# Injury-transduced oligodendrocytes modulate neuroinflammation and glial activation in diseased and non-diseased central nervous system

**DOI:** 10.1038/s41467-026-76159-2

**Published:** 2026-08-29

**Authors:** Yan Wang, Meina Zhu, Joohyun Park, Hyun Kyu Kim, Ge Xiong, Xi Chen, Bo Chen, Shinghua Ding, Wensheng Lin, Fuzheng Guo

**Affiliations:** 1 Department of Neurology, School of Medicine, University of California, Davis, Sacramento, CA USA; 2 Institute for Pediatric Regenerative Medicine (IPRM), Shriners Hospitals’ for Children – Northern California, Sacramento, CA USA; 3 Institute of Neurology, Sichuan Provincial People’s Hospital, Chengdu, China; 4 School of Medicine, University of Electronic Science and Technology of China, Chengdu, China; 5 Department of Neuroscience, Cell Biology and Anatomy, University of Texas Medical Branch, Galveston, TX USA; 6 Department of Chemical and Biomedical Engineering, University of Missouri, Columbia, MO USA; 7 Department of Neuroscience, University of Minnesota, Minneapolis, MN USA

**Keywords:** Oligodendrocyte, Cellular neuroscience

## Abstract

Oligodendrocytes (OLs) are brain cells that make myelin, the insulating sheath that supports nerve signal transmission. Although oligodendrocyte dysfunction is common in the central nervous system (CNS), how these cells respond to injury remains incompletely understood. Here we show, using mouse models and mouse tissue analyses, that OLs respond to demyelinating diseases by increasing the expression and secretion of serine protease inhibitor clade A member 3N (SERPINA3N). This transition of homeostatic OLs to Serpina3n-expressing OLs (SerpinOLs) occurs not only in demyelinating disease, but also after stroke, endotoxin-induced injury, neurodegeneration, traumatic injury, and healthy aging. Mechanistically, direct injury to OLs, rather than inflammation alone, drives the transition. Phenotypically, SerpinOLs show inflammatory and immune-regulatory features and activation of signal transducer and activator of transcription 3 (STAT3), which is required for SERPINA3N induction. Functionally, SerpinOLs amplify neuroinflammation and glial activation toward pro-inflammatory and neurodegenerative states. Together, SerpinOLs represent a common population of injury-transduced OLs that contributes to CNS pathology beyond myelin production.

## Abstract (web summary)

This study identifies SerpinOLs, injury-responsive oligodendrocytes producing SERPINA3N, as a common cell state in disease and aging, which amplify neuroinflammation and glial activation beyond myelin-related functions in mice.

## Introduction

Oligodendrocytes (OLs), generated from oligodendroglial progenitor cells (OPCs), are myelin-forming cells of the central nervous system (CNS). Oligodendroglial lineage cells (OLs and OPCs) support neuron/axonal survival and shape neural circuitry through developmental myelination in the developing brain^1^ and adaptive myelination in the adult brain^2^, both of which are essential for brain function and behavior^3^. OLs and their myelin derivatives are the primary victims of demyelinating disorders such as multiple sclerosis, an inflammatory CNS demyelinating disease. In addition, oligodendrocyte dysfunction has been well-recognized in other CNS pathologies^4^ such as stroke^5^, endotoxicity^6^, neurodegeneration^7^, neurotrauma^8^, and even normal (or healthy) aging, a naturally occurring process in the absence of diseases or trauma^9^. Despite the commonly existing dysfunction, how OLs respond to CNS pathologies molecularly and functionally remains incompletely understood.

Single-cell transcriptomics and meta-analysis have unveiled diverse subpopulations of disease-associated or specific oligodendroglia (DOLs)^10–14^ in the diseased CNS. They are defined by different transcriptomic signatures, for example, DOLs defined by signature genes associated with immunogenesis, differentiation and survival, and interferon response^11,15^. There is no doubt that more DOL subpopulations will be identified with the technical advancement of next-generation sequencing and bioinformatics. However, there are at least two fundamental knowledge gaps for the ever-growing DOL subpopulations. First, the cellular and molecular mechanisms that transition homeostatic OLs into distinct activation states are still enigmatic. Second, the biological functions of DOLs in disease pathophysiology remain unknown or largely speculative in vivo^10,16–18^.

In the present study, we found that homeostatic OLs respond to inflammatory demyelination by secreting serine protease inhibitor clade A member 3N (SERPINA3N), a secretory protein dysregulated in the brain and body fluids in many neurological conditions^19^. SERPINA3N-expressing OLs (termed SerpinOLs) are present in various types of CNS neurological conditions. Using a series of transgenic mice and injury models, we show that direct intrinsic injury to OLs, regardless of the presence of neuroinflammation (a common feature of CNS pathologies), triggers the conversion of homeostatic OLs into SerpinOLs. Using SERPINA3N mutant transgenic mice, we demonstrate that SerpinOLs perpetuate CNS inflammatory response and promote glial activation toward pro-inflammatory and neurodegenerative states in the diseased and aged CNS. Thus, our study defines SerpinOLs as an injury-transduced population of OLs that serve as crucial modifiers for neuroinflammation and glial activation, common pathological features in various neurological diseases and injuries. Our findings suggest OLs actively participate in CNS pathology regulation through myelination-independent pathways.

## Results

### Screening for demyelination-responsive genes identifies *Serpina3n* dysregulation selectively in oligodendrocytes

**Fig. 1:**
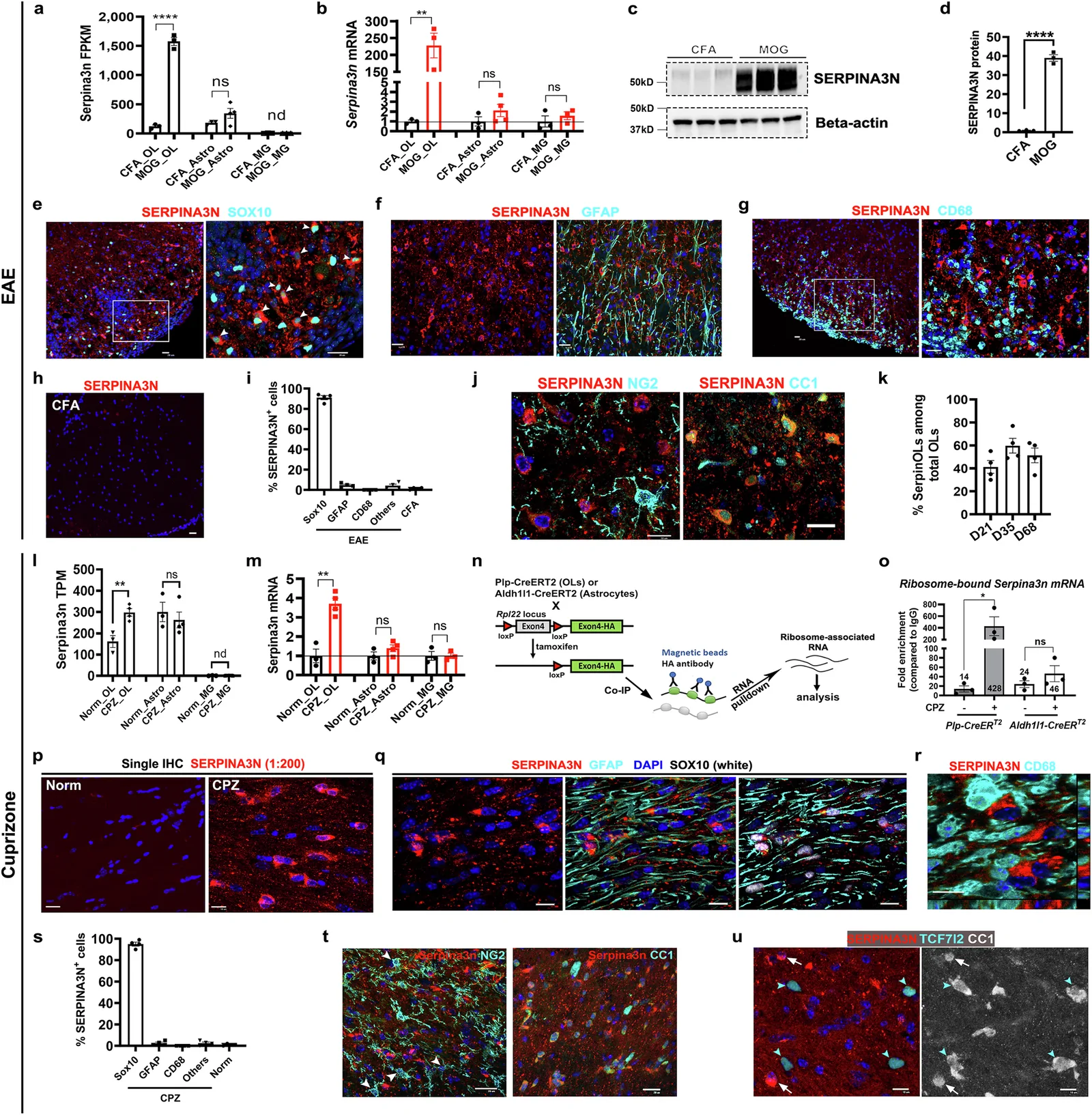
Oligodendrocytes transition into SERPINA3N-expressing OLs (SerpinOLs) in response to CNS demyelination. RNA-seq (**a**) and RT-qPCR (**b**) analysis of *Serpina3n* in MACS-purified oligodendroglia (OL), astroglia (Astro), and microglia (MG) from D30 MOG/EAE and CFA Ctrl spinal cords. nd, not detectable. **a** OL, ****P < 0.0001; Astro, *P* = 0.1684; MG, *P* = 0.2453. **b** OL, **P = 0.0035; Astro, *P* = 0.2404; MG, *P* = 0.4565. *n* = 3 mice except MOG_Astro, *n* = 4 mice. Western blot (**c**) and quantification (**d**) of SERPINA3N in D21 MOG/EAE and CFA spinal cords. ****P < 0.0001. *n* = 3 mice. β-actin run on a separate gel using the same samples is a sample processing control. Fluorescent immunohistochemistry (IHC) for SERPINA3N with SOX10 (**e**), GFAP (**f**), or CD68 (**g**) in D21 MOG/EAE and CFA Ctrl (**h**) spinal cord ventral white matter and quantification by lineage markers (**i**). Arrowheads, intracellular SOX10⁺SERPINA3N⁺ cells. Boxed areas, higher magnification. IHC of SERPINA3N and NG2 (OPCs) or CC1 (mature OLs) (**j**) and quantification (**k**). **e**–**k** *n* = 4 mice. RNA-seq (**l**) and RT-qPCR (**m**) of Serpina3n in MACS-purified OL, Astro and MG from 4-week CPZ or Norm brains. **l** OL, **P = 0.0075; Astro, *P* = 0.5503; MG, *P* = 0.6745. **m** OL, **P = 0.0019; Astro, *P* = 0.2039; MG, *P* = 0.9847. *n* = 3 mice Norm_OL, Norm_Astro, Norm_MG and CPZ_MG; *n* = 4 mice CPZ_OL and CPZ_Astro. **n** RiboTag strategy. **o** RT-qPCR of ribosome-bound *Serpina3n* transcripts. *P = 0.0427, ns, *P* = 0.9971. *n* = 3 mice per group. **p** IHC of SERPINA3N in the corpus callosum (CC) of Norm and 4-week CPZ mice. **q** IHC of SERPINA3N, SOX10, and GFAP. **r** Orthogonal view of confocal images of SERPINA3N and CD68. **s** % SERPINA3N^+^ cells co-expressing the indicated markers. *n* = 4 mice per group. **t** IHC of SERPINA3N and NG2 or CC1. Arrowheads, NG2^+^ OPCs. **u** IHC for SERPINA3N, CC1, and newly regenerated OL marker TCF7l2. Cyan arrowheads, CC1^+^TCF7l2^+^SERPINA3N^-^ cells; red arrowheads, CC1^+^TCF7l2^-^SERPINA3N^+^ cells. Scale bars: **e**–**h**, **j**, **p**–**r**, **u** 10 µm, **t** 20 µm. Data are mean ± SEM. **a**, **b**, **d**, **l**, **m** Two-tailed unpaired Student’s *t*-test; **o** two-way ANOVA with Tukey’s test. Source data are provided as a Source Data file.

We set out to identify genes responsive to inflammatory demyelination by conducting an unbiased RNA-seq analysis of MOG/EAE mice (Supplementary Fig. 1a), an animal model of multiple sclerosis^20^. Most top differentially expressed genes (DEGs), including *Gpnmb*, *Trem2*, *Lyz2*, *Ctsd*, and *Cd68*, were enriched in activated microglia/macrophages (Supplementary Fig. 1b). In contrast, *Serpina3n*, the third most highly expressed DEG in the top 10 list (Supplementary Data 1), was selectively induced in oligodendrocytes (OLs) (Fig. 1a), as shown by glial type-specific RNA-seq analysis (Supplementary Fig. 1c–f). Among OL-specific DEGs, *Serpina3n* showed the strongest induction in response to MOG/EAE (Supplementary Data 2), a result confirmed by real-time quantitative PCR (RT-qPCR) (Fig. 1b). These findings identify *Serpina3n* as a responsive gene to inflammatory demyelination specifically in OLs.

### SERPINA3N is dysregulated and secreted by oligodendrocytes during autoimmune demyelination

To determine whether *Serpina3n* mRNA induction results in protein upregulation, we performed Western blot (Fig. 1c), which revealed a > 30-fold increase in SERPINA3N protein in the spinal cord of MOG/EAE mice (Fig. 1d). Fluorescent immunohistochemistry (IHC) was used to characterize the cellular specificity of SERPINA3N. Not only intracellular but also extracellular SERPINA3N was detected in the MOG/EAE spinal cord (Fig. 1e–g), consistent with its secretory properties^19^. SERPINA3N^+^ cells were predominantly identified as SOX10^+^ oligodendroglial lineage cells (Fig. 1e, i), and few were GFAP^+^ astrocytes (Fig. 1f, i) or CD68^+^ activated microglia/macrophages (Fig. 1g, i). In CFA-treated control mice, SERPINA3N expression was minimal (Fig. 1h, i). All SERPINA3N^+^ oligodendroglial lineage cells were identified as CC1^+^ mature OLs but not NG2^+^ OPCs (Fig. 1j). These findings suggest that mature OLs upregulate and secrete SERPINA3N in response to inflammatory demyelination.

SERPINA3N-expressing OLs, which we referred to as SerpinOLs, persisted into the chronic phase of MOG/EAE (Supplementary Fig. 2a–c) when peripheral inflammatory infiltrates subside^21^, indicating that peripheral immune cell infiltration is dispensable for SERPINA3N expression. SerpinOLs were found in both lesional (Supplementary Fig. 2b) and non-lesional regions (Supplementary Fig. 2b). SerpinOLs were negative for TCF7l2 (Supplementary Fig. 2b), a nuclear marker labeling newly regenerated OLs^22^, suggesting that they are not newly formed OLs. Many SerpinOLs were located in proximity to CD68^+^ cells (Supplementary Fig. 2d). At the population level, SerpinOLs accounted for ~40–60% of all mature OLs during the disease course of MOG/EAE (Fig. 1k). Together, our data suggest that OLs, but not other cell types, respond to inflammatory demyelination via SERPINA3N induction.

### Chemically induced demyelination transitions homeostatic OLs into SerpinOLs

We next tested whether OLs are the major cell-type secreting SERPINA3N in the cuprizone (CPZ) demyelination model, which was characterized by the local expansion and activation of resident microglia with a limited number of monocyte-derived macrophages (Ccr2-RFP^+^) (Supplementary Fig. 3a). Glial type-specific RNA-seq (Supplementary Fig. 3b, c, Supplementary Data 3) showed a significant increase of *Serpina3n* transcripts only in OLs (Fig. 1l), a finding validated by RT-qPCR assay (Fig. 1m) although astrocytes exhibited similar levels of *Serpina3n* transcripts to OLs (Fig. 1l). We used the RiboTag technique^23^ to determine if *Serpina3n* transcripts are translated into SERPINA3N proteins in astrocytes and/or OLs (Fig. 1n). Our results revealed a >30-fold increase in ribosome-bound *Serpina3n* mRNA in OLs, but not astrocytes (Fig. 1o) during CPZ demyelination. This finding suggests that the active translation of SERPINA3N protein occurs predominantly in OLs during CPZ demyelination.

SERPINA3N showed a splenium-to-genu gradient in the corpus callosum of CPZ-treated mice (Supplementary Fig. 3d), mirroring the caudal-to-rostral gradient of oligodendrocyte/myelin damage in this model^24^. Both intracellular and extracellular SERPINA3N signal was present in CPZ-treated mice, whereas SERPINA3N was barely detectable in normal diet control (Fig. 1p). Greater than 90% of intracellular SERPINA3N^+^ cells were positive for SOX10 and few, if any, were GFAP (Fig. 1q, s) or CD68 (Fig. 1r, s), identifying them as oligodendroglial lineage cells. All SERPINA3N^+^ oligodendroglial lineage cells were further identified as CC1^+^ mature OLs, but not NG2^+^ OPCs (Fig. 1t) or TCF7l2^+^ newly generated OLs^22^ (Fig. 1u). Paradoxically, extensive co-labeling of SERPINA3N with GFAP^+^ reactive astrocytes was noticed only on fluorescent double IHC of SERPINA3N/GFAP (Supplementary Fig. 3e), an observation also reported in previous studies^25,26^. The observed co-labeling likely resulted from optical artifacts because the process-like SERPINA3N immunoreactive signal (Supplementary Fig. 3e) was absent from the single IHC of SERPINA3N (Fig. 1p), triple IHC of SERPINA3N/GFAP/SOX10 (Fig. 1q, Supplementary Fig. 3f), or sequential staining of SERPINA3N and GFAP (and the reverse order) (Supplementary Fig. 3g, h). Collectively, our results show that OLs upregulate and secrete SERPINA3N in response to CNS demyelination regardless of peripheral immune infiltration.

### Genetic evidence demonstrating oligodendrocyte-specific SERPINA3N upregulation and secretion during demyelinating injury

**Fig. 2:**
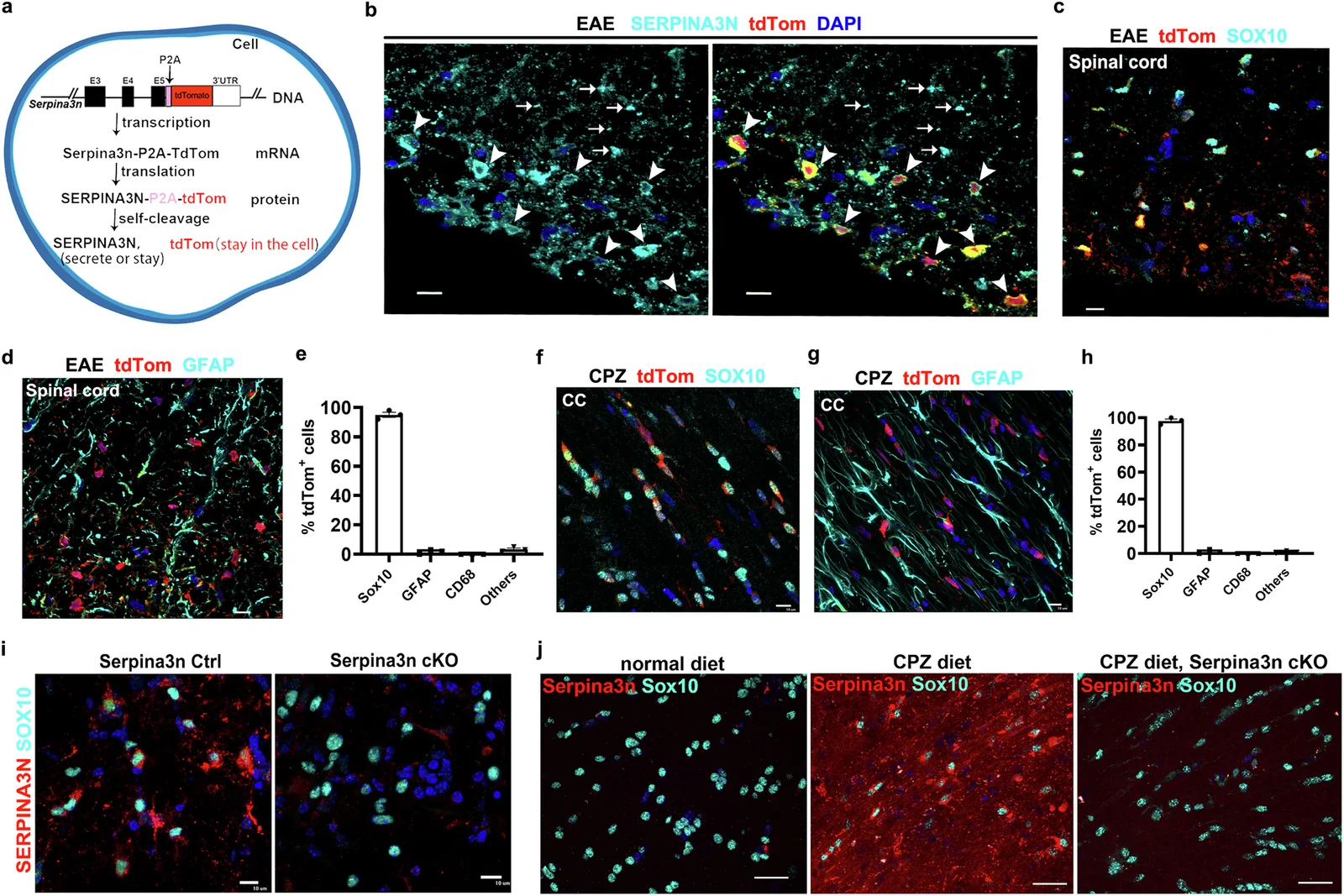
Genetic evidence for SERPINA3N expression in oligodendrocytes. **a** Schematic of *Serpina3n*-tdTom reporter design. The P2A self-cleaving site separates dTom from SERPINA3N-tdTom, allowing tdTom to localize in cell bodies while SERPINA3N remains intracellular or is secreted. **b** Double fluorescent IHC for SERPINA3N and tdTom in D30 MOG/EAE ventral white matter of spinal cord. Arrowheads, tdTom^+^ SERPINA3N^+^ cell bodies; arrows, extracellular SERPINA3N. IHC of tdTom and SOX10 (**c**) or GFAP (**d**) in D30 MOG/EAE spinal cord. **e** Quantification of tdTom^+^ cells co-expressing the indicated marker in D30 MOG/EAE spinal cord. *n* = 3 mice per group. IHC of tdTom and SOX10 (**f**) or GFAP (**g**) in 3-week CPZ corpus callosum (CC). **h** Quantification of tdTom^+^ cells co-expressing indicated markers in 3-week CPZ corpus callosum. *n* = 3 mice per group. **i** IHC for SERPINA3N and SOX10 in spinal cord of *Serpina3n* control (Ctrl) and conditional knockout (cKO, *Olig2-Cre:Serpina3n*^*fl/fl*^) mice at D30 post-MOG/EAE. **j** IHC of SERPINA3N and SOX10 in the corpus callosum of *Serpina3n* Ctrl and cKO mice after a 4-week CPZ (or Norm) diet. Scale bars: **b**–**d**, **f**, **g**, **i** 10 µm, **j** 20 µm. Data are presented as mean values ± SEM. Source data are provided as a Source Data file.

Due to its secretory nature, SERPINA3N in OLs may originate from other cell types and be deposited in OLs. No direct evidence exists proving that SERPINA3N protein originates from OLs. To study the cellular origin of SERPINA3N, we generated *Serpina3n-*tdTom reporter mice (Fig. 2a, Supplementary Fig. 4a–d). The induction of SERPINA3N-driven tdTom expression was validated using MOG/EAE and CPZ models (Supplementary Fig. 4e, f). In the MOG/EAE spinal cord, tdTom was co-localized with intracellular SERPINA3N (Fig. 2b, arrowheads), confirming the efficacy of tdTom in reporting SERPINA3N expression. Over 90% of tdTom^+^ cells were SOX10^+^ oligodendroglial lineage, with a negligible fraction being GFAP^+^ astroglial lineage cells or CD68^+^ activated microglia/macrophages (Fig. 2c–e). Similarly, in the corpus callosum of CPZ-demyelinating mice, >90% of tdTom^+^ cells were identified as SOX10^+^ and few were GFAP^+^ or CD68^+^ cells (Fig. 2f–h), confirming that OLs but not astrocytes or microglia are the cellular origin of SERPINA3N protein. To further corroborate our conclusion, we generated oligodendroglia-specific *Serpina3n* conditional knockout (cKO) mice (*Olig2-Cre:Serpina3n*^*fl/fl*^)^27^. In these mice, SERPINA3N immunoreactivity, both intracellular and extracellular, was nearly abolished in the spinal cord of MOG/EAE mice (Fig. 2i) and in the corpus callosum of CPZ mice (Fig. 2j). Thus, these data provide conclusive evidence demonstrating that OLs are the primary source of SERPINA3N expression and secretion in response to CNS demyelination.

### Diverse CNS pathologies induce the transition of homeostatic OLs into SerpinOLs

Previous studies have identified *Serpina3n* as a marker of reactive astrocytes in diseased conditions such as ischemic stroke^28^, lipopolysaccharide (LPS)-induced neuroinflammation^28^, Alzheimer’s disease (AD)^29^, and non-diseased normal aging^30^. However, our findings in demyelination models prompted us to re-evaluate this widely cited concept. We hypothesize that OLs are the primary source of SERPINA3N in these CNS pathologies.

To test this, we examined SERPINA3N expression in multiple CNS disease and injury paradigms. In photothrombotic ischemic stroke^31^ (Supplementary Fig. 5), SERPINA3N⁺ cells peaked at ~7 dpi (days post-injury) in the penumbra and were largely SOX10⁺ oligodendroglial lineage, with minimal contribution from astrocytes or myeloid cells. Similarly, following LPS-induced neuroinflammation (Supplementary Fig. 6), the majority of SERPINA3N⁺ (or *Serpina3n*-tdTom⁺) cells were SOX10⁺ oligodendroglial lineage. This oligodendroglial lineage specificity was rigorously corroborated using *Serpina3n*-tdTom transgenic reporter mice, ruling out non-specificity of the SERPINA3N antibody (Supplementary Fig. 6d1–e3). Similarly, in the 5xFAD model of AD (Supplementary Fig. 7 and 8), while extracellular SERPINA3N deposits co-localized with amyloid plaques, intracellular SERPINA3N was almost exclusively restricted to SOX10^+^ oligodendroglial lineage (>90%) in the dorsal subiculum and fimbria, regions enriched with amyloid plaques in the model. Importantly, we demonstrated that previously reported astrocyte-specific SERPINA3N signal in AD model^29^ may be an optical artifact inherent to simultaneous double-staining protocols because sequential and triple immunohistochemistry confirmed that astrocytes express negligible levels of SERPINA3N. Finally, in a spinal cord injury model (SCI, Supplementary Fig. 9), SERPINA3N^+^ cells are primarily localized in the white matter below the lesion site and identified as SOX10^+^ oligodendroglial lineage. Collectively, these data challenge the prevailing dogma^28^ and establish that the transition of homeostatic oligodendroglia into a SERPINA3N-expressing state (SerpinOLs) is a conserved response across diverse neurological injuries and diseases.

### Normal aging transitions homeostatic OLs into SerpinOLs

**Fig. 3:**
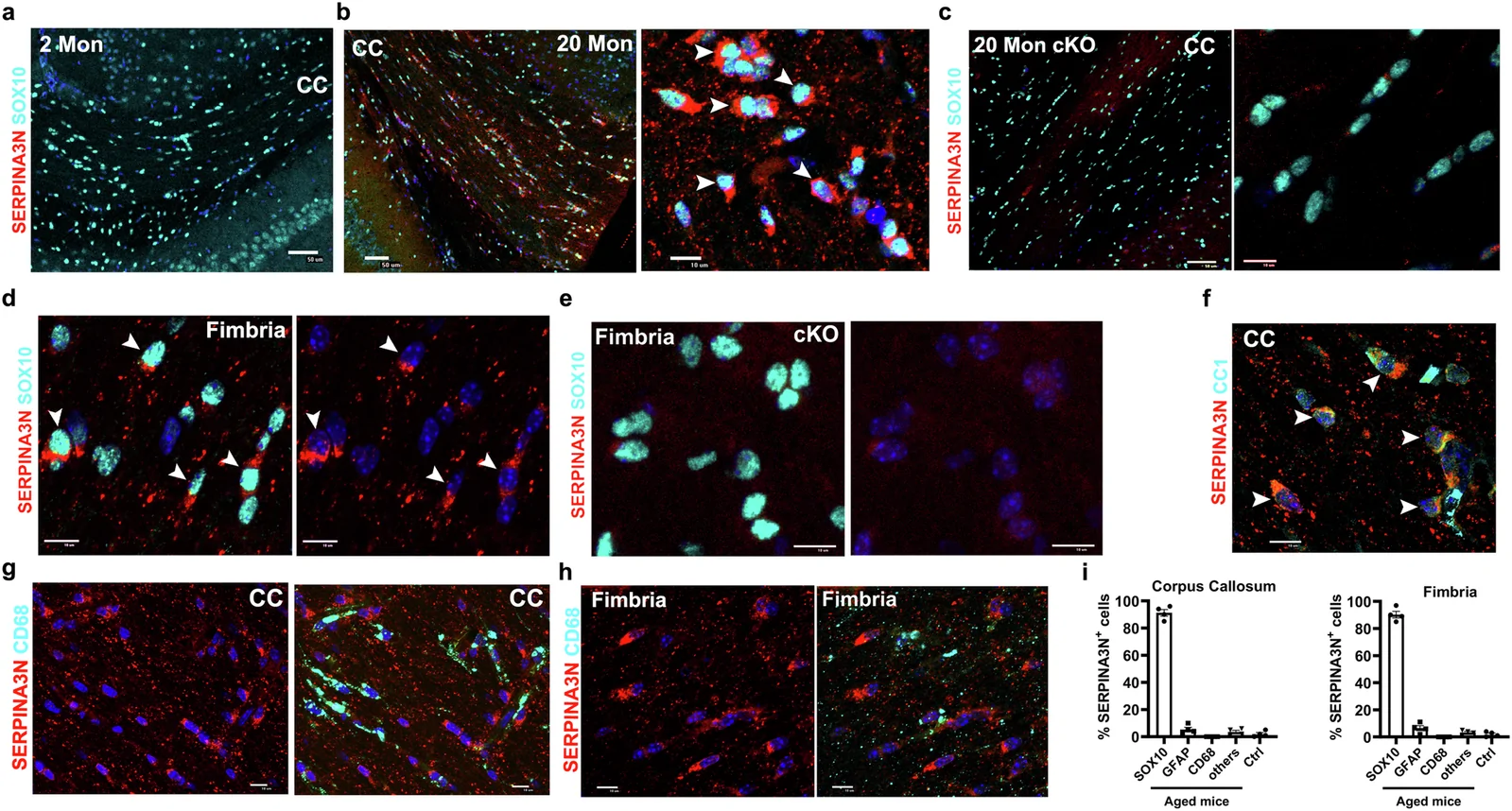
SerpinOLs are present in the brain during normal aging. Double IHC of SERPINA3N and SOX10 in the corpus callosum (CC) of young (2 months, Mon, **a**), aged (20 months, **b**), and aged *Serpina3n* cKO mice (**c**). Arrowheads indicate SERPINA3N^+^SOX10^+^ cells. Numerous SERPINA3N puncta were observed in aged mice and were abolished in aged *Serpina3n* cKO mice. SERPINA3N^+^SOX10^+^ SerpinOLs (arrowheads) in the fimbria of aged WT (**d**) and *Serpina3n* cKO (**e**) mice. **f** Confocal image showing SERPINA3N^+^ cells co-labeled with CC1^+^ mature OLs in aged CC. Fluorescent IHC showing absence of SERPINA3N from CD68^+^ cells in aged CC (**g**) and fimbria (**h**). **i** Percentage of SERPINA3N^+^ cells positive for indicated markers in aged and young Ctrl mice. *n* = 4 mice. Scale bars: **a** 50 µm, **d**–**h** 20 µm. Data are presented as mean values ± SEM. Source data are provided as a Source Data file.

We next examine whether normal healthy aging, a naturally occurring process in the absence of diseases or neurotraumas^9^, is sufficient to convert homeostatic OLs into SerpinOLs. While homeostatic OLs do not express SERPINA3N in the young adult brain (Fig. 3a), we observed robust induction of intracellular and extracellular SERPINA3N in the aged brain (20 months old), predominantly in subcortical white matter tracts, such as the corpus callosum (Fig. 3b) and fimbria (Fig. 3d), consistent with recent findings that white matter is the most vulnerable hotspot of brain aging^32^. Approximately 90% of intracellular SERPINA3N^+^ cells were identified as SOX10^+^ oligodendroglial lineage (Fig. 3b, d, arrowheads, Fig. 3i). Further analysis confirmed that they were CC1^+^ mature OLs (Fig. 3f, arrowheads).

We generated *Olig2-Cre:Serpina3n*^*fl/fl*^ mice (oligodendroglial *Serpina3n* cKO) to define whether OLs are the cellular source of SERPINA3N during normal aging. We found that SERPINA3N immunoreactive signal, both intracellular and extracellular, was abolished in the corpus callosum (Fig. 3c) and fimbria (Fig. 3d) of aged *Serpina3n* cKO mice compared with controls, validating OLs as the primary producers of SERPINA3N in the aged brain.

Normal aging is associated with chronic activation of microglia^33^. We found that activated microglia (CD68^+^) were frequently observed in the regions of the corpus callosum (Fig. 3g) and fimbria (Fig. 3h) where SerpinOLs were markedly increased. Collectively, our results establish that normal aging transitions homeostatic OLs into SerpinOLs. The proximity of SerpinOLs to activated microglia (Fig. 3g, h, arrowheads) indicates that normal aging promotes SerpinOL transition preferentially in white matter regions with microglial activation and that SerpinOLs may modulate microglial activation in the aged brain.

### Neuroinflammation is insufficient to transition homeostatic OLs into SerpinOLs

The mechanisms underlying the transition of homeostatic OLs into SerpinOLs remain poorly understood. Given that neuroinflammation is a shared hallmark of the neurological conditions assessed, we tested if neuroinflammation is sufficient to transition homeostatic OLs into SerpinOLs. To this end, we employed two independent experimental approaches.

**Fig. 4:**
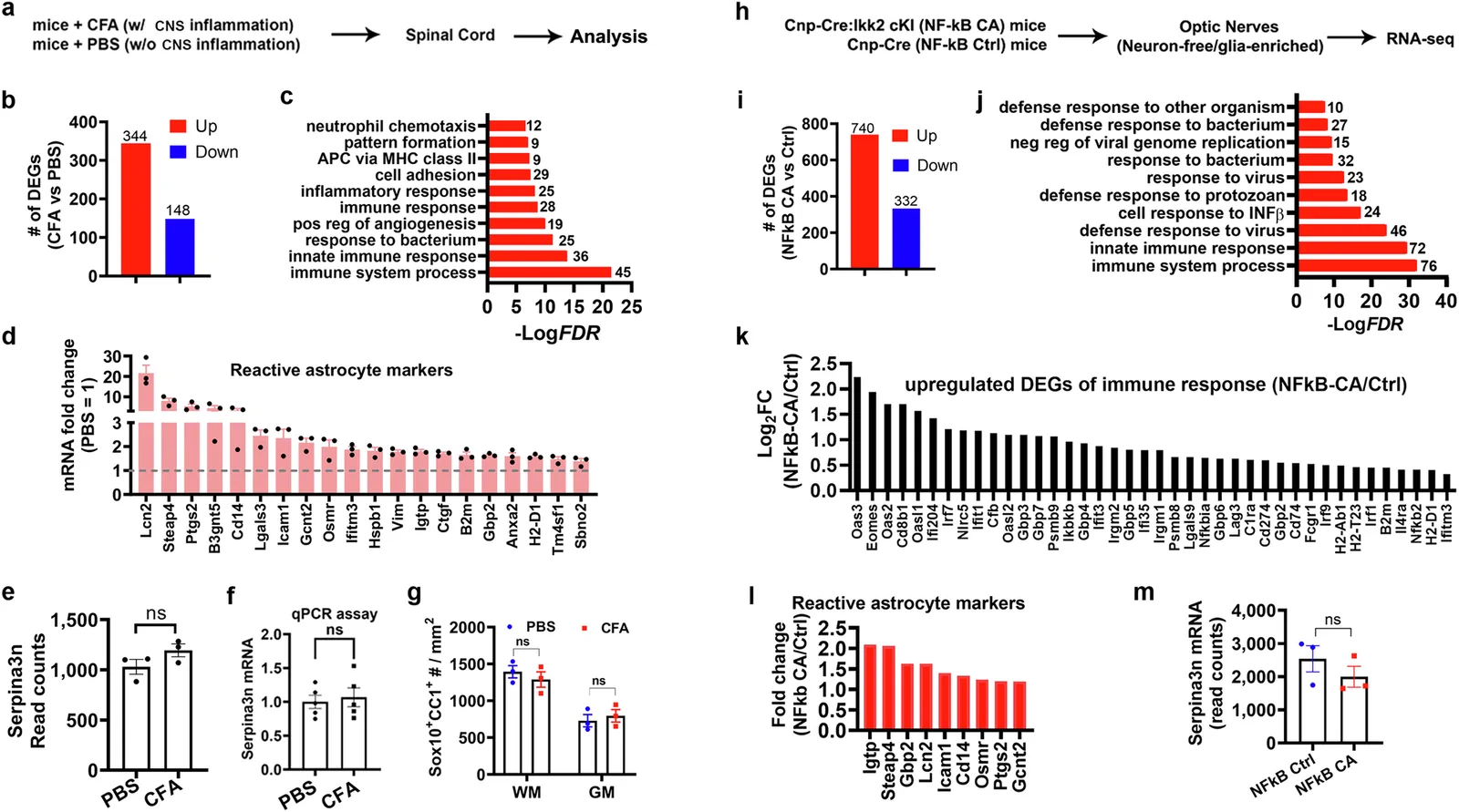
CNS inflammation is not sufficient but promotes SERPINA3N expression in OLs. **a** Experimental design for (**b**–**g**). Adult mice were immunized with CFA or PBS (s.c. day 0) and pertussis toxin (i.p. days 0 and 2). Spinal cords were analyzed at day 35 post-CFA (or PBS). **b** Number of up- or downregulated DEGs in CFA vs. PBS spinal cord identified by RNA-seq (see Supplementary Data 4). **c** Top 10 enriched Gene Ontology (GO) terms overrepresented by upregulated DEGs in CFA vs. PBS (Supplementary Data 4). **d** Upregulation of reactive astrocyte marker genes in CFA vs. PBS. *n* = 3 mice per group. **e** *Serpina3n* RNA-seq read counts. *P* = 0.1674. *n* = 3 mice per group. **f** RT-qPCR of *Serpina3n* mRNA. *P* = 0.7005. *n* = 5 mice per group. **g** Densities of OLs (SOX10^+^CC1^+^) in spinal cord white matter (WM) and gray matter (GM). *P* = 0.4753 WM, *P* = 0.6081 GM. *n* = 3 mice per group. **h** Experimental design for (**i**–**m**). RNA-seq was performed on optic nerves from oligodendroglial-specific NFkB constitutive activation (NFkB CA) and Ctrl mice. **i** Number of DEGs in NFkB CA vs. Ctrl mice identified by RNA-seq (Supplementary Data 5). **j** Top 10 enriched GO terms overrepresented by upregulated DEGs in NFkB CA (Supplementary Data 5). **k** Examples of immune response genes upregulated in NFkB CA (Supplementary Data 5). **l** Upregulation of reactive astrocyte marker genes in NFkB CA vs. Ctrl mice. **m** *Serpina3n* RNA-seq read counts. *P* = 0.3459. *n* = 3 mice per group. Data are presented as mean values ± SEM. Statistical significance was determined using a two-tailed unpaired Student’s *t*-test. Source data are provided as a Source Data file.

In the first approach, mice were inoculated with complete Freund’s adjuvant (CFA), a potent stimulator of the innate immune system^34^, to elicit CNS inflammation and glial activation. Unbiased RNA-seq analysis (Fig. 4a) revealed robust inflammatory responses in the spinal cord of CFA-treated mice, as evidenced by the induction of a large cohort of DEGs (Fig. 4b, Supplementary Data 4) related to immune system process, innate immune response, and bacterial responses (Fig. 4c). CFA also triggered astrocyte activation, as shown by the upregulation of reactive astrocyte markers (Fig. 4d). Despite these marked inflammatory and glial responses, *Serpina3n* transcripts were not induced in the spinal cord of CFA-treated mice compared with PBS controls (Fig. 4e, f). We found that CFA treatment did not cause oligodendroglial damage or loss (Fig. 4g). These data suggest that neuroinflammation, in the absence of OL injury, is insufficient to transition homeostatic OLs into SerpinOLs.

In the second approach, we used a genetic model to induce CNS inflammation. We generated *Cnp-Cre:R26Stop*^*FL*^*ikk2ca* transgenic mice (referred to as NFkB CA mice) in which NFkB signaling, a key regulator of inflammation^35^, is constitutively activated in oligodendroglial lineage cells^36^. Our previous studies showed that NFkB activation does not affect oligodendroglial viability under normal conditions^36^. Our analysis of glial cell-enriched optic nerves (Fig. 4h) revealed marked transcriptomic changes (Fig. 4i, Supplementary Data 5), including upregulation of genes associated with immune system processes and innate immune response (Fig. 4j, k) and astroglial activation (Fig. 4l), compared with NFkB Ctrl mice. Despite this robust inflammatory environment, Serpina3n was not induced in NFkB CA mice (Fig. 4m), further supporting the conclusion that CNS inflammation alone does not drive the transition of homeostatic OLs into SerpinOLs. These results suggest that additional factors, such as OL injury or damage, play crucial roles in this transition.

### CNS inflammation potentiates SERPINA3N expression under demyelinating conditions

To assess whether inflammation enhances SERPINA3N expression in the context of demyelinating injury, we induced MOG/EAE in adult NFkB CA and Ctrl mice (Supplementary Fig. 10a). Elevated NFkB activity in NFkB CA mice was confirmed by increased phospho-p65 levels (Supplementary Fig. 10b). While the density of SERPINA3N^+^ cells (Supplementary Fig. 10c, d) and the proportion of SerpinOLs among total OLs (Supplementary Fig. 10e) were comparable between NFkB CA and Ctrl groups, NFkB CA mice exhibited a significant increase in SERPINA3N-occupying area (Supplementary Fig. 10f) and SERPINA3N signal intensity (Supplementary Fig. 10g) compared with Ctrl mice. These findings demonstrate that although neuroinflammation alone does not initiate the SerpinOL state transition, it amplifies SERPINA3N expression in SerpinOLs under demyelinating conditions. Given extensive OL damage/injury in MOG/EAE, our data suggest that oligodendroglial injury may be a key driver for the state transition from homeostatic OLs to SerpinOLs.

### Oligodendroglial injury drives the transition of homeostatic OLs into SerpinOLs

**Fig. 5:**
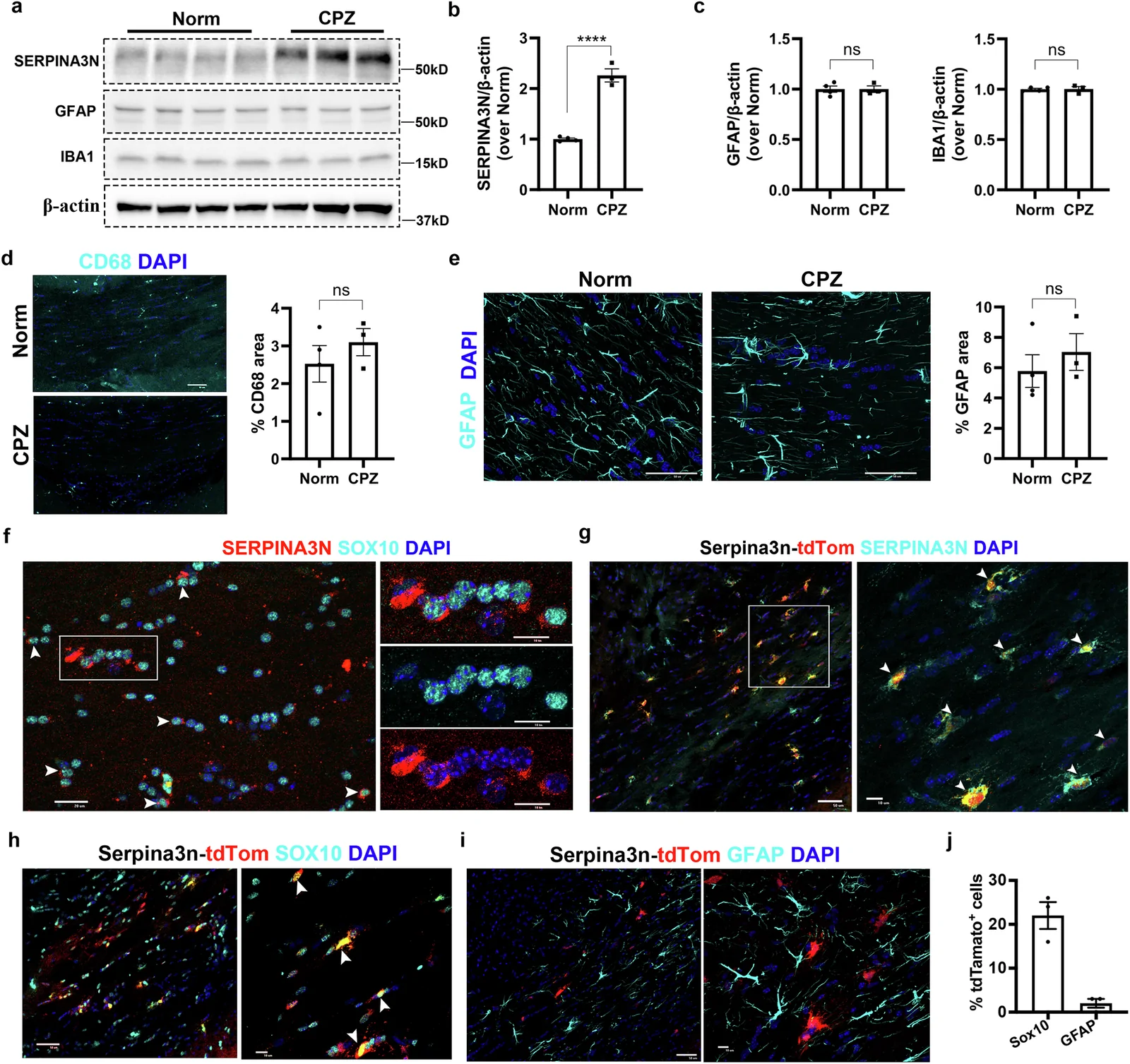
OL injury is sufficient for SerpinOL transformation. **a**–**c** Western blot and quantification of SERPINA3N, GFAP, and IBA1 in brains of adult mice after 2 days of normal (Norm) or cuprizone (CPZ) diet. ****P < 0.0001 SERPINA3N; *P* = 0.9916 GFAP; *P* = 0.9097 IBA1. *n* = 4 mice for Norm; *n* = 3 mice for CPZ. β-actin was run on a separate gel using the same samples and is shown as a sample processing control. IHC and quantification of CD68^+^ (**d**) and GFAP^+^ cells (**e**) in the corpus callosum after 2 days of CPZ. *P* = 0.4172 CD68; *P* = 0.4748 GFAP. *n* = 4 mice for Norm; *n* = 3 mice for CPZ. **f** IHC of SERPINA3N and SOX10 in the corpus callosum after 2 days of CPZ. Arrowheads indicate SERPINA3N^+^ OLs. Boxed region shown at higher magnification. **g** IHC of SERPINA3N and tdTom in the corpus callosum of *Serpina3n*-tdTom mice on a CPZ diet for 2 days. Boxed region shown at higher magnification. Arrowheads point to double-positive cells. IHC of tdTom with SOX10 (**h**) or GFAP(**i**) in the corpus callosum of *Serpina3n*-tdTom mice on a CPZ diet for 2 days. **j** Quantification of the percentage of tdTomato^+^ cells co-expressing SOX10 or GFAP after 2 days of CPZ. *n* = 3 mice for CPZ. Scale bars: **d**, **e** 50 µm, **f** 20 µm, **g**–**I** 50 µm (lower magnification) and 10 µm (higher magnification). Data are presented as mean values ± SEM. Statistical significance was determined using a two-tailed unpaired Student’s *t*-test. Source data are provided as a Source Data file.

To determine whether OL injury is sufficient to trigger the transition of homeostatic OLS into SerpinOLs, we employed the CPZ demyelination model. Previous studies have shown that CPZ consumption induces rapid OL injury and loss in the corpus callosum as early as day 2^37^. At this early timepoint, we observed a significant increase in SERPINA3N expression in the brain (Fig. 5a, b), prior to detectable activation of microglia or astrocytes, as indicated by unaltered levels of IBA1, CD68, and GFAP at both protein and histological levels (Fig. 5a–e). Notably, SERPINA3N expression was detected in SOX10^+^ cells exhibiting beads-on-a-string morphology (Fig. 5f, boxed area), a characteristic of normal OLs in the corpus callosum under homeostatic conditions. To corroborate these findings, we utilized *Serpina3n*-tdTom reporter mice. Robust tdTom was co-localized with endogenous SERPINA3N at day 2 post-CPZ diet (Fig. 5g), confirming the accuracy of the reporter in marking SERPINA3N-producing cells. We found that approximately 20% of SOX10^+^ oligodendroglial lineage cells transitioned into tdTom^+^ SerpinOLs in the corpus callosum at this stage (Figs. 5h, j). Consistent with our findings in 4-week CPZ (Fig. 1q, Fig. 2g), minimal tdTom expression was detected in GFAP^+^ astrocytes at 2 days CPZ (Figs. 5i, j). These results suggest that oligodendroglial injury alone is sufficient to drive the transition of homeostatic OLs into SerpinOLs.

### Cell-autonomous OL injury triggers SerpinOL transition

**Fig. 6:**
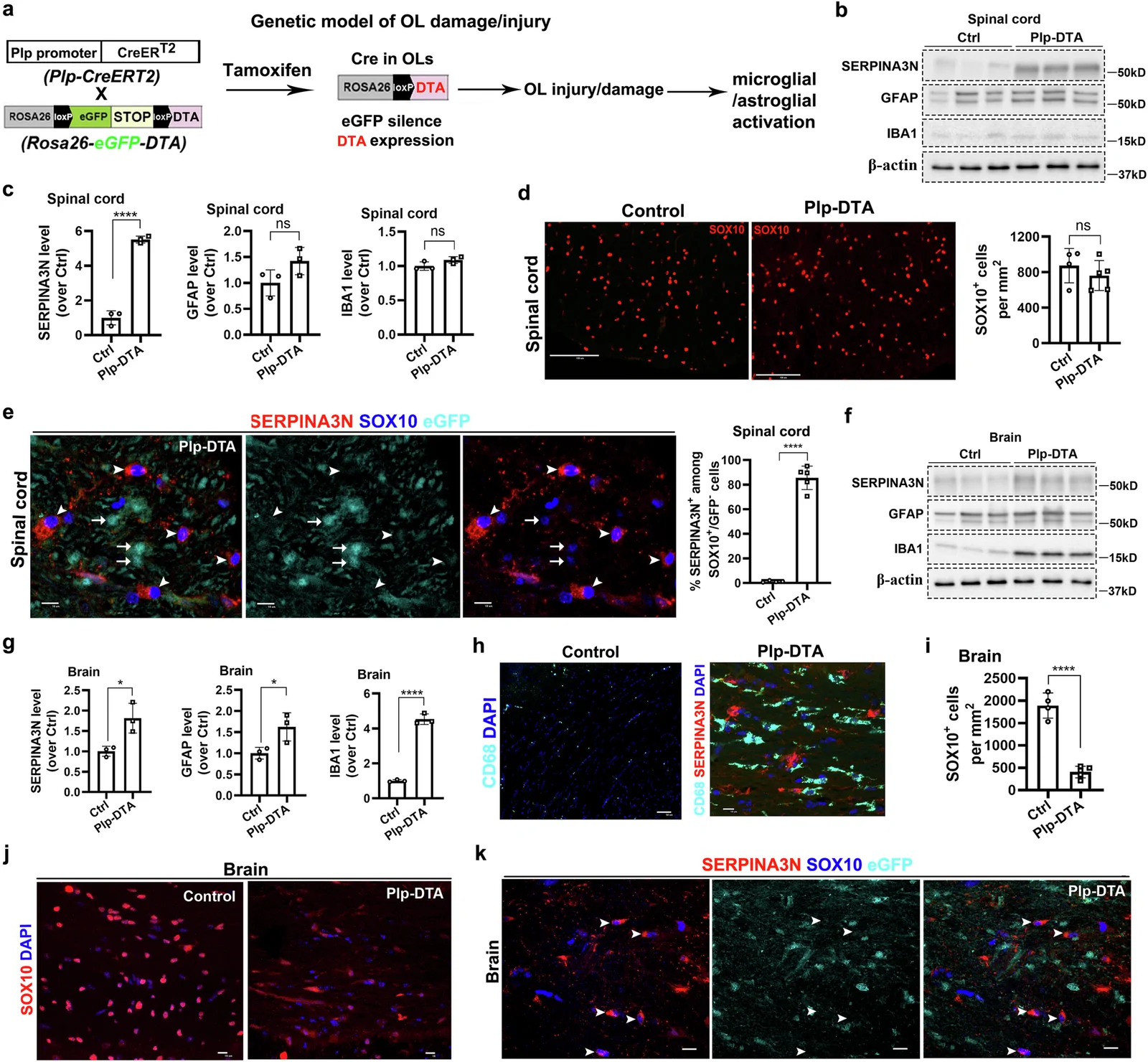
Autonomous injury transitions homeostatic OLs into SerpinOLs. **a** Diagram of the *Cre/loxP*-based genetic model of autonomous injury via diphtheria toxin fragment A (DTA) expression in *Plp*^+^ OLs. **b**, **c** Western blot and quantification of SERPINA3N and glial markers (GFAP and IBA1) in the spinal cord. ****P < 0.0001 (SERPINA3N); *P* = 0.1150 (GFAP); *P* = 0.1250 (IBA1). *n* = 3 mice per group. **d** IHC and quantification of SOX10^+^ oligodendroglial lineage cells in the ventral white matter of the spinal cord. *P* = 0.3851. *n* = 4 mice for Ctrl; *n* = 5 mice for Plp-DTA. **e** Triple IHC and quantification of SERPINA3N, SOX10, and eGFP in the ventral white matter of the spinal cord. Arrowheads indicate eGFP^-^SOX10^+^ injured OLs positive for SERPINA3N; arrows indicate eGFP^+^SOX10^+^ intact OLs negative for SERPINA3N. ****P < 0.0001. *n* = 4 mice for Control; *n* = 5 mice for Plp-DTA. **f**, **g** Western blot and quantification of SERPINA3N and glial markers (GFAP and IBA1) in the brain. *P = 0.0217 SERPINA3N; *P = 0.0404 GFAP; ****P < 0.0001 IBA1. *n* = 3 mice per group. **h** IHC images of CD68 and SERPINA3N in the corpus callosum. Quantification (**i**) and representative confocal images (**j**) of SOX10^+^ oligodendroglial cells in the corpus callosum. ****P < 0.0001. *n* = 4 mice for Control; *n* = 5 mice for Plp-DTA. **k** IHC images showing DTA-expressing SOX10^+^eGFP^-^ injured OLs positive for SERPINA3N in the corpus callosum. Arrowheads indicate DTA-injured SerpinOLs (SERPINA3N^+^SOX10^+^eGFP^-^). Representative images are shown from *n* = 5 Plp-DTA mice with similar results. Data in (**c**–**k**) were collected from the spinal cord or brain 24 h after a single i.p. injection of tamoxifen (100 mg/kg) in adult *Plp-CreER*^T2^:*Rosa26-eGFP-DTA* (Plp-DTA) mice or *Plp-CreER*^T2^ Ctrl mice. Scale bars: **d** 100 µm, **e**, **j**, **k** 10 µm, **h** 50 µm in Ctrl, 10 µm in Plp-DTA. Data are presented as mean values ± SEM. Statistical significance was determined using a two-tailed unpaired Student’s *t*-test. Source data are provided as a Source Data file.

To further elucidate the mechanism underlying SerpinOL transition, we employed a genetic model of cell-autonomous OL injury. Expression of diphtheria toxin fragment A (DTA), which disrupts eukaryotic translation and induces cellular stress and death^38^, was selectively induced in OLs using *Plp-CreER*^*T2*^*:Rosa26-eGFP-DTA* double transgenic mice (Plp-DTA). Tamoxifen administration activates DTA expression and silences eGFP specifically in *Plp*^+^ OLs, leading to OL-intrinsic injury and subsequent secondary glial activation (Fig. 6a).

In the spinal cord of Plp-DTA mice, SERPINA3N expression increased >5-fold within 24 h post-tamoxifen, while astroglial (GFAP) and microglial (IBA1) markers remained unchanged compared with Ctrl mice (Fig. 6b, c). Although the total number of OLs was unchanged (Fig. 6d), many Cre-recombined OLs (SOX10^+^eGFP^-^) showed robust SERPINA3N expression (Fig. 6e, arrowheads), while neighboring intact OLs (SOX10^+^eGFP^+^) remained SERPINA3N negative (i.e., non-SerpinOLs) (Fig. 6e, arrows). Our quantification revealed that greater than 80% of DTA-expressing SOX10^+^eGFP^-^ OLs had transitioned into SerpinOLs (Fig. 6e, right). These results demonstrate that DTA-mediated cell-autonomous OL injury is sufficient to drive the transition of homeostatic OLs into SerpinOLs.

This conclusion was further corroborated by the findings from the brain of Plp-DTA mice, where SERPINA3N levels were also significantly upregulated 24 h post-tamoxifen (Fig. 6f, g). Unlike the spinal cord, OL loss was evident in the corpus callosum (Fig. 6i, j), accompanied by strong activation of microglia and astrocytes (Fig. 6f–h), likely reflecting region-specific differences in susceptibility and kinetics of oligodendroglia to DTA-mediated damage/injury. Importantly, >90% of the surviving oligodendroglia (eGFP^-^SOX10^+^) in the brain expressed SERPINA3N (Fig. 6k, arrowheads), reinforcing the concept that intrinsic injury or damage is a primary driver for the transition of homeostatic OLs into SerpinOLs.

In contrast, no SerpinOLs were observed in *Adlh1l1-CreER*^*T2*^*:Rosa26-eGFP-DTA* double transgenic mice (Aldh1l1-DTA) (Supplementary Fig. 11a) in which tamoxifen-induced DTA expression is restricted to astrocytes (Aldh1l1^+^). Astrocyte-specific injury did not result in oligodendroglial loss (Supplementary Fig. 11b, c) or SerpinOL induction in the brain (Supplementary Fig. 11d) and spinal cord (Supplementary Fig. 11e) at 24 h post-tamoxifen. These findings indicate that astrocytic alterations play a minimal role in driving OL state transitions.

Collectively, our findings from these genetic models prove that oligodendroglial injury, rather than neuroinflammation or glial activation, is the mechanism underlying the transition of homeostatic OLs into SerpinOLs.

### SerpinOLs exhibit inflammation/immune-regulatory signatures and activation of STAT3 signaling

**Fig. 7:**
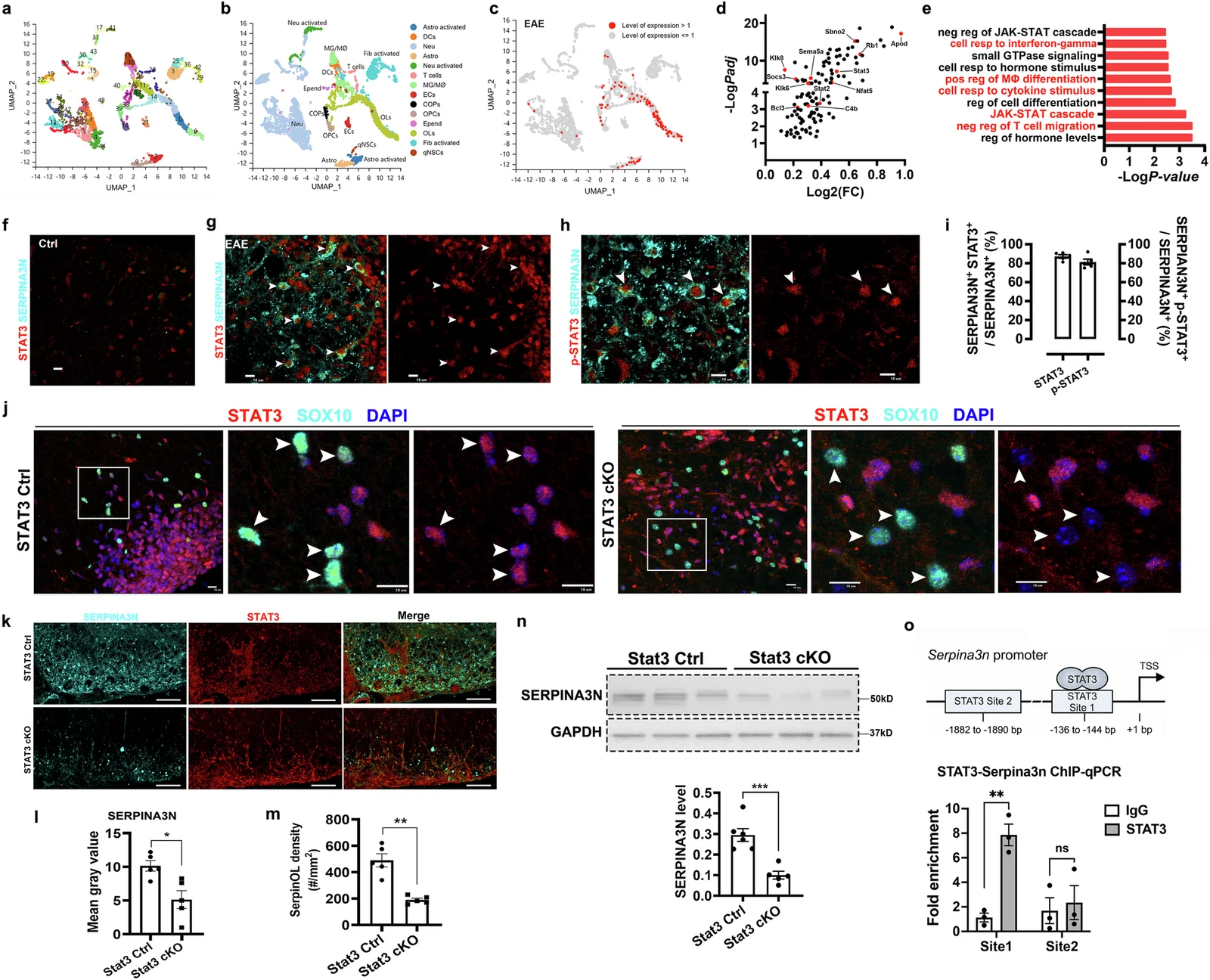
Characterization of SerpinOLs in demyelinating injury. UMAP clusters (**a**, Supplementary Data 6) and cell-type annotations (**b**) of 7946 single cells (scRNA-seq) from the spinal cord at D30 post-MOG/EAE. MG/MØ microglia/macrophages, DCs, dendritic cells, Neu neurons, Astro astrocytes, ECs endothelial cells, Epend ependymal cells, OPCs oligodendrocyte progenitor cells, COPs differentiation-committed OPCs, OLs oligodendrocytes, Fib fibroblasts, qNSC quiescent neural stem cells. **c** *Serpina3n* expression across different cell populations. *Serpina3n* mRNA is predominantly enriched in the OL clusters, with lower levels detected in astrocyte clusters. **d** Significantly upregulated genes in SerpinOLs compared with non-Serpina3n-expressing OLs, assessed by pseudobulk RNA analysis (Supplementary Data 7). **e** Examples of biological processes overrepresented by upregulated genes in SerpinOLs (Supplementary Data 7). **f** IHC of SERPINA3N and STAT3 in CFA Ctrl spinal cord. Colocalization of SERPINA3N with STAT3 (**g**, arrowheads) and active phosho-STAT3 (**h**, arrowheads) in D30 MOG/EAE spinal cord. **i** Quantification of the percentage of SERPINA3N+ cells co-expressing STAT3 (**g**) or p-STAT3 (**h**). **j** IHC showing efficient depletion of STAT3 from SOX10^+^ oligodendroglial cells in the spinal cord of *Stat3* cKO (*Olig2-Cre:Stat3*^fl/fl^) compared with *Stat3* Ctrl (*Olig2-Cre:Stat3*^+/+^) mice at D30 post-MOG/EAE. Arrowheads indicate STAT3^+^SOX10^+^ cells in *Stat3* Ctrl and STAT3^-^SOX10^+^ cells in *Stat3* cKO mice. **k**–**m** IHC and quantification of SERPINA3N in *Stat3* Ctrl and cKO spinal cord at D30 post-MOG/EAE. *P = 0.0114 (**k**), **P = 0.0004 (**m**). **n **Western blot and quantification for SERPINA3N in *Stat3* Ctrl and cKO spinal cord at D30 post-MOG/EAE. ***P = 0.0006. GAPDH was run on a separate gel using the same samples and is shown as a sample processing control. **o** Schematic of predicted STAT3 binding sites within the Serpina3n promoter (Site 1 near the TSS and distal Site 2). ChIP-qPCR showing STAT3 binding at Site 1 but not Site 2, compared with IgG control; enrichment is shown as fold change. **P = 0.0027, *P* = 0.8785. *n* = 3 independent primary OL cultures. Scale bars: **f**–**h**, **j** 10 µm; **k** 50 µm. Data are presented as mean values ± SEM. Statistical significance was determined using a two-tailed unpaired Student’s *t*-test. Source data are provided as a Source Data file.

The molecular signatures of SerpinOLs and underlying molecular mechanisms of SerpinOL transition remain incompletely understood. To investigate these, we performed single-cell RNA sequencing (scRNA-seq) on spinal cord cells from MOG/EAE mice to characterize SerpinOLs under demyelinating conditions. From 7,946 high-quality live cells pooled from three spinal cords, we identified 49 distinct clusters (C0-C48) based on cluster-specific marker genes (Fig. 7a, Supplementary Data 6). Seven clusters (C6, C0, C2, C14, C33, C39, and C18) were annotated as oligodendroglial lineage cells (Fig. 7b, Supplementary Fig. 12). Consistent with histological findings, *Serpina3n* was predominantly expressed in oligodendrocyte clusters (C6, C0, C2, C14, C33) (Fig. 7c). We defined SerpinOLs and non-SerpinOLs based on their Serpina3n expression and conducted pseudobulk differential expression analysis. This revealed 126 DEGs in SerpinOLs compared with non-SerpinOLs under MOG/EAE conditions (Fig. 7d, Supplementary Data 7). Functional annotation of these DEGs showed significant enrichment for pathways related to immune response, immune regulation, and JAK-STAT signaling (Fig. 7e, Supplementary Data 7), a pathway critically involved in inflammation modulation^39^. Notable DEGs included those with established roles in immune regulation and activation such as *Apod*^40^, *Rb1*^41^, *Nfat5*^42^, *Klk6*^43^, and *Sbno2*^44^ (Fig. 7d). While prior studies have shown that OL lineage cells acquire immune cell-like phenotypes including MHC-II molecule expression in MOG/EAE^45^, the vast majority of SerpinOLs did not express immune cell markers such as CD45 (Supplementary Fig. 13a–c), nor key MHC-II molecules such as I-A/I-E (Supplementary Fig. 13d–f) and CD74 (Supplementary Fig. 13g, h). These findings suggest that SerpinOLs are molecularly distinct from previously reported oligodendroglial populations of antigen phagocytosing and presenting properties^45^ and that SerpinOLs may exert immune-regulatory functions through MHC-II-independent mechanisms.

**Fig. 8:**
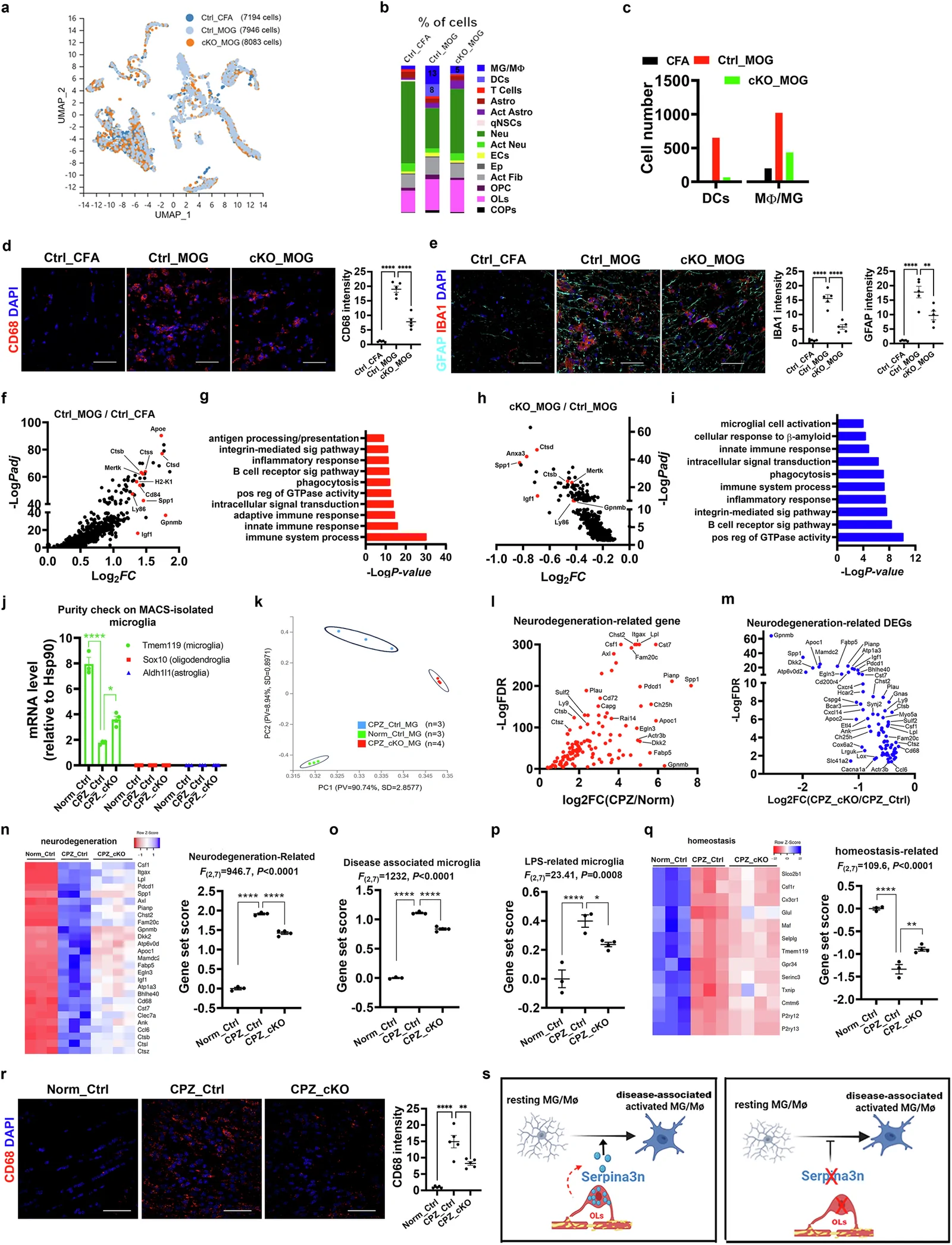
SerpinOLs regulate CNS inflammation and microglial activation in response to demyelination. **a** UMAP clusters of single cells from the spinal cord of three groups of mice at D30 post-MOG/EAE (or CFA). **b** Percentage of cell types. **c** Numbers of dendritic cells (DCs) and microglia/macrophages (MG/MØ). **d**, **e** Representative IHC images and quantifications of CD68, GFAP, and IBA1 fluorescence intensity in spinal cord white matter. ****P < 0.0001, **P = 0.0038, *n* = 5 mice per group. **f** Upregulated DEGs in Ctrl_MOG vs. Ctrl_CFA revealed by psuedobulk analysis of MG/MΦ (C11, C13, C38) (Supplementary Fig. 15, Supplementary Data 8). **g** Biological processes of upregulated DEGs in Ctrl_MOG mice (Supplementary Data 8). **h** Downregulated DEGs in cKO_MOG versus Ctrl_MOG mice revealed by pseudobulk analysis of MG/MΦ (Supplementary Fig. 15, Supplementary Data 9). **i** Biological processes of downregulated DEGs in Ctrl_MOG mice (Supplementary Data 9). **j** RT-qPCR purity check of microglia (MG) purified from Norm_Ctrl, CPZ_Ctrl and CPZ_cKO brains. ****P < 0.0001, *P = 0.0104. Microglial RNA was used for RNA-seq and bioinformatics in (**k**–**q**). **k** Principal component analysis of MG samples. **l** Upregulation of core neurodegeneration-related genes^46^ in MG of CPZ_Ctrl versus Norm_Ctrl mice (Supplementary Data 10). **m** Downregulation of core neurodegeneration-related genes^46^ in MG of CPZ_cKO versus CPZ_Ctrl mice (Supplementary Data 10). **n** Heatmap and gene set scores for neurodegeneration (Supplementary Data 10). ****P < 0.0001. Gene set scores of disease-associated (**o**)^70^ and LPS-related (**p**)^46^ microglia signatures (Supplementary Data 11). ****P < 0.0001, ***P = 0.0006, *P = 0.0493. **q** Heatmap and gene set scores of homeostatic microglia (Supplementary Data 11). ****P < 0.0001, **P = 0.0034. *n* = 3 mice for the Norm_Ctrl and CPZ_Ctrl groups; *n* = 4 mice for the CPZ_cKO group (**j**–**q**). **r** IHC and quantification of CD68 fluorescence intensity. ****P < 0.0001, **P = 0.0029. *n* = 5 mice per group. **s** Graphic conclusion. Oligodendrocyte-derived SERPINA3N promotes the activation of homeostatic microglia toward disease-associated and neurodegeneration-related states. Created in BioRender. https://BioRender.com/k5ria9m. Scale bars: **h**, **i**, **r** 50 µm. Data are mean ± SEM. Statistical significance was determined using one-way ANOVA followed by Tukey’s multiple comparisons test. Source data are provided as a Source Data file.

Importantly, we found that JAK/STAT signaling and target genes (*Stat3, Stat2, Socs3, Bcl3, and Sbno*) were significantly enriched in SerpinOLs (Fig. 7d, e). Immunostaining revealed that while STAT3 is broadly expressed in the spinal cord of CFA control mice (Fig. 7f), it is strongly induced following MOG/EAE (Fig. 7g, h). Notably, quantitative analysis in MOG/EAE spinal cords revealed that approximately 87.7% of SerpinOLs were positive for STAT3 (Figs. 7g, i) and 81.3% expressed its active phosphorylated form, p-STAT3 (Fig. 7h, i). These high co-expression rates suggest a strong link between STAT3 activation and SerpinOL state. To directly test the role of STAT3 signaling in SerpinOL transition, we generated oligodendroglial-specific *Stat3* cKO mice (*Olig2-Cre:Stat3*^fl/fl^). Deletion of *Stat3* in OLs (Fig. 7j) resulted in a marked reduction in SERPINA3N expression (Fig. 7l, n) and a significant decrease in the density of SerpinOLs (Fig. 7m). Mechanistically, promoter sequence analysis identified two putative STAT3 binding motifs at −135 to −144 bp (Site 1) and −1882 to −1890 bp (Site 2) relative to the transcription start site (TSS) of *Serpina3n*. ChIP-qPCR analysis in H_2_O_2_-injured primary oligodendrocytes^27^ revealed significant STAT3 enrichment at Site 1 compared with IgG controls (Fig. 7o), indicating that STAT3 directly activates *Serpina3n*. Furthermore, SERPINA3N depletion attenuated CNS inflammation (see Fig. 8 in the next section) in demyelinating animals and was associated with reduced STAT3 in oligodendroglial cells (Fig. S12). Together, these findings suggest a reciprocal regulation between STAT3 and SERPINA3N in oligodendroglial lineage cells.

### SerpinOLs regulate CNS inflammation and glial activation during demyelination

To determine the functional relevance of OL-derived SERPINA3N in diseased conditions, we generated transgenic mice with oligodendroglia-specific SERPINA3N deficiency and subjected them to MOG/EAE demyelinating injury. scRNA-seq analysis (Fig. 8a) revealed a significant increase in both the proportion (Fig. 8b) and the number (Fig. 8c) of myeloid cells, including DCs (C10) and microglia/macrophages (MG/MØ; C11, C13, and C38) (Supplementary Fig. 15a–c), in the spinal cords of MOG versus CFA Ctrl mice, which is consistent with the known role of myeloid cell-mediated CNS inflammation in MOG/EAE demyelination^21^. Strikingly, SERPINA3N ablation significantly reduced myeloid cell populations in the spinal cords of cKO_MOG mice compared with Ctrl_MOG mice (Fig. 8b, c). Histological analysis confirmed a significant reduction in the density of CD68^+^ cells in the spinal cord white matter of cKO_MOG mice (Fig. 8d). Furthermore, the extent of gliosis was attenuated, as evidenced by decreased densities of IBA1^+^ microglia/macrophages and GFAP^+^ astroglia in cKO_MOG mice (Fig. 8e). These findings were congruent with scRNA-seq results showing decreased expression of CD68 (Supplementary Fig. 15d) and suggest that SerpinOLs regulate CNS inflammatory response to MOG/EAE injury through producing SERPINA3N. To further explore this mechanism, we conducted pseudobulk RNA-seq analysis of MG/MØ clusters (C11, C13, C38). In Ctrl_MOG mice, 947 genes were significantly upregulated compared with Ctrl_CFA mice (Fig. 8f, Supplementary Data 8); these genes are primarily related to inflammation, immune response, and phagocytosis (Fig. 8g). Interestingly, of the 947 DEGs, 417 (44%) were significantly downregulated in MG/MØ of cKO_MOG versus Ctrl_MOG mice (Fig. 8h), representing 79% of the 530 total downregulated DEGs (417/530) in cKO_MOG conditions (Supplementary Data 9). These 417 genes were primarily associated with inflammatory/immune responses and phagocytosis among others (Fig. 8i), indicating that SERPINA3N deficiency dampens pro-inflammatory transcriptomic profiles of MG/MØ in MOG/EAE. Given that microglia/macrophages do not express SERPINA3N in MOG/EAE, our findings suggest that SERPINA3N secreted from SerpinOLs acts in a paracrine manner to promote MG/MØ-driven neuroinflammation during EAE injury. Importantly, the reduction in neuroinflammation translated into significant functional and tissue protection, as we found that Serpina3n-deficient mice exhibited substantially improved motor symptoms (clinical scores) (Supplementary Fig. 16a–c) and attenuated spinal cord myelin damage (Supplementary Fig. 16d) in MOG/EAE compared with *Serpina3n*-sufficient MOG/EAE mice.

To further validate the role of SerpinOLs in neuroinflammation and microglial activation, we employed CPZ demyelination model in which microglia, but not monocyte-derived macrophages, drive neuroinflammation (cf Supplementary Fig. 3a). RNA-seq was performed on microglia purified from brains of healthy controls (Norm_Ctrl), CPZ-treated controls (CPZ_Ctrl), and CPZ-treated *Serpina3n* cKO (CPZ_cKO) mice (Fig. 8j). PCA analysis showed that SERPINA3N deficiency markedly shifted microglial transcriptomic profiles away from those of CPZ_Ctrl group (Fig. 8k), which is indicative of an altered activation state.

A microglial gene module comprising 134 core neurodegeneration-related genes^46^ was used to assess disease-related microglial activity. Of these, 125 (93%, 125/134) were upregulated in microglia of CPZ_Ctrl versus Norm_Ctrl groups (Fig. 8l, Supplementary Data 10), which is consistent with the finding that activated microglia are essential for CPZ-mediated demyelination^47^. Remarkably, 81 of these 125 genes (64.8%) were significantly downregulated in CPZ_cKO versus CPZ_Ctrl mice (Fig. 8m, Supplementary Data 10), suggesting that SERPINA3N deficiency attenuates neurodegeneration-associated microglial activation. To further characterize microglial state, we calculated cumulative gene set scores (Supplementary Data 11**;** see “Methods” section). Gene sets associated with neurodegeneration (Fig. 8n), disease-associated microglia (Fig. 8o), and LPS-induced pro-inflammatory activation (Fig. 8p) were all significantly decreased in CPZ_cKO versus CPZ_Ctrl microglia. In contrast, the homeostasis-related gene set score was significantly increased in CPZ_cKO microglia (Fig. 8q). Histological analysis further confirmed a significant reduction in the density of CD68^+^ cells in the corpus callosum of CPZ_cKO mice (Fig. 8r). These data support a working model in which SERPINA3N depletion in SerpinOLs shifts microglia from a disease-associated and pro-inflammatory state toward a more homeostatic state in response to demyelination (Fig. 8s). Consistent with the reduction in neuroinflammation and microglial activation, SERPINA3N deficiency conferred robust protection against white matter injury in CPZ demyelination model, as we found a significant decrease in CPZ-elicited oligodendrocyte loss (Supplementary Fig. 16e) and myelin damage (Supplementary Fig. 16f). Moreover, SERPINA3N deficiency promoted tissue repair by enhancing the generation of newly formed OLs and accelerating remyelination (Supplementary Fig. 17). Collectively, these functional data establish that injury-transduced SerpinOLs aggravate CNS inflammation, promote microglial activation toward a disease-associated and neurodegeneration-related activation state, and exacerbate oligodendrocyte/myelin damage.

### SerpinOLs regulate neuroinflammation and glial activation in non-demyelinating conditions

**Fig. 9:**
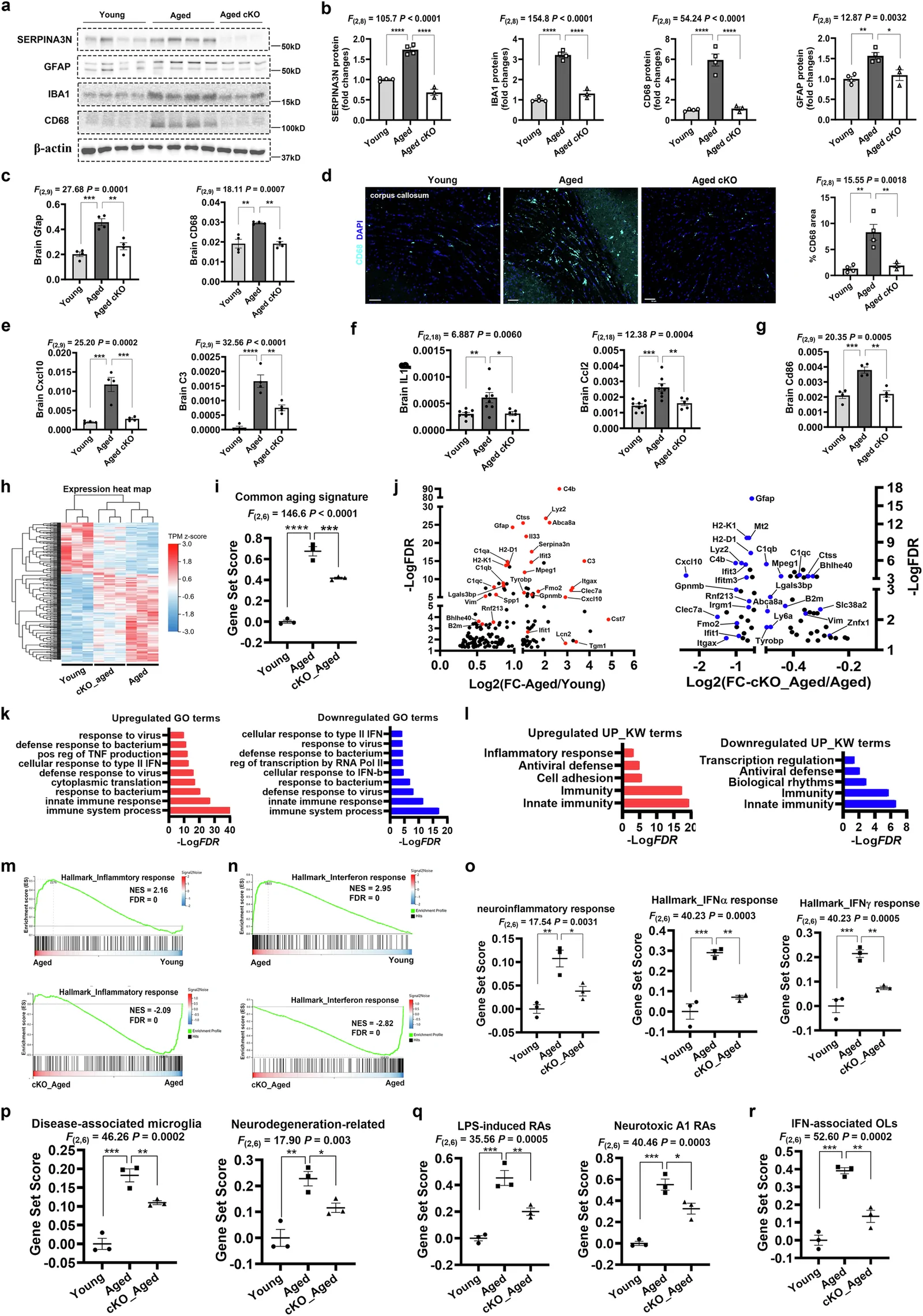
SerpinOLs regulate CNS inflammation and glial responses during normal aging. **a**, **b** Western blot and quantification of SERPINA3N, IBA1, CD68, and GFAP. ****P < 0.0001, **P = 0.0035, *P = 0.0147. β-actin is a sample processing control. **c** RT-qPCR of brain *Gfap* and *Cd68*. *Gfap* ***P = 0.001, **P = 0.0012; *Cd68* **P = 0.0014. **d** IHC and quantification of CD68. Scale bar = 50 µm. **P = 0.0023, Aged vs Young; **P = 0.0063, cKO_Aged vs Aged. *n* = 4 mice, Young and Aged; *n* = 3 mice, Aged cKO (**a**, **b**, **d**). **e** RT-qPCR of *Cxcl10* and *C3*. *Cxcl10*, ***P = 0.0003 Aged vs Young, ***P = 0.0006 cKO_Aged vs Aged; *C3*, ****P < 0.0001, **P = 0.0033. **f** RT-qPCR of *IL-1b* and *Ccl2*. *IL-1b* **P = 0.0092, *P = 0.0257, *Ccl2* ***P = 0.0005, **P = 0.006. *n* = 8 mice for Young and Aged groups; *n* = 5 mice for Aged cKO group. **g** RT-qPCR of *Cd86*. ***P = 0.0008, **P = 0.0012. **c**, **e**, **g** *n* = 4 mice per group. **h** Unsupervised clustering heatmap of DEGs (Supplementary Data 12). **i** Gene set scores of common aging signatures^32^ (Supplementary Data 11). ****P < 0.0001, ***P = 0.0006. **j** DEGs upregulated in Aged versus Young and downregulated in cKO_Aged versus Aged. GO (**k**) and UniProt keyword (UP_KW_BP, **l**) analysis. GSEA of MSigDB Hallmark Pathways showing upregulation of inflammatory response (**m**) and interferon response (**n**) in aged brain versus young (upper panels) and downregulation in cKO_Aged brain versus aged (lower panels). NES, normalized enrichment score. **o**–**r** Gene set scores. **o** Neuroinflammatory response (**P = 0.0033, *P = 0.0183), IFNα response (***P = 0.0004, **P = 0.0013), and IFNγ response (***P = 0.0005, **P = 0.0034). **p** Disease-associated microglia (DAM, ***P = 0.0002, **P = 0.0088) and neurodegeneration-related microglia (NDM, **P = 0.0022, *P = 0.0451). **q** LPS-induced reactive astrocytes^28^ (***P = 0.0005, **P = 0.0066) and neurotoxic A1 reactive astrocytes^71^ (***P = 0.0003, *P = 0.0105) (Supplementary Data 11). **r** Interferon-responsive OLs. ***P = 0.0002, **P = 0.0011. **h**–**r** *n* = 3 mice per group. Data are mean values ± SEM. Statistical significance was determined using one-way ANOVA followed by Tukey’s multiple comparisons test. Source data are provided as a Source Data file.

We next investigated whether the function of SerpinOLs in regulating neuroinflammation is preserved in non-demyelinating conditions. Given our findings that homeostatic OLs transition into SerpinOLs during normal aging (Fig. 3), we hypothesize that SerpinOLs contribute to age-related neuroinflammation and glial activation. To test this hypothesis, we examined the brains of young (2 mon), aged (20 mon), and aged *Serpina3n* cKO (20 mon, *Olig2-Cre:Serpina3n*^fl/fl^) mice. SERPINA3N deletion was confirmed by Western blot (Fig. 9a, b) and histological (cf Fig. 3c, e) assays. Notably, depleting SERPINA3N attenuated aging-elicited activation of microglia and astrocytes, as evidenced by reduced expression of CD68, IBA1 and GFAP at both mRNA and protein levels in aged cKO brains compared with age-matched Ctrl mice (Fig. 9a–c). Histological quantification confirmed that SERPINA3N deletion normalized CD68^+^ area in the corpus callosum of aged cKO mice to levels observed in young mice (Fig. 9d). Aging is known to drive astrocyte activation toward the neurotoxic A1 phenotype^30^; this was supported by increased expression of the representative pan-reactive astrocyte marker *Cxcl10* and neurotoxic reactive astrocyte marker *C3* (Fig. 9e). SERPINA3N deletion significantly reduced the expression of these markers in aged cKO brain (Fig. 9e). Additionally, expression of aging-elicited pro-inflammatory cytokine *IL-1b* and chemokine *Ccl2* was significantly decreased in aged cKO mice (Fig. 9f), alongside reduced expression of M1-polarized pro-inflammatory microglial marker CD86 (Fig. 9g). These results suggest that OL-derived SERPINA3N promotes aging-associated neuroinflammation and glial activation toward pro-inflammatory and neurotoxic states.

We next performed unbiased brain RNA-seq to strengthen our conclusions. Unsupervised heatmap clustering of DEGs (Supplementary Data 12) revealed distinct separation between aged cKO and aged Ctrl mice (Fig. 9h), suggesting that SERPINA3N deletion alters aged brain transcriptomics. We examined the expression of a recently identified common aging signature (CAS) consisting of 82 differentially regulated genes in the murine brain^32^ and found that SERPINA3N deletion significantly reversed this common aging signature (Fig. 9I). Specifically, most DEGs induced by normal aging (Fig. 9j, red) were significantly downregulated in *Serpina3n* cKO mice (Fig. 9j, blue), including disease-associated/neurodegeneration-related microglial signature genes (*Spp1, Ctss, Itgax, Clec7a, Gpnmb, Bhlhe40*, *Lyz2, Tyrobp, and Cts7*), complement genes (*C1qa, C1qb, C1qc, C4b*, and *C3*), pan-reactive astrocyte marker genes (*Lcn2, Gfap*, and *Vim*), and A1 neurotoxic reactive astrocyte marker genes (*C3, B2m, Ifit3, Ifit1, H2-D1*, and *H2-K1*) (Fig. 9j). GO analysis revealed that normal aging activated innate immune response and inflammatory response (Fig. 9k, l, red) whereas SERPINA3N deficiency significantly reduced these pathways in aged cKO brain (Fig. 9k, l, blue). These findings indicate that SERPINA3N accelerates brain aging through promoting CNS inflammation and glial activation.

To further corroborate our findings, we performed gene set enrichment analysis (GSEA) using the MSigDB molecular signature database. Our results showed that SERPINA3N depletion reversed brain inflammatory response (Fig. 9m) and interferon response (Fig. 9n) in *Serpina3n* cKO mice compared with aged Ctrl mice, which was confirmed by significantly rescued gene set scores of neuroinflammatory and interferon type I (IFNα) and type II (IFNγ) responses (Fig. 9o). These data suggest that SERPINA3N promotes aging-associated neuroinflammation and cellular responses to interferons, crucial cytokines for innate immune activation.

Microglial and astroglial activation are key mediators of age-related neuroinflammation^48^. We found that SERPINA3N deficiency reduced microglial cell activation (Supplementary Fig. 18a) and significantly decreased the activation states of disease-associated and neurodegeneration-related microglia (Fig. 9p), as well as LPS-induced pro-inflammatory response (Supplementary Fig. 18b), and interferon-related response (Supplementary Fig. 18c) with minimal effect on microglial proliferation (Supplementary Fig. 18d) and homeostasis (Supplementary Fig. 18e). Similarly, SERPINA3N deletion mitigated aging-elicited astrocyte reactivity, including LPS-induced pro-inflammatory response^28^ and A1 neurotoxic phenotypes^30^ (Fig. 9q). These findings indicate that SerpinOL-derived SERPINA3N is a key regulator of aging-elicited glial activation.

Previous studies have reported that OLs are activated into different disease-associated subpopulations with distinct transcriptomic signatures, including DA1 OLs (immunogenic), DA2 OLs (survival/differentiation) and interferon-responsive OLs^11,15^. We found that normal aging predominantly induced an IFN-responsive OL state (Fig. 9r) in the brain, with no significant enrichment of DA1 or DA2 signatures (Supplementary Fig. 18f, g). Importantly, depleting SERPINA3N significantly reduced the activation state of interferon-responsive OLs (Fig. 9r), suggesting that SERPINA3N modulates oligodendroglial responses to interferon during normal aging^15^.

In line with attenuated neuroinflammation and reduced glial activation, we found that SERPINA3N deficiency exerted protective effects on brain damage, as evidenced by significant rescue in aging-elicited axonal dysfunction (Supplementary Fig. 19a), myelin damage (Supplementary Fig. 19a–d), and oxidative stress injury (Supplementary Fig. 19e). Taken together, our functional data demonstrate that SerpinOLs, acting through SERPINA3N production, regulate neuroinflammation and promote glial activation toward neurodegenerative and neurotoxic states that exacerbate brain injury during normal aging.

### SERPINA3N promotes microglial activation toward pro-inflammatory states

**Fig. 10:**
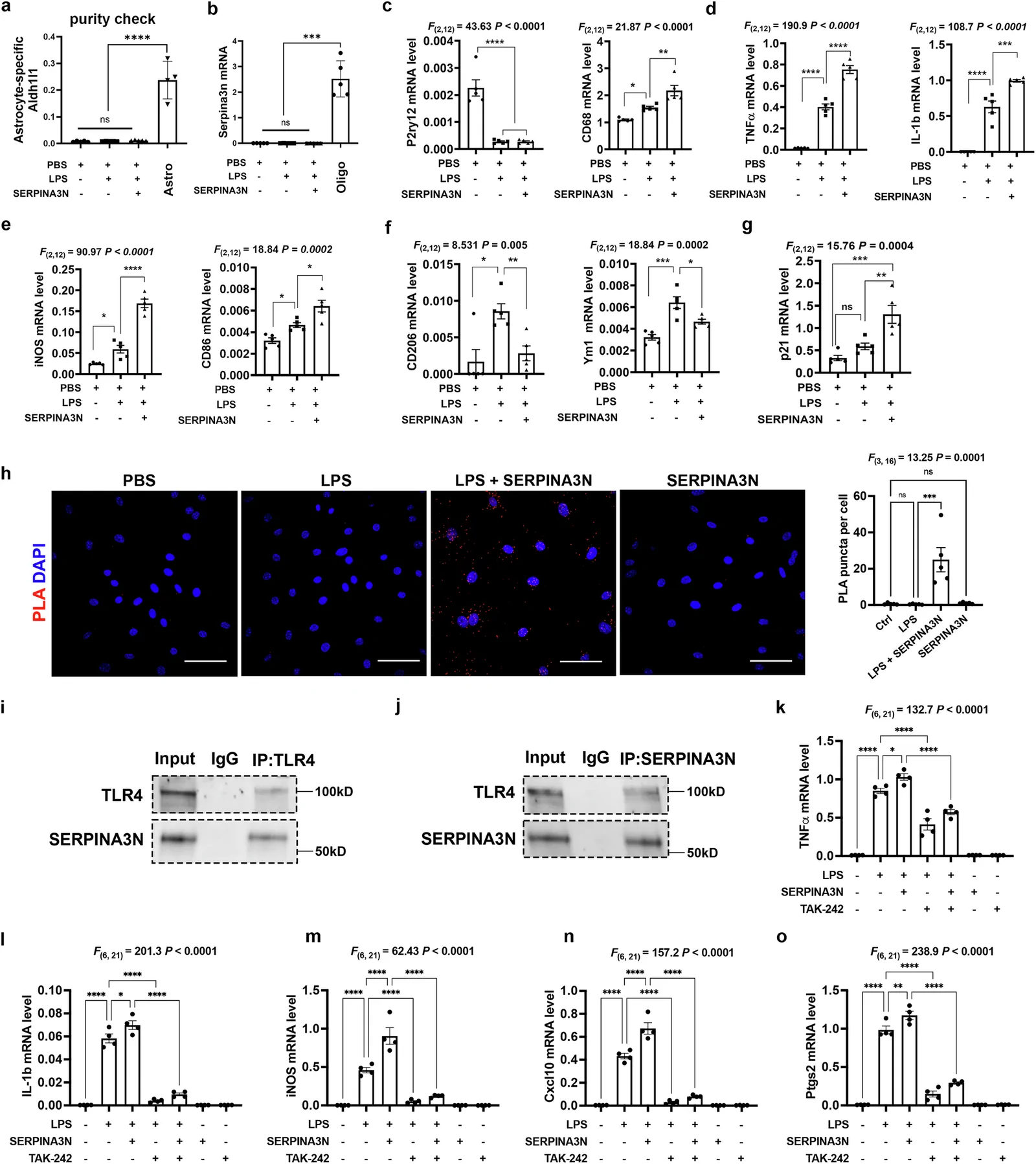
SERPINA3N promotes activated microglia toward a pro-inflammatory state. Primary microglia were treated with LPS (10 ng/mL), recombinant mouse SERPINA3N (50 ng/mL) and/or TAK-242 for 24 h before RNA isolation. **a** RT-qPCR purity assessment using astrocytic marker *Aldh1l1*. Primary astrocytes (Astro) were used as a positive control. ****P < 0.0001. **b** RT-qPCR showing absence of *Serpina3n* mRNA in cultured microglia. Primary oligodendrocytes (Oligo) were used as a positive control. ***P < 0.001. **c** RT-qPCR of *P2ry12* and *Cd68*. *P2ry12* ****P < 0.0001; *Cd68* *P = 0.046 LPS vs PBS, *P = 0.0059 LPS vs LPS + SERPINA3N. **d** RT-qPCR of *Tnfα* and *IL-1b*. *Tnfα* ****P < 0.0001; *IL-1b* ***P = 0.0005, ****P < 0.0001. RT-qPCR of pro-inflammatory microglial markers *iNos* and *Cd86* (**e**) and anti-inflammatory microglial markers *Cd206* and *Ym1* (**f**). *iNos* *P = 0.0228, ****P < 0.0001; *Cd86* *P = 0.0428 LPS vs PBS, *P = 0.0142 LPS vs LPS + SERPINA3N; *Cd206* *P = 0.0188, **P = 0.0060; *Ym1* ***P = 0.0001, *P = 0.0142. **g** RT-qPCR for the cell senescence marker *p21* (*Cdkn1a*). ns *P* = 0.3568, **P = 0.0047, ***P = 0.0004. *n* = 5 independent biological replicates (**a**–**g**). **h** In situ PLA in primary microglia treated with PBS, LPS, LPS + SERPINA3N, or SERPINA3N alone, with quantification of PLA puncta per cell. ns *P* > 0.9999 Ctrl vs LPS; ns, *P* = 0.9998 Ctrl vs SERPINA3N; ***P = 0.0005. *n* = 5 independent microglial cultures. Scale bars: 50 µm. Co-IP of TLR4 and SERPINA3N interaction in primary microglia treated with LPS and SERPINA3N protein, using anti-TLR4 (**i**) or anti-SERPINA3N (**j**) antibodies. Input lysates and isotype IgG immunoprecipitants were included as controls. Representative Western blots from three independent experiments with similar results are shown. RT-qPCR of *Tnfα* (**k**), *IL-1b* (**l**), *iNos* (**m**), *Cxcl10* (**n**), and *Ptgs2* (**o**) in primary microglia treated with LPS, SERPINA3N, and/or TAK-242. *Tnfα* *P = 0.031, ****P < 0.0001; *IL-1b* *P = 0.0137, ****P < 0.0001; *iNos* and *Cxcl10* ****P < 0.0001; *Ptgs2* **P = 0.0062, ****P < 0.0001. *n* = 4 independent biological replicates. Data are mean values ± SEM. Statistical significance was determined using one-way ANOVA followed by Tukey’s multiple comparisons test. Source data are provided as a Source Data file.

To determine whether SERPINA3N directly promotes pro-inflammatory microglial activation, we utilized primary microglial cultures and a SERPINA3N gain-of-function approach. RT-qPCR assay confirmed the isolated primary microglia were free of astrocyte contamination (Fig. 10a) and did not express SERPINA3N under unstimulated (PBS), LPS-stimulated, and SERPINA3N-treated conditions (Fig. 10b). As expected, LPS stimulation significantly upregulated *Cd68* while reducing *P2ry12*, a marker of homeostatic microglia (Fig. 10c). We found that treatment with recombinant SERPINA3N further enhanced *Cd68* expression (Fig. 10c), suggesting that SERPINA3N potentiates microglial activation. In addition, SERPINA3N significantly upregulated pro-inflammatory cytokines *Tnfα* and *IL-1b* (Fig. 10d), as well as pro-inflammatory markers *iNos* and *Cd86* (Fig. 10e). In contrast, it suppressed the expression of anti-inflammatory markers *Cd206* and *Ym1* (Fig. 10f), consistent with a shift toward a pro-inflammatory phenotype. Microglial senescence is increasingly recognized as a pathological feature of the aged brain^49^. We found that SERPINA3N treatment markedly increased the expression of *p21* (*Cdkn1a*), a well-established marker of cell senescence^50^ in LPS-stimulated microglia. These findings demonstrate a direct effect of SERPINA3N on microglial activation.

To gain mechanistic insight into how SERPINA3N promotes microglial activation toward a pro-inflammatory state, we examined whether SERPINA3N directly engages Toll-like receptor 4 (TLR4), a primary and potent cell-surface receptor that mediates pro-inflammatory signaling in microglia. Because microglia themselves do not express detectable SERPINA3N, recombinant SERPINA3N provided a reductionist approach to test whether oligodendrocyte-derived SERPINA3N can act directly on microglia. Using this system, we found that recombinant SERPINA3N physically interacted with TLR4, as demonstrated by proximity ligation assay (PLA, Fig. 10h) and co-immunoprecipitation with anti-TLR4 (Fig. 10i) and anti-SERPINA3N (Fig. 10j) antibodies. We next asked whether this interaction has functional consequences for microglial pro-inflammatory activation. In primary microglia stimulated with LPS, SERPINA3N further potentiated the expression of multiple TLR4-responsive pro-inflammatory genes, including *Tnfα* (Fig. 10k), I*L*-*1b* (Fig. 10l), *iNos* (Fig. 10m), *Cxcl10* (Fig. 10n), and *Ptgs2* (Fig. 10o). Importantly, inhibition of TLR4 signaling with TAK-242, a selective TLR4 inhibitor, markedly attenuated the SERPINA3N-dependent enhancement of these pro-inflammatory mediators (Fig. 10k–o). Thus, SERPINA3N not only binds TLR4, but also functionally amplifies TLR4-dependent pro-inflammatory signaling in microglia. Together, these findings provide a direct mechanistic link between oligodendrocyte-derived SERPINA3N and the pro-inflammatory activation state of microglia. Specifically, our data support a model in which oligodendrocyte-derived SERPINA3N acts directly on microglia and promotes pro-inflammatory activation through TLR4-dependent signaling.

## Discussion

Oligodendroglial lineage cells (OPCs and OLs) transition into distinct states identified by their gene expression profiles in response to CNS pathologies. Recently, a subpopulation of immune oligodendroglial lineage cells has been proposed in demyelinating diseases and animal models^13,51–53^. For instance, OPCs exposed to demyelination acquire phagocytic and antigen-presenting capabilities, influencing the function of CD8^+^ cytotoxic T cells^54^ and CD4^+^ helper T cells^13^ through upregulation of major histocompatibility complex class I (MHC-I)^54^ and MHC-II^13^ molecules, respectively. However, MHC-expressing oligodendroglial lineage cells are rare^45^ or absent^55^ in vivo. Moreover, the role of mature OLs, far more abundant than OPCs, in modulating CNS inflammatory and immune responses remains elusive. In this study, we found that SerpinOLs represent a substantial subpopulation of mature oligodendrocytes (40~60%) during CNS demyelination. They are characterized by gene signatures associated with immune and inflammatory responses and STAT3 signaling activation, which underlies SERPINA3N production in OLs. We provide genetic evidence that SerpinOLs perpetuate CNS immune and inflammatory responses to deleterious insults. Thus, our findings point to a potential myelination-independent function of mature OLs through SERPINA3N-regulated neuroinflammation and glial activation.

Our findings challenge the widely cited concept that Serpina3n/SERPINA3N is a reactive astrocyte marker in the diseased CNS. Our results unravel that OLs are the major cell type producing SERPINA3N protein in diverse types of CNS diseases and injuries. A decade ago, *Serpina3n* mRNA was reported as a reliable marker of reactive astrocytes in LPS endotoxic and ischemic brain injury models^28^, and more recently, it has been identified as a representative signature gene of disease-associated astrocytes in Alzheimer’s disease^29^ and normal aging^29,30^. In contrast, other studies reported that *Serpina3n* is a representative signature gene of disease-associated or specific oligodendrocytes under neurological conditions^10,11,13,15,26^. Our RNA-seq data reveal that both OLs and astrocytes exhibit comparably high levels of *Serpina3n* mRNA under demyelinating conditions (Fig. 1l). However, SERPINA3N protein is produced predominantly in OLs, as confirmed by a tdTom reporter mouse line in which tdTom expression depends on SERPINA3N protein translation (Fig. 2). These observations led us to hypothesize that *Serpina3n* mRNA is actively translated into SERPINA3N protein in OLs, but the translation is less efficient in astrocytes. Indeed, our RiboTag data (Fig. 1n) support this hypothesis, demonstrating that ribosome-bound and actively translated *Serpina3n* mRNA is increased in OLs and much higher than that in astrocytes upon CNS demyelination (Fig. 1o). Likewise, in the LPS-induced endotoxic brain model, SERPINA3N protein and tdTom are observed predominantly in OLs (Supplementary Fig. 6c–e) despite high *Serpina3n* mRNA expression in astrocytes^28^. In Alzheimer’s disease models (5xFAD), our group (Supplementary Fig. 8a, c) and others^29^ observed remarkable overlap of SERPINA3N-like signal with GFAP^+^ processes in the subiculum and dentate gyrus, leading to the interpretation that SERPINA3N is a marker of disease-associated astrocytes^29^. However, we interpreted this observation as an imaging artifact rather than true expression because the star-shaped SERPINA3N-like signal does not exist in single IHC using an anti-SERPINA3N antibody (Supplementary Fig. 8b, d). Similarly, in the aged brain, we unequivocally demonstrate that SERPINA3N protein is produced predominantly by OLs using oligodendroglial *Serpina3n* cKO mice (*Olig2-Cre:Serpina3n*^fl/fl^) (Fig. 3, Fig. 8). Thus, OLs, but not astrocytes, are the major cell type producing SERPINA3N.

The underlying mechanisms of SerpinOL transition from homeostatic OLs remain poorly defined. SerpinOLs are not specific to CNS demyelinating diseases in which OLs are the direct cellular target. Our data (Supplementary Fig. 5–9, Fig. 3) suggest that SerpinOLs may be present in any disease condition where OL injury occurs. This is supported by our results derived from the acute CPZ model (Fig. 5) and DTA-induced injury model (Fig. 6). In the CPZ model, approximately 20% of homeostatic OLs already transition to SerpinOLs after a very brief 2-day CPZ treatment when glial activation is yet to be detected. In the DTA model^56^, where OL injury is cell-autonomous and independent of inflammatory cues or disease-specific pathologies, over 80% of DTA-expressing OLs transition into SerpinOLs within 24 h post-tamoxifen when microglial/astroglial activation is yet to be detected in the spinal cord. Furthermore, our results collected from a panel of genetic mutant mice establish that neuroinflammation and pro-inflammatory mediators, which are ubiquitously present in various CNS diseases, are insufficient to drive the state transition of homeostatic OLs into SerpinOLs in the absence of OL injury. Therefore, OL injury, rather than neuroinflammation and glial activation, is a crucial mechanism underlying SerpinOL transition. In this regard, we define SerpinOLs as an injury-transduced activation state and propose their presence as a sensitive marker of OL injury.

While a growing list of disease-associated or specific oligodendroglia is anticipated, their functions are largely unknown^10^ or speculative^17^ thus far. The current study addresses these knowledge gaps. By leveraging oligodendroglial *Serpina3n* cKO mouse lines^27^, our results convincingly demonstrate that ablating SERPINA3N attenuates inflammatory responses to CNS demyelination and shifts microglial activation toward a more homeostatic state. To further extend this observation to non-diseased conditions, we used the healthy aging model. Consistently, deleting SERPINA3N remarkably reduces aging-elicited upregulation of pro-inflammatory cytokines and chemokines and shifts glial activation signatures of aged brains toward those of young brains (Fig. 9). Thus, our study suggests that SerpinOLs, a common and large population of injury-transduced mature OLs, amplify pro-inflammatory responses to CNS diseases and normal aging.

In addition to regulating ubiquitous neuroinflammation, the presence of SerpinOLs across CNS pathologies of distinct etiologies suggests that they play a critical role in regulating disease-specific pathophysiology, ranging from demyelination, endotoxicity, stroke, and CNS trauma, to neurodegeneration and normal aging. For instance, SerpinOLs may actively influence extracellular plaque pathology in Alzheimer’s disease by marking Aβ with SERPINA3N (Supplementary Fig. 7d). However, while SERPINA3N is a secretory protein dysregulated in biofluids of many neurological conditions^19^, its marked cytoplasmic accumulation in injured OLs has sparked debate regarding its functional impact. Although earlier studies proposed that SERPINA3N protects oligodendrocytes against injury^11,57,58^, our recent in vitro study suggests that it may potentiate oxidative stress and senescence^27^. Here, using oligodendrocyte-specific *Serpina3n* cKO mice, we provide definitive in vivo evidence that OL-derived SERPINA3N functions as a pathogenic amplifier rather than a cytoprotective shield. In demyelinating models, *Serpina3n* deficiency attenuated neuroinflammation, preserved myelin, and enhanced functional recovery and remyelination (Supplementary Fig. 16, 17). Furthermore, in non-diseased aging, depleting SERPINA3N mitigated glial activation, oxidative stress, and axonal and myelin degeneration (Supplementary Fig. 19). Collectively, these functional data weaken the neuroprotective hypothesis and indicate that injury-induced SERPINA3N expression in oligodendrocytes exacerbates neuroinflammation and tissue damage across diverse CNS pathologies.

We found that SERPINA3N does not influence microglial activation or phenotype polarization in the absence of LPS stimulation in vitro (Fig. 10). This observation suggests that SERPINA3N is insufficient to trigger glial activation de novo. We hypothesize that SERPINA3N exerts its biological functions, such as promoting neuroinflammation and glial activation, in the context of diseased/injured conditions but not under CNS homeostasis, as our recent study concluded^27^. This conclusion is in sharp contrast to a recent study^59^ reporting that SERPINA3N induces robust inflammatory responses in the homeostatic brain. We conjecture that the reported effect in that study^59^ may result from CNS trauma elicited by injection needles during experimentation. In this regard, homeostatic transgenic mice carrying enforced *Serpina3n*-expressing alleles will be required to prove or falsify this hypothesis. Furthermore, it would be interesting to define the role of SerpinOLs in CNS traumatic injury (Supplementary Fig. 9) using our cell-specific genetic mouse models.

The molecular mechanisms underlying SERPINA3N-regulated microglial pro-inflammatory activation remain to be established. As a member of the serpin family, SERPINA3N is best known for inhibiting the cleavage activity of endogenous serine proteases, although non-canonical functions beyond protease inhibition have also been reported^19^. In addition, SERPINA3N is a secretory glycoprotein with N-linked glycosylation, raising the possibility that it may modulate microglial activation and function through direct interaction with cell-surface receptors. Therefore, identifying SERPINA3N binding partners (serine proteases and non-protease receptors) is essential for understanding how this oligodendrocyte-derived factor regulates microglial activation^60^. In the present study, we provide evidence that TLR4 is one such binding partner, a cell-surface receptor that triggers microglial pro-inflammatory responses in various neurological diseases and injury. Using PLA and co-immunoprecipitation, we found that SERPINA3N physically associates with TLR4 in primary microglia (Fig. 10h–j). Functionally, recombinant SERPINA3N enhanced LPS-induced expression of multiple TLR4 downstream inflammatory genes, whereas this effect was significantly blunted by the selective TLR4 inhibitor TAK-242. Together, these findings support a direct mechanistic link between oligodendrocyte-derived SERPINA3N and microglial pro-inflammatory activation. Given that cultured microglia lack detectable SERPINA3N expression, our data support a model in which oligodendrocyte-derived SERPINA3N acts directly on microglia and potentiates inflammatory activation through TLR4-dependent signaling. However, the precise molecular mechanisms by which SERPINA3N modulates TLR4 signaling remain elusive. One possibility is that SERPINA3N may regulate TLR4 availability by inhibiting serine protease activity required for TLR4 cleavage^61^. Future studies are needed to prove this possibility.

To summarize, our findings nominate SerpinOLs as a subpopulation of injury-transduced OLs. Direct injury to OLs, rather than neuroinflammation, underlies the state transition of SerpinOLs. Molecularly, STAT3-mediated signaling is a trigger for SERPINA3N expression in SerpinOLs. Functionally, SerpinOLs perpetuate neuroinflammation and promote microglia toward pro-inflammatory and disease-associated states, thereby exacerbating demyelination, impeding remyelination, and worsening functional outcomes. Thus, targeting SerpinOLs and their transcriptomic profiles represents a promising avenue for modulating CNS inflammatory response and preserving tissue integrity in various CNS pathologies.

## Methods

### Transgenic mice

All transgenic mice were maintained on a C57BL/6 background and housed in a 12 h light/dark cycle with ad libitum access to water and food. Both males and females were used in this study unless otherwise indicated. Sex was considered in the study design by including both males and females across experimental groups where possible. Data were not analyzed separately by sex because the study was not powered to detect sex-specific effects. All animal procedures were approved by the UC Davis Institutional Animal Care and Use Committee (IACUC; protocol nos. 23771 and 23773) and were performed in accordance with institutional guidelines.

### A total of 13 transgenic lines

A total of 13 transgenic lines were used in the study, including *Serpina3n-tdTom* reporter line we generated by our own group, *Plp-CreER*^*T2*^ (RRID:IMSR_JAX:005975), *Aldh1l1-CreER*^*T2*^ (RRID:IMSR_JAX:031008), *Aldh1l1-eGFP* (RRID:IMSR_JAX:030247), *RiboTag* (RRID:IMSR_JAX:029977), *Olig2-Cre* (RRID:IMSR_JAX:025567), *Sox10-Cre* (RRID:IMSR_JAX:025807), *Serpina3n-floxed* (RRID:IMSR_JAX:027511), *Stat3-floxed* (RRID:IMSR_JAX:016923), *Rosa26-eGFP-DTA* (RRID:IMSR_JAX:032087), *5xFAD* (RRID:MMRRC_034848-JAX), *Cx3cr1-GFP/Ccr2-RFP* (RRID:IMSR_JAX:032127), and *Cnp-Cre:R26Stop*^*FL*^*ikk2ca* (referred to as NFkB CA mice) was from our previous study^36^.

Serpina3n-tdTom knock-in reporter mouse line was generated using CRISPR/Cas9-based Extreme Genome Editing (EGE) technology. A single sgRNA was designed to target exon 5 of the mouse *Serpina3n* gene. The targeting construct was assembled using the TV-4G vector and included a ~1.5 kb 5′ ******homologous arm, exons 4–5 fused with a P2A-tdTomato cassette, and a ~1.5 kb 3′ homologous arm. The targeting vector DNA, *Cas9* mRNA, and sgRNA were microinjected into zygotes derived from C57BL/6J mice. The injected embryos were subsequently transferred into pseudopregnant female mice. The P2A-tdTomato cassette was precisely inserted into exon 5, immediately upstream of the 3′ untranslated region (3′UTR) of the *Serpina3n* gene. F0 positive founder mice were bred with wild-type C57BL/6J mice to obtain F1 offspring. Germline transmission in F1 heterozygous mice was confirmed by PCR, DNA sequencing, and Southern blot analysis using probes at both the 5′ and 3′ ends to exclude random integration events. The DNA template for PCR was prepared using: EGE-YQL-0142-A-WT-F: TCCCTGGTCTCCACTGAGTGATGTG; EGE-YQL-0142-A-WT-R: GCTACCAAGAAGTCTGGGAGCATGG and tdtomato-15R: AACTCTTTGATGACCTCCTCG (see Fig. S4).

Animal genotype was determined by PCR of genomic DNA extracted from tail tissue.

### Mouse models of CNS diseases and injuries

#### MOG/EAE model

Both male and female mice were used in this study. EAE was induced in 12–15-week-old mice using two subcutaneous flank injections of a total of 300 µg of MOG peptide (35–55) emulsified in complete Freund’s adjuvant [incomplete Freund’s adjuvant (Thermo Fisher) containing 5 mg/ml of heat-killed Mycobacterium tuberculosis (BD Difco Adjuvants)]. On days 0 and 2 after induction, mice also received intraperitoneal injections of 200 ng of pertussis toxin. CFA control mice received only CFA and pertussis toxin, without MOG peptide. Mice were weighed and assessed for clinical symptoms of classical EAE on a 5-point scale using our published protocols^62^. Mice were scored daily as follows: 0, no detectable signs of EAE; 0.5, distal limp tail; 1.0, limp tail or waddling gait; 1.5, limp tail and waddling gait; 2.0, unilateral partial hindlimb paresis; 2.5, bilateral partial hindlimb paresis; 3.0, complete bilateral hindlimb paresis; 3.5, partial hindlimb paralysis; 4.0, complete hindlimb paralysis; and 5, moribund. At the indicated timepoints, spinal cords were extracted from mice in each group. Imaging and quantifications were performed in the ventral white matter of the lumbar spinal cord. For single-cell RNA sequencing, lumbar segments from three mice were pooled and processed together using the 10x Genomics Chromium platform.

#### Cuprizone (CPZ) model

Mice were fed 0.3% (w/w) CPZ (bis-cyclohexanone oxaldihydrazone, Inotiv, #TD.140805) mixed into standard powdered rodent chow for 2 days to 4 weeks to induce demyelination. Food consumption was monitored, and food was changed twice weekly. Control mice were fed standard powdered mouse chow for up to 4 weeks. Mice were euthanized after 2 days or 4 weeks of CPZ treatment, and brain tissues were collected for cell sorting, molecular analyses, and various histological examinations.

#### LPS endotoxicity

6–8-week-old mice were injected intraperitoneally (i.p.) with 3 mg/kg of LPS (Cat# L2880, *E. coli* O55:B5, Sigma-Aldrich, USA) or PBS (Cat# 10010023, Gibco, USA) for endotoxin challenge^28^.

#### Photothrombosis-induced focal cerebral ischemia

Photothrombotic focal cerebral ischemia was induced according to our previously published protocol^31^. Mice were anesthetized with ketamine and xylazine at doses of 130 and 10 mg/kg body weight, respectively. The photosensitizer Rose Bengal (RB) was prepared in artificial cerebrospinal fluid (ACSF) and delivered intravenously through the tail vein at 0.03 mg/g body weight. To induce focal ischemia, the designated area at the center of the craniotomy was illuminated for 2 min with green light (535 ± 25 nm) from a mercury lamp through a 10× objective (numerical aperture, 0.3).

#### 5xFAD mouse model for Alzheimer’s disease

To investigate SERPINA3N expression in the context of Alzheimer’s disease, we utilized 7-8-month-old hemizygous 5xFAD transgenic mice, which are a widely used model of aggressive amyloid pathology. These mice overexpress mutant human amyloid beta (A4) precursor protein 695 (APP) harboring three Familial Alzheimer’s Disease (FAD) mutations, Swedish (K670N/M671L), Florida (I716V), and London (V717I), along with a human presenilin 1 (PSEN1) transgene carrying two additional FAD mutations (M146L and L286V). 5xFAD mice were obtained from the Mutant Mouse Resource and Research Center (MMRRC) (RRID: MMRRC_034848-JAX) and maintained on a C57BL/6J background by crossing hemizygous 5xFAD mice with wild-type C57BL/6J mice (RRID: IMSR_JAX:000664). Genotyping of the 5XFAD transgene, located on mouse chromosome 3, was performed using the following primers: common forward 5′-ACC CCC ATG TCA GAG TTC CT-3′, wild-type reverse: 5′-TAT ACA ACC TTG GGG GAT GG-3′, and mutant reverse: 5′-CGG GCC TCT TCG CTA TTA C-3′. PCR amplification yields a 129 bp band for the transgene and a 216 bp band for the wild-type allele. Hemizygous mice exhibit both bands, whereas wild-type mice show only the 216 bp band.

#### Spinal cord injury

The T7/T10 double lateral hemisection injury was performed as previously reported in our study^63^. Mice were anesthetized by isoflurane inhalation (4% for induction and 2% for maintenance) in O₂ throughout the surgical procedure. Following exposure of the thoracic spinal cord by laminectomy from T7 to T10, a right-sided over-hemisection was created at T7 using a scalpel and micro-scissors. The lesion included the dorsal columns bilaterally and extended through the contralateral ventral spinal cord to ensure complete disruption of the ventral pathways (Supplementary Fig. 9a). A second lesion was subsequently made at T10, where the left half of the spinal cord was transected to the midline using a scalpel and micro-scissors. Following completion of the lesions, the overlying muscle layers were closed with sutures, and the skin incision was secured with wound clips. Animals were sacrificed 10 weeks after surgery.

#### Normal aging

To determine whether OLs are a source of SERPINA3N during normal aging, we generated transgenic *Olig2-Cre:Serpina3n*^*fl/fl*^ mice (oligodendroglial *Serpina3n* cKO). We analyzed three experimental groups: young control mice (2 months old), aged wild-type mice (20 months old), and aged *Serpina3n* cKO mice (20 months old, *Olig2-Cre:Serpina3n*^*fl/fl*^). Mice were euthanized at the designated ages, and their forebrains were dissected and collected for downstream histological, molecular, and biochemical analyses.

#### DTA mouse model for cell-type-specific injury

To induce targeted injury of oligodendrocytes or astrocytes, we employed a *Cre/loxP*-based genetic model using the *Rosa26-eGFP-DTA* allele (RRID:IMSR_JAX:032087). This allele contains a *loxP*-flanked STOP cassette (*EGFP*::PGK-neo::3xpolyA) upstream of the diphtheria toxin A subunit (DTA) gene. In the absence of Cre, EGFP is ubiquitously expressed, while DTA expression is silenced by the transcriptional stop sequence. Upon *Cre*-mediated recombination, the STOP cassette is excised, resulting in loss of EGFP and activation of DTA expression, leading to selective ablation of *Cre*-expressing cells. For oligodendrocyte-specific ablation, *Rosa26-eGFP-DTA* mice were crossed with *Plp-CreER*^T2^ mice to generate *Plp-DTA* double transgenic mice. For astrocyte-specific ablation, *Rosa26-eGFP-DTA* mice were crossed with Aldh1l1*-CreER*^*T2*^ mice to generate *Aldh1l1-DTA* mice. Adult mice (8-10 weeks old) received a single intraperitoneal injection of tamoxifen (100 mg/kg) to induce Cre recombination. Mice were sacrificed 24 h after tamoxifen administration, and brain and spinal cord tissues were collected for analysis.

#### Tissue preparation, immunohistochemistry (IHC) and quantification

Mice were deeply anesthetized with a ketamine/xylazine mixture and transcardially perfused with ice-cold PBS. Collected tissues were either immediately snap-frozen on dry ice for RNA or protein extraction, or post-fixed in freshly prepared 4% paraformaldehyde (PFA; Electron Microscopy Sciences) for histological analysis. Fixed tissues were post-fixed for an additional 2 h at room temperature (RT), then washed in PBS (3 × 15 min), cryoprotected in 30% sucrose (Fisher Chemical) overnight at 4 °C, and embedded in O.C.T. compound (VWR International). Serial coronal cryosections (12 μm) were cut using a Leica Cryostat (CM 1900-3-1) and stored at −80 °C until use. For IHC, sections were air-dried at RT for 2 h, then blocked in 10% donkey serum diluted in PBS containing 0.1% Triton X-100 for 1 h at RT. Sections were incubated with primary antibodies overnight at 4 °C, followed by PBS with 0.1% Tween-20 (PBST) washes and incubation with fluorescently labeled secondary antibodies for 2 h at RT. DAPI was used as a nuclear counterstain. Images were acquired using a Nikon A1 confocal microscope. Confocal Z-stacks were obtained at 1 μm intervals (total thickness: 10 μm), and maximum intensity projections were generated for image analysis and quantification. To identify the cellular source of SERPINA3N, we quantified the percentage of SERPINA3N⁺ cells co-expressing specific lineage markers including GFAP (astrocytes), SOX10 (oligodendrocytes), and CD68 (activated microglia/macrophages). SERPINA3N^+^ cells that did not co-localize with any of these markers were categorized as other. Quantification was performed on IHC images from at least three sections per animal (minimum *n* ≥ 4 animals per group, as indicated in the figure legends), and data are reported as the percentage of SERPINA3N⁺ cells in each category. Antibodies used for IHC are listed below: Goat polyclonal anti-Serpina3n (1:200, R&D System, Cat# AF4709; RRID:AB_2270116), Rabbit polyclonal anti-IBA1 (1:100, WAKO, Cat# 019-19741; RRID:AB_839504), Rabbit polyclonal anti-SOX10 (1:200, Abcam, Cat# ab27655; RRID: AB_778021), Mouse monoclonal anti-GFAP (1:200, or 1:20,000, Agilent, Cat# Z0334; RRID:AB_10013382), Rat monoclonal anti-CD68 (1:200, Bio-Rad, Cat# MCA1957; RRID: AB_3100585), Mouse monoclonal anti-APC (Ab-7) (CC1) (1:100, Millipore, Cat#OP80; RRID: AB_213434), Rabbit monoclonal anti-TCF4/TCF7L2 (C48H11) (1:100, Cell Signaling Technology, Cat#2569S; RRID:AB_2199816), Rabbit recombinant monoclonal anti-Stat3 (1:100, Cell Signaling, Cat# 4904, RRID:AB_331269), Rabbit recombinant monoclonal anti-p-STAT3 (Tyr705) (1:100, Cell Signaling, Cat# 9145; RRID:AB_2491009), Rabbit polyclonal anti-RFP (1:200, Rockland, Cat# 600-401-379; RRID:AB_2209751), Mouse monoclonal anti-Aβ1-16 (1:100, clone 6E10, BioLegend, Cat# 803001; RRID:AB_2715854), Rat monoclonal anti-I-A/I-E (1:100, BD Pharmingen, Cat# 556999; RRID:AB_396545), Mouse monoclonal anti-NeuN (1:100, Millipore, Cat# MAB377; RRID:AB_2298772), Rabbit recombinant monoclonal anti-phospho-p65 (Ser536) (1:100, Cell Signaling Technology, Cat# 3033, RRID:AB_331284), Mouse monoclonal anti-CD45 (1:100, Thermo Fisher Scientific, Cat# 14-0451-82; RRID:AB_467251), Rabbit polyclonal anti-CD74 (1:100, Thermo Fisher Scientific, Cat# PA5-22113; RRID:AB_11157006), Chicken polyclonal anti-GFP (1:200, Abcam, Cat# ab13970; RRID:AB_300798), Mouse monoclonal anti-PLP1 (1:200, Thermo Fisher, Cat# MA1-80034; RRID: AB_2299793), Rat monoclonal anti-MBP (1:200, Novus, Cat# NB600-717; RRID: AB_2139899), Mouse anti-GST-π (1:100, BD Bioscience, Cat# 610718; RRID: AB_398042), Mouse monoclonal anti-SMI32 (1:200, Covance, Cat# SMI-32R-100, RRID:AB_509997), Mouse monoclonal anti-4 Hydroxynonenal (4HNE, 1:100, Abcam, Cat# ab46545, RRID: AB_722490). All secondary antibodies used for IHC were from Jackson ImmunoResearch Laboratories.

#### Black-Gold II myelin staining

Black-Gold II myelin stain (Biosensis, Cat# TR-100-B) was performed according to the manufacturer’s instructions. Fixed, frozen sections (12 um) were incubated with 0.3% Black-Gold II stain for 20 min at 60 °C. Staining was stabilized with 1% sodium thiosulfate solution, followed by rinsing, dehydration through a graded ethanol series (50%, 75%, 85%, 95% and 100%), clearing in xylene, and coverslipping with mounting medium (Fisher Scientific). Images were acquired on an Olympus BX61 microscope, and staining intensity was quantified using ImageJ (NIH).

#### RNA extraction, cDNA preparation, RT-qPCR, and primers

Total RNA was isolated using the RNeasy Lipid Tissue Mini Kit (QIAGEN) according to the manufacturer’s instructions, including on-column DNase digestion using the RNase-Free DNase Set (QIAGEN) to eliminate genomic DNA contamination. RNA concentration and purity were assessed using a NanoDrop 2000 Spectrophotometer (Thermo Fisher Scientific). Complementary DNA (cDNA) was synthesized using the Omniscript RT Kit (QIAGEN). Quantitative real-time PCR (RT-qPCR) was carried out using the QuantiTect SYBR® Green PCR Kit (QIAGEN) on an Agilent MP3005P thermocycler. Gene expression was normalized to the internal control gene Hsp90, and relative expression levels were calculated using the 2^−ΔCt method: ΔCt = Ct(Hsp90) - Ct(target gene). Primer sequences used for RT-qPCR are listed below: *Serpina3n* (F/R, GCCTCGTCAGGCCAAAAAG/TGAACGTGTCAAGAGGGTCAA), *Cxcl10* (CCCACGTGTTGAGATCATTG/CACTGGGTAAAGGGGAGTGA); *C3* (AGCTTCAGGGTCCCAGCTAC/GCTGGAATCTTGATGGAGACGC); *IL-1b* (GAAATGCCACCTTTTGACAGTG/CTGGATGCTCTCATCAGGACA); *Ccl2* (CACTCACCTGCTGCTACTCA/GCTTGGTGACAAAAACTACAGC); *Cd68* (TGTCTGATCTTGCTAGGACCG/GAGAGTAACGGCCTTTTTGTGA); *Aldh1l1* (GCAGGTACTTCTGGGTTGCT/GGAAGGCACCCAAGGTCAAA); *P2ry12* (CCCTGTGCGTCAGAGACTAC/CAAGCTGTTCGTGATGAGCC); *Tnfα* (TGTGCTCAGAGCTTTCAACAA/CTTGATGGTGGTGCATGAGA); *iNos* (CCCTTCAATGGTTGGTACATG/ACATTGATCTCCGTGACAGCC); *Cd86* (GAGCGGGATAGTAACGCTGA/ GGCTCTCACTGCCTTCACTC); *Cd206* (CTTCGGGCCTTTGGAATAAT/TAGAAGAGCCCTTGGGTTGA); *Ym1* (CAGGTCTGGCAATTCTTCTGAA/GTCTTGCTCATGTGTGTAAGTGA); *P21* (TCTTGCACTCTGGTGTCTGA/CTGCGCTTGGAGTGATAGAA); *Ptgs2* (GCTGTACAAGCAGTGGCAAA/ CCCCAAAGATAGCATCTGGA); *Hsp90* (AAACAAGGAGATTTTCCTCCGC/CCGTCAGGCTCTCATATCGAAT).

#### Protein preparation and Western blot

Tissues were lysed in N-PER Neuronal Protein Extraction Reagent (Thermo Fisher), supplemented with protease and phosphatase inhibitor cocktail (Thermo Fisher) and PMSF (Cell Signaling Technology). Lysates were incubated on ice for 10 min and centrifuged at 10,000 × *g* for 10 min at 4 °C. Protein concentrations were determined using a BCA protein assay kit (Thermo Fisher Scientific). Equal amounts of protein (30 μg per sample) were resolved by SDS-PAGE using AnykD Mini-PROTEAN TGX precast gels (Bio-Rad) and transferred to 0.2 μm nitrocellulose membranes (Bio-Rad) using the Trans-Blot Turbo Transfer System (Bio-Rad). Membranes were blocked with 5% BSA (Cell Signaling Technology) for 1 h at room temperature and incubated overnight at 4 °C with primary antibodies. After washing, membranes were incubated with appropriate HRP-conjugated secondary antibodies and visualized using Western Lightning Plus ECL (PerkinElmer). Band intensities were quantified using NIH ImageJ software. Antibodies used for Western blot are listed below: Goat polyclonal anti-SERPINA3N (1:1000, R&D System, Cat# AF4709; RRID:AB_2270116), Rabbit polyclonal anti-IBA1 (1:1000, WAKO, Cat# 019-19741; RRID:AB_839504), Rabbit polyclonal anti-GFAP (1:1000, Millipore, Cat# MAB360; RRID:AB_11212597), Rat monoclonal anti-CD68 (1:1000, Bio-Rad, Cat# MCA1957; RRID: AB_3100585), Mouse monoclonal anti-PLP1 (1:1000, Thermo Fisher, Cat# MA1-80034; RRID: AB_2299793), Rat monoclonal anti-MBP (1:1000, Novus, Cat# NB600-717; RRID: AB_2139899), Mouse monoclonal anti-β-ACTIN (1:1000, Cell Signaling Technology, Cat# 3700; RRID:AB_2242334), Rabbit monoclonal anti-GAPDH (1:1000, Cell Signaling Technology, Cat# 2118, RRID:AB_561053). All HRP-conjugated secondary antibodies were from Thermo Fisher Scientific. Uncropped and unprocessed blot images are provided in the Source Data file.

#### RiboTag RNA immunoprecipitation

To analyze ribosome-bound transcripts in oligodendrocytes and astrocytes, *Plp1-CreER*^*T2*^ and *Aldh1l1-CreER*^*T2*^ transgenic lines were crossed with RiboTag mice (RRID:IMSR_JAX:029977). Adult mice received intraperitoneal tamoxifen injections once daily for five consecutive days. Starting 14 days after the final injection, mice were maintained on either a CPZ diet or a normal diet for 4 weeks prior to tissue collection. Brains were rapidly extracted and homogenized on ice using a Micro-Tube Homogenizer System in ice-cold homogenization buffer (10% w/v) containing: 50 mM Tris-HCl (pH 7.4), 100 mM KCl, 12 mM MgCl₂, 1% NP-40, 1 mM DTT, 0.1 mg/ml cycloheximide (Sigma), 200 units/ml RNasin (Promega), and cOmplete protease inhibitor cocktail (MilliporeSigma), all prepared in RNase-free water. Homogenates were centrifuged at 10,000 × *g* for 10 min at 4 °C to remove cellular debris. Supernatants were transferred to fresh microcentrifuge tubes kept on ice. A 10 µl aliquot was saved as the input control. For immunoprecipitation, 5 µl of anti-hemagglutinin (HA) antibody (Covance Anti-HA.11 Epitope Tag Antibody) or 1 µg of normal IgG control was added to the remaining 400 µl of cleared lysate. Samples were incubated for 4 h at 4 °C with gentle rotation. Protein A/G magnetic beads (Thermo Fisher Scientific, Cat. No. 88803; 100 µl per sample) were washed three times with homogenization buffer, equilibrated, added to the samples, and incubated overnight at 4 °C with rotation. The next day, samples were washed three times with high-salt buffer (50 mM Tris-HCl, 300 mM KCl, 12 mM MgCl₂, 1% NP-40, 1 mM DTT, 100 units/ml RNasin, 0.1 mg/ml cycloheximide, and 1:200 protease inhibitor in RNase-free water), with each wash lasting 5 min on a rotator in a cold room. After the final wash, magnetic beads were collected using a magnetic stand, and residual buffer was removed. Beads were resuspended in RLT plus lysis buffer, and RNA was extracted using the RNeasy Plus Micro Kit (Qiagen, Cat. No. 74034) following the manufacturer’s protocol.

#### Magnetic-activated cell sorting (MACS)

Single-cell suspensions were generated from mouse brain tissues using the Adult Brain Dissociation Kit (Mouse and Rat; Cat# 130-107-677, Miltenyi Biotec, Germany) and a gentleMACS Dissociator (Cat. No. 130-093-235, Miltenyi Biotec, Germany). Brain tissues were collected, cut into approximately 0.5 cm pieces, and placed in prewarmed gentleMACS C Tubes (Cat# 130-093-237, Miltenyi Biotec, Germany). Tissue dissociation was initiated by adding 1950 μL of enzyme mix 1, containing Enzyme P and Buffer Z, followed by mechanical dissociation using the appropriate gentleMACS program. After the initial dissociation cycle, 30 μL of enzyme mix 2, containing Enzyme A and Buffer Y, was added, and the samples underwent two additional gentle dissociation cycles at 37 °C. The resulting cell suspensions were briefly centrifuged and passed through a 70-μm MACS SmartStrainer to remove undissociated tissue and cell aggregates. The strainer was subsequently rinsed with 10 mL of D-PBS (Cat# 14287-080, Gibco) to maximize cell recovery.

Following centrifugation, the resulting cell pellets were subjected to magnetic bead-based isolation of specific glial cell populations. Anti-CD11b MicroBeads (Cat# 130-093-634, Miltenyi Biotec, Germany) and anti-O4 MicroBeads (Cat# 130-094-543, Miltenyi Biotec, Germany) were used for the isolation of CD11b-positive and O4-positive cells, respectively. For astrocyte isolation, cells were first incubated with FcR Blocking Reagent, followed by labeling with anti-ACSA-2 MicroBeads (Cat# 130-097-678, Miltenyi Biotec, Germany). Cells were incubated with the corresponding MicroBeads for 15 min at 4 °C and subsequently washed with 0.5% BSA/PBS. After centrifugation at 300 × *g* for 10 min, the cell pellets were resuspended in 500 μL of 0.5% BSA/PBS for subsequent magnetic separation.

For magnetic separation, an LS Column was placed in a MACS Separator (Cat# 130-090-976, Miltenyi Biotec) and equilibrated with 3 mL of 0.5% BSA/PBS. The cell suspension was loaded onto the column, followed by three washes with 3 mL of 0.5% BSA/PBS. The unlabeled cell fraction was recovered in the flow-through, whereas the magnetically retained cells were eluted after removal of the column from the magnetic field by applying the supplied plunger. The isolated cells were lysed in 350 μL of Buffer RLT Plus supplemented with β-mercaptoethanol (β-ME) and stored at −80 °C until further analysis.

#### RNA sequencing

For bulk RNA-seq, mRNA was enriched from high-quality total RNA (RIN > 7) using oligo(dT)-attached magnetic beads. The enriched poly(A) RNA underwent fragmentation, followed by first- and second-strand cDNA synthesis with dUTP incorporation to maintain strand specificity. The cDNA was end-repaired, 3′-adenylated, and ligated to bubble-shaped adapters before PCR amplification. The resulting PCR products were denatured, circularized using a bridged primer, and amplified via phi29 polymerase to form DNA nanoballs (DNBs). These DNBs were loaded onto patterned nanoarrays and sequenced on an MGI T7 system, producing paired-end 150 bp reads. After sequencing, raw reads were quality-checked and aligned to the Mus musculus reference genome (GCF_000001635.27_GRCm39) using HISAT or Bowtie2 (v2.3.4.3), then quantified by RSEM (v1.3.1). Differentially expressed genes (DEGs) were identified (e.g., DESeq2, threshold Q ≤ 0.05 or FDR ≤ 0.001). Subsequent analyses, including principal component analysis, correlation, and GO/KEGG enrichment, were conducted to interpret gene expression patterns.

For scRNA-seq, a single-cell suspension was prepared from approximately 50–100 mg of fresh tissue via homogenization, filtration, and low-speed centrifugation. After confirming >80% cell viability by trypan blue, each suspension was processed on the 10x Genomics Chromium platform, generating GEM (Gel Bead in Emulsion) droplets with unique barcodes and UMIs. Reverse transcription was carried out within each droplet, followed by cDNA purification, amplification, fragmentation, end-repair, adapter ligation, and PCR indexing. Libraries were circularized and amplified into DNA nanoballs before sequencing on the DNBSEQ G400 system (PE28 + 100, ~350 M reads per sample). Resulting reads were aligned to the Mus musculus reference genome (refdata-gex-mm10-2020-A) using Cell Ranger (v5.0.1)^64^. Seurat (v3.2.0)^65^ was employed for downstream quality filtering by excluding cells with <200 detected genes and cells with mitochondrial transcript fraction in the top 15%; predicted doublet excluded using DoubletDetection^66^. A set of 2000 highly variable genes was used for principal component analysis (PC = 15), followed by dimensionality reduction with UMAP. Cluster marker genes were identified using the FindAllMarkers function (logfc.threshold > 0.25, min.pct > 0.1, Padj ≤ 0.05), and SCSA^67^ was utilized to assist in cell-type annotation.

#### Gene set score calculation

For each defined gene set, expression values were first transformed via log₂(expression+1) to stabilize variance and mitigate the impact of highly expressed genes. In cases where all genes were anticipated to move in a consistent direction, the sample score was calculated as the arithmetic mean of the log-transformed values. Where both up- and downregulated genes were involved, each gene was assigned a + 1 or −1 weight based on its presumed direction of regulation, and the final score was obtained by averaging these weighted, log-transformed values. If baseline correction was needed, the mean gene set score of control samples was subtracted from each test sample. All analyses were conducted in R (v4.4.0).

#### Primary oligodendrocyte culture, differentiation, and treatment

Primary OPCs were isolated from cerebral cortices of postnatal day 0 to 2 mice of mixed sex^68^. Cerebral cortices were dissected, meninges were removed, and the tissue was enzymatically digested using papain (20 U/mL; Worthington) supplemented with DNase I (250 U/mL; Sigma) and D-(+)-glucose (0.36%; AMRESCO) for 1 h at 37 °C. The tissue was then mechanically triturated to obtain a single-cell suspension. Cells were resuspended in DMEM containing 10% heat-inactivated fetal bovine serum (FBS) and penicillin/streptomycin (P/S) and plated onto poly-D-lysine (PDL; Millipore)-coated 10-cm dishes. After 24 h, cultures were washed with HBSS to remove debris, and the medium was replaced with serum-free growth medium (GM) consisting of 30% B104 neuroblastoma conditioned medium and 70% N1 medium [DMEM supplemented with 5 μg/mL insulin (Sigma), 50 μg/mL apo-transferrin (Sigma), 100 μM putrescine (Sigma), 30 nM sodium selenite (Sigma), and 20 nM progesterone (Sigma)]. Cells were cultured until reaching approximately 80% confluency. Mixed glial cells were then dissociated into a single-cell suspension and subjected to immunopanning. The suspension was first incubated on dishes coated with anti-Thy1.2 (CD90.2) antibody (10 μL; BioLegend) to deplete astrocytes, neurons, and meningeal cells. The non-adherent fraction was subsequently transferred to dishes coated with anti-NG2 antibody (5 μL; Millipore) to select for OPCs. Isolated OPCs were plated onto PDL-coated plates in OPC proliferation medium [GM supplemented with 5 ng/mL FGF (PeproTech), 4 ng/mL PDGF-AA (PeproTech), 50 μM forskolin (PeproTech), and GlutaMAX (Thermo Fisher)]. To induce differentiation, the medium was switched to differentiation medium (DM) consisting of DMEM/F12 supplemented with 12.5 μg/mL insulin, 100 μM putrescine, 24 nM sodium selenite, 10 nM progesterone, 10 ng/mL biotin, 50 μg/mL transferrin (Sigma), 30 ng/mL 3,3′,5-Triiodo-L-thyronine (T3; Sigma), 40 ng/mL L-Thyroxine (T4; Sigma), GlutaMAX, and P/S. Cultures were maintained at 37 °C in a humidified atmosphere containing 5% CO_2_. To induce OL injury, differentiated OLs were treated with 100 μM H_2_O_2_ or vehicle control for 24 h, after which cells were harvested for ChIP-qPCR analysis.

#### Chromatin immunoprecipitation (ChIP) assay

ChIP-qPCR assay was performed using the SimpleChIP Enzymatic Chromatin IP Kit (Cat# 9003; Cell Signaling Technology)^69^. Primary OLs were crosslinked with 1% formaldehyde for 10 min at room temperature, followed by quenching with 125 mM glycine for 5 min. Cells were washed twice with ice-cold PBS, harvested in PBS containing a protease inhibitor cocktail, and lysed to isolate nuclei. Extracted nuclei were incubated with Micrococcal Nuclease to digest chromatin into fragments ranging from 150 to 900 bp. The nuclear membrane was subsequently disrupted by sonication. The digested chromatin was subjected to immunoprecipitation (IP) with an anti-STAT3 antibody (Cat #9139, Cell Signaling Technology) or normal mouse IgG control overnight at 4 °C with rotation. ChIP-Grade Protein G magnetic beads were added to each sample and incubated for 2 h at 4 °C with rotation. Following high-salt and low-salt washes, chromatin was eluted from the antibody/bead complex by incubation at 65 °C for 30 min with vortexing. Protein-DNA cross-links were reversed by incubation with Proteinase K overnight at 65 °C. The DNA was purified using spin columns and analyzed by RT-qPCR. Two putative STAT3 binding sites were identified within the *Serpina3n* promoter region relative to the TSS: Site 1 (5′-TTACCAGAA-3′) located at −144 to −135 bp, and Site 2 (5′-TTGAGAGAA-3′) located at −1890 to −1882 bp. The specific primer sequences used for amplification were as follows: Site 1: Forward 5′-AGGAGTTCTGATAGAGGCTACA-3′; Reverse 5′-GGCTCCTCCCTCCTCTTAG-3′. Site 2: Forward 5′-GCAGAGTGAGGGACAGCAT-3′; Reverse 5′-CCATTACTTCAACCAGGCTCTG-3′. The enrichment of STAT3 binding was quantified relative to the IgG negative control and is presented as fold change.

#### Primary microglia culture and SERPINA3N/TAK-422 treatment

Primary cortical cell cultures were prepared from postnatal day 0–2 mouse pups. Mouse pups were quickly decapitated with sterile scissors, and then the heads were immediately transferred into ice-cold saline solution. The meninges were carefully removed under an anatomical microscope, and the cortices were then mechanically dissociated with surgical instruments and transferred with a 10 mL pipette to a sterile 50 mL conical tube. After centrifugation, cortical tissues were treated with a papain dissociation kit (Cat# LK003176, Worthington, USA) supplemented with DNase I (250 U/mL, Cat# D5025, Sigma-Aldrich, USA) and D-(+)-glucose (0.36%, Cat# 0188, AMRESCO, USA), and incubated in a 36 °C/10% CO_2_ chamber for 90 min. Digested tissues were pipetted up and down with a sterile Pasteur pipette and then flushed through the cell strainer. Strained cells were centrifuged at 300 × *g* for 10 min. Cells were then plated on poly-D-lysine (PDL, Cat# A003-E, Millipore, USA)-coated T-75 flask (Cat# 430641U, Corning, USA) by adding DMEM/F12 (Cat# 1196092, Thermo Fisher, USA) with 10% heat-inactivated fetal bovine serum (FBS, Cat# 12306-C, Sigma-Aldrich, USA) and 1% penicillin/streptomycin (P/S, Cat# 15140122, Thermo Fisher, USA). The plates were incubated in a 5% CO_2_ incubator at 37 °C for 10–14 days to grow a confluent mixed glial population. After the mixed glial culture was completely confluent, a mechanical shaking method was used to isolate microglia (180 rpm at 37 °C without CO_2_ for 2 h). After shaking, culture medium containing microglia in the T-75 flask was transferred to 50 mL tubes. Microglia were washed with PBS, and cell count was performed using a hemocytometer with Trypan Blue exclusion. Next, microglia were resuspended in DMEM/F12 containing 10% FBS, 1% P/S, and microglia supplement (Cat# 1952, ScienCell, USA) and seeded appropriately onto 6-well cell culture plates. All culture plates were incubated in a 37 °C incubator with 5% CO_2_ and the medium was changed every other day after seeding. Primary microglia at 14 days in vitro (DIV) were stimulated with LPS (10 ng/mL; Cat# L2880, *E. coli* O55:B5, Sigma-Aldrich, USA) for 24 h with or without recombinant SERPINA3N (50 ng/mL; Cat# 4709-PI, R&D System, USA) and/or the TLR4 inhibitor TAK-242 (0.2 μM; Cat# 614316, Sigma-Aldrich, USA). Vehicle control cells were treated with DMSO.

#### Proximity ligation assay (PLA)

Primary microglia were treated with or without LPS and recombinant SERPINA3N, fixed on glass slides, and subjected to a proximity ligation assay using the Duolink in Situ Red Starter Kit Mouse/Goat (Cat# DUO92103, Sigma-Aldrich, USA). Samples were lightly permeabilized with 0.02% Triton X-100 in PBS for 5 min, then blocked with the Duolink Blocking Solution and Fc block (CD16/CD32 antibody, Cat# 14-0161-82, Invitrogen) in a humidified chamber for 60 min at 37 °C. Samples were subsequently incubated with primary antibodies against TLR4 (Cat# NB100, NOVUS; AB_2205129) and SERPINA3N (Cat# AF4709, R&D System; RRID:AB_2270116), diluted in Duolink Antibody Diluent overnight at 4 °C. After washing in 1× Wash Buffer A, samples were incubated with the species-specific PLUS and MINUS PLA probes, each diluted 1:5 in antibody diluent, for 1 h at 37 °C. Slides were then washed and subjected to ligation using 1× ligation buffer containing ligase for 30 min at 37 °C, followed by amplification using 1× amplification buffer containing polymerase for 100 min at 37 °C. After amplification, slides were washed in 1× Wash Buffer B and 0.01× Wash Buffer B, mounted with Duolink in Situ Mounting Medium with DAPI, and imaged by confocal microscopy. Quantification was performed by calculating the number of PLA puncta per cell.

#### Co-Immunoprecipitation (Co-IP)

Primary microglia cultured for 14 DIV were stimulated with LPS in the presence of recombinant SERPINA3N for 24 h, washed three times with PBS to remove excess recombinant SERPINA3N, and then collected for Co-IP. To stabilize interactions between SERPINA3N and TLR4 prior to lysis, cells were crosslinked with DTSSP [3,3′-dithiobis(sulfosuccinimidyl propionate)], Cat# 21578, Thermo Fisher Scientific), a water-soluble, membrane-impermeable, cleavable crosslinker. DTSSP was freshly dissolved in cold PBS and added to the cells at a final concentration of 1 mM, followed by incubation for 30 min at 4 °C. The crosslinking reaction was quenched with 20 mM glycine for 15 min. Co-IP was performed using the Pierce Crosslink Magnetic IP/Co-IP Kit (Cat# 88805, Thermo Fisher Scientific) according to the manufacturer’s instructions. Cells were lysed in IP Lysis/Wash Buffer supplemented with protease and phosphatase inhibitor cocktail (Cat# 78440, Thermo Fisher Scientific). Lysates were clarified by centrifugation, and a portion of each sample was reserved as input. Protein concentration was determined, and equal amounts of total protein (1 mg per sample) were used for each immunoprecipitation. 10 μg of primary antibody or the corresponding isotype control IgG was covalently crosslinked to 25 μL of protein A/G magnetic beads. Equal amounts of protein lysate were then incubated overnight at 4 °C with antibody-crosslinked beads. After incubation, the beads were washed thoroughly to remove non-specifically bound material, and bound proteins were eluted using the kit’s low-pH elution buffer. Eluates were immediately neutralized with neutralization buffer. For western blot analysis, eluates were mixed with Lane Marker Sample Buffer containing β-mercaptoethanol. Under reducing conditions, the disulfide bond within DTSSP was cleaved, allowing release of crosslinked protein complexes for electrophoretic analysis. The primary antibodies and corresponding isotype controls used for Co-IP were as follows: anti-TLR4 (Cat# NB100, NOVUS; AB_2205129), anti-SERPINA3N (Cat# AF4709, R&D System; RRID:AB_2270116), normal mouse IgG (Cat# 68860, Cell Signaling Technology; RRID:AB_3675987), and normal goat IgG (Cat# AB-108-C, R&D Systems; RRID:AB_354267). Uncropped and unprocessed blot images are provided in the Source Data file.

#### Data collection and statistics

Data collection and quantification were performed by lab members blinded to mouse genotype and treatment. Both male and female mice were included in all experiments. Data are presented as mean ± standard error of the mean (s.e.m.) unless otherwise indicated. Scatter dot plots were used throughout the manuscript, with each dot representing a single mouse or one independent experiment. Data normality was assessed using the Shapiro–Wilk test. For comparison between two groups, an unpaired two-tailed Student’s *t*-test was used. The degrees of freedom are reported as t(df) in figure legends. For comparisons involving more than two groups, a one-way ANOVA followed by Tukey’s multiple comparisons test was performed. For comparisons involving two independent variables, a two-way ANOVA followed by Tukey’s multiple comparisons test was performed. The F-statistic and associated degrees of freedom are reported as F(DFn, DFd), where DFn and DFd represent the numerator and denominator degrees of freedom, respectively. Brown-Forsythe test was used to evaluate variance equality across multiple groups. For bulk RNA-seq differential expression analysis, differentially expressed genes (DEGs) were identified from raw read counts using DESeq2. Statistical significance was determined using the Wald test, and *P*-values were adjusted for multiple comparisons using the Benjamini–Hochberg procedure to control the false discovery rate (FDR). Adjusted *P*-values are reported as padj, which corresponds to the FDR. DEGs were defined using a threshold of FDR ≤ 0.05, unless otherwise indicated. For scRNA-seq marker gene analysis, cluster marker genes were identified using the FindAllMarkers function in Seurat v3.2.0 with logfc.threshold >0.25 and min.pct >0.1. Statistical significance was determined using the Wilcoxon rank-sum test, and *P*-values were adjusted for multiple comparisons. Marker genes were defined using an adjusted *P*-value threshold of Padj ≤ 0.05. Statistical analyses for non-sequencing data and data visualizations were conducted using GraphPad Prism version 8.0. A *p*-value of less than 0.05 was considered statistically significant. Significance is indicated in figures as follows: ∗*p* < 0.05, ∗∗*p* < 0.01, ∗∗∗*p* < 0.001; ns denotes not significant (*p* > 0.05).

Newly generated materials from this study are available from the corresponding authors upon request, subject to completion of an appropriate material transfer agreement where applicable.

### Reporting summary

Further information on research design is available in the Nature Portfolio Reporting Summary linked to this article.

## Supplementary information

Supplementary Information

Description of Additional Supplementary Files

Supplementary Data 1

Supplementary Data 2

Supplementary Data 3

Supplementary Data 4

Supplementary Data 5

Supplementary Data 6

Supplementary Data 7

Supplementary Data 8

Supplementary Data 9

Supplementary Data 10

Supplementary Data 11

Supplementary Data 12

Reporting Summary

Transparent Peer Review file

## Source data

Source Data

## Data Availability

The RNA-seq data generated and used in this study have been deposited in the Gene Expression Omnibus (GEO) database under the following accession codes: GSE287324, GSE287323, GSE287320, GSE287310, GSE287313 and GSE287309. Specifically, GSE287324 corresponds to Supplementary Data 1 and 4; GSE287323 corresponds to Supplementary Data 2; GSE287320 corresponds to Supplementary Data 3; GSE287310 corresponds to Supplementary Data 6–9; GSE287313 corresponds to Supplementary Data 10; and GSE287309 corresponds to Supplementary Data 12. Additional metadata and processed results, including gene set scores, are provided in the Supplementary Data files and/or Source Data file. Source data generated in this study are provided with this paper in the Source Data file. All other data supporting the findings of this study are available within the article, Supplementary Information, Supplementary Data files and Source Data file. Source data are provided with this paper.
