# Supplementary material for: Injury-transduced oligodendrocytes modulate neuroinflammation and glial activation in diseased and non-diseased central nervous system
Source: Nat Commun. 2026 Aug 29;17:10347. doi: 10.1038/s41467-026-76159-2 (PMC13623841; doi:10.1038/s41467-026-76159-2)
Supplement: Supplementary file 1 [file 41467_2026_76159_MOESM15_ESM.pdf]

nature portfolio

Corresponding author(s): Yan Wang, Fuzheng Guo

Last updated by author(s): Jun 7, 2026

Reporting Summary

Nature Portfolio wishes to improve the reproducibility of the work that we publish. This form provides structure for consistency and transparency in reporting. For further information on Nature Portfolio policies, see our Editorial Policies and the Editorial Policy Checklist.

Statistics

For all statistical analyses, confirm that the following items are present in the figure legend, table legend, main text, or Methods section.

n/a	Confirmed	
[ ]	[x]	The exact sample size (*n*) for each experimental group/condition, given as a discrete number and unit of measurement
[ ]	[x]	A statement on whether measurements were taken from distinct samples or whether the same sample was measured repeatedly
[ ]	[x]	The statistical test(s) used AND whether they are one- or two-sided<br>*Only common tests should be described solely by name; describe more complex techniques in the Methods section.*
[x]	[ ]	A description of all covariates tested
[ ]	[x]	A description of any assumptions or corrections, such as tests of normality and adjustment for multiple comparisons
[ ]	[x]	A full description of the statistical parameters including central tendency (e.g. means) or other basic estimates (e.g. regression coefficient) AND variation (e.g. standard deviation) or associated estimates of uncertainty (e.g. confidence intervals)
[ ]	[x]	For null hypothesis testing, the test statistic (e.g. *F*, *t*, *r*) with confidence intervals, effect sizes, degrees of freedom and *P* value noted<br>*Give P values as exact values whenever suitable.*
[x]	[ ]	For Bayesian analysis, information on the choice of priors and Markov chain Monte Carlo settings
[x]	[ ]	For hierarchical and complex designs, identification of the appropriate level for tests and full reporting of outcomes
[x]	[ ]	Estimates of effect sizes (e.g. Cohen's *d*, Pearson's *r*), indicating how they were calculated

Our web collection on statistics for biologists contains articles on many of the points above.

Software and code

Policy information about availability of computer code

Data collection	Bulk RNA-seq libraries were prepared using poly(A) enrichment and sequenced on on the MGI T7 T7 platform. Single-cell suspensions were processed using the 10x Genomics Chromium system and sequenced with the DNBSEQ G400 platform (PE28+100). Initial processing of of single-cell data, including quality control and UMI counting, was performed using Cell Ranger (v5.0.1). Preliminary analysis and visualization were provided by by BGI Genomics via the Dr. Tom Data Visualization Solution (https://www.bgi.com/global/service/dr-tom).<br><br>Fluorescence and confocal imaging were performed using a Nikon A1 A1 confocal microscope and an an Olympus BX61 microscope. Image processing was done using ImageJ (v1.52p, NIH) and Nikon NIS-Elements C software. Quantitative PCR was conducted using an an Agilent MP3005P thermocycler. Absorbance measurements were obtained using a SpectraMax i3x plate reader (Molecular Devices), and protein bands were imaged using the ChemiDoc MP MP Imaging System (Bio-Rad).
Data analysis	Bulk RNA-seq data were aligned to to the Mus musculus reference genome (GRCm39) using HISAT2 or or Bowtie2 (v2.3.4.3) and quantified with RSEM (v1.3.1). Differential expression analysis was performed using DESeq2.<br><br>Single-cell RNA-seq data were processed using Cell Ranger (v5.0.1) and analyzed in in R (v4.4.0) with Seurat (v3.2.0), including quality control, dimensionality reduction (UMAP), clustering, and marker gene identification. Doublets were excluded using DoubletDetection.<br><br>Custom R code was used specifically for gene set scoring. Log2(expression + 1) transformed expression values were averaged per sample and normalized against control means. The scoring logic followed the approach described in Pandey et et al., Cell Reports (2022; 40(8):111189). The R packages used included readxl, dplyr, tidyr, and writexl.

Additional single-cell transcriptomic analysis and visualization were performed using the Dr. Tom Data Visualization Solution (https://www.bgi.com/global/service/dr-tom) provided by BGI Genomics.

Image quantification and visualization were performed using ImageJ (v1.52p, NIH) and Nikon NIS-Elements C imaging software. Statistical analysis and graphical visualization were conducted using GraphPad Prism (v10.0.2, Dotmatics) and Microsoft Excel.

For manuscripts utilizing custom algorithms or software that are central to the research but not yet described in published literature, software must be made available to editors and reviewers. We strongly encourage code deposition in a community repository (e.g. GitHub). See the Nature Portfolio guidelines for submitting code & software for further information.

Data

Policy information about availability of data

All manuscripts must include a data availability statement. This statement should provide the following information, where applicable:

- Accession codes, unique identifiers, or web links for publicly available datasets
- A description of any restrictions on data availability
- For clinical datasets or third party data, please ensure that the statement adheres to our policy

Bulk and single-cell RNA sequencing data generated in this study have been deposited in the NCBI Gene Expression Omnibus (GEO) under the accession numbers GSE287309, GSE287310, GSE287313, GSE287320, GSE287323, and GSE287324. These datasets include both raw FASTQ files and processed gene expression matrices. All datasets are currently private and scheduled for public release on January 15, 2029.

Each GSE entry contains multiple samples (GSMs), including aging mouse brain, experimental autoimmune encephalomyelitis (EAE), and cuprizone-induced demyelination models, with detailed metadata provided in the GEO submission.

Reviewer access is available through the following links and tokens:
• GSE287309: https://www.ncbi.nlm.nih.gov/geo/query/acc.cgi?acc=GSE287309, token: ypmbaoiwzzilhoj
• GSE287310: https://www.ncbi.nlm.nih.gov/geo/query/acc.cgi?acc=GSE287310, token: alcpmgakxnuzlsh
• GSE287313: https://www.ncbi.nlm.nih.gov/geo/query/acc.cgi?acc=GSE287313, token: ufspkauyjrghfqn
• GSE287320: https://www.ncbi.nlm.nih.gov/geo/query/acc.cgi?acc=GSE287320, token: slelewcybzilnor
• GSE287323: https://www.ncbi.nlm.nih.gov/geo/query/acc.cgi?acc=GSE287323, token: wbgxqyaypnylhet
• GSE287324: https://www.ncbi.nlm.nih.gov/geo/query/acc.cgi?acc=GSE287324, token: itqvosukbbcxdan

Additional metadata and processed results, including gene set scores, are provided in the Supplementary Data files and/or Source Data file. Source data generated in this study are provided with this paper in the Source Data file. All other data supporting the findings of this study are available within the article, Supplementary Information, Supplementary Data files and Source Data file.

Research involving human participants, their data, or biological material

Policy information about studies with human participants or human data. See also policy information about sex, gender (identity/presentation), and sexual orientation and race, ethnicity and racism.

Reporting on sex and gender	N/A
Reporting on race, ethnicity, or other socially relevant groupings	N/A
Population characteristics	N/A
Recruitment	N/A
Ethics oversight	N/A

Note that full information on the approval of the study protocol must also be provided in the manuscript.

Field-specific reporting

Please select the one below that is the best fit for your research. If you are not sure, read the appropriate sections before making your selection.

[x] Life sciences   [ ] Behavioural & social sciences   [ ] Ecological, evolutionary & environmental sciences

For a reference copy of the document with all sections, see nature.com/documents/nr-reporting-summary-flat.pdf

Life sciences study design

All studies must disclose on these points even when the disclosure is negative.

Sample size	Sample sizes were chosen based on prior studies using similar mouse models and experimental approaches, our previous experience with comparable assays, feasibility considerations, and the need to include sufficient independent biological replicates for statistical analysis. For quantitative analyses, at least three independent biological replicates were used unless otherwise indicated. Exact sample sizes for each experiment are provided in the corresponding figure legends and Source Data files.

Data exclusions	No data were excluded from the analysis in this study.
Replication	Quantitative analyses were performed using independent biological replicates and analyzed statistically. Experiments were independently replicated where applicable with similar results. Exact sample sizes are provided in the figure legends and Source Data files.
Randomization	Animals or cells were randomly assigned into experimental or control groups.
Blinding	Data collection and quantification were performed by lab members blinded to mouse genotype and treatment.

Reporting for specific materials, systems and methods

We require information from authors about some types of materials, experimental systems and methods used in many studies. Here, indicate whether each material, system or method listed is relevant to your study. If you are not sure if a list item applies to your research, read the appropriate section before selecting a response.

Materials & experimental systems

n/a	Involved in the study
☐	☒ Antibodies
☒	☐ Eukaryotic cell lines
☒	☐ Palaeontology and archaeology
☐	☒ Animals and other organisms
☒	☐ Clinical data
☒	☐ Dual use research of concern
☒	☐ Plants

Methods

n/a	Involved in the study
☒	☐ ChIP-seq
☒	☐ Flow cytometry
☒	☐ MRI-based neuroimaging

Antibodies

Antibodies used	Goat polyclonal anti-Serpina3n (1:200, R&D System, Cat# AF4709; RRID:AB_2270116), Rabbit polyclonal anti-IBA1 (1:100, WAKO, Cat# 019–19741; RRID:AB_839504), Rabbit polyclonal anti-SOX10 (1:200, Abcam, Cat# ab27655; RRID: AB_778021), Mouse monoclonal anti-GFAP (1:200, or 1:20000, Agilent, Cat# Z0334; RRID:AB_10013382) , Rat monoclonal anti-CD68 (1:200, Bio-Rad, Cat# MCA1957; RRID: AB_3100585), Mouse monoclonal anti-APC (Ab-7) (CC1) (1:100, Millipore, Cat#OP80; RRID: AB_213434), Rabbit monoclonal anti-TCF4/TCF7L2 (C48H11) (1:100, Cell Signaling Technology, Cat#2569S; RRID:AB_2199816), Rabbit recombinant monoclonal anti-Stat3 (1:100, Cell Signaling, Cat# 4904, RRID:AB_331269), Rabbit recombinant monoclonal anti-pStat3 (Tyr705) (1:100, Cell Signaling, Cat# 9145; RRID:AB_2491009), Rabbit polyclonal anti-RFP (1:200, Rockland, Cat# 600-401-379; RRID:AB_2209751), Mouse monoclonal anti-Ab1-16 (1:100, clone 6E10, BioLegend, Cat# 803001; RRID:AB_2715854), Rat monoclonal anti-I-A/I-E (1:100, BD Pharmingen, Cat# 556999; RRID:AB_396545), Mouse monoclonal anti-NeuN (1:100, Millipore, Cat# MAB377; RRID:AB_2298772), Rabbit recombinant monoclonal anti-phosphor-p65 (Ser536) (1:100, Cell Signaling Technology, Cat# 3033, RRID:AB_331284), Mouse monoclonal anti-CD45 (1:100, Thermo Fisher Scientific, Cat# 14-0451-82; RRID:AB_467251), Rabbit polyclonal anti-CD74 (1:100, Thermo Fisher Scientific, Cat# PA5-22113; RRID:AB_11157006), Chicken polyclonal anti-GFP (1:200, Abcam, Cat# ab13970; RRID:AB_300798), Mouse anti-TLR4 (NOVUS, Cat# NB100; AB_2205129), Normal mouse IgG (Cell Signaling Technology, Cat# 68860; RRID:AB_3675987), Normal goat IgG (R&D Systems, Cat# AB-108-C; RRID:AB_354267).<br>All secondary antibodies used this study are from Jackson ImmunoResearch Laboratories.
Validation	All antibodies used in this study are commercially available and were validated based on manufacturer documentation for the indicated applications, previous literature using the same antibodies, expected staining or banding patterns, and/or validation experiments described in the manuscript where applicable. Supplier names, catalog numbers, clone names, lot numbers and working dilutions are provided above.

Animals and other research organisms

Policy information about studies involving animals; ARRIVE guidelines recommended for reporting animal research, and Sex and Gender in Research

Laboratory animals	All transgenic mice were maintained on a C57BL/6 background. Both male and female mice were used in this study. A total of 12 transgenic lines were used in the study, including Serpina3n-tdTom reporter line we generated by our own group, Plp-CreERT2 (RRID:IMSR_JAX:005975), Aldh1l1-CreERT2 (RRID:IMSR_JAX:031008), Aldh1l1-eGFP (RRID:IMSR_JAX:030247), RiboTag (RRID:IMSR_JAX:029977), Olig2-Cre (RRID:IMSR_JAX:025567), Serpina3n-floxed (RRID:IMSR_JAX:027511), Stat3-floxed (RRID:IMSR_JAX:016923), Rosa26-eGFP-DTA (RRID:IMSR_JAX:032087), 5xFAD (RRID:MMRRC_034848-JAX), Cx3cr1-GFP/Ccr2-RFP (RRID:IMSR_JAX:032127), and Cnp-Cre:R26StopFLikk2ca (referred to as NFkB CA mice) was from our previous study.<br><br>For MOG/EAE mice model, 12–15-week-old mice were used. For Alzheimer's disease mouse model, 7-8 months old hemizygous 5XFAD transgenic mice and age-matched wild-type mice were used. For normal aging model, 2 months old young control mice and 20 months old aged wild-type or Serpina3n cKO mice were used. All other mice are around 8 weeks old.<br><br>For primary microglia culture, P2 wild-type neonatal pups were used.

Wild animals	N/A
Reporting on sex	Both male and female mice were used; however, the study was not designed or powered to assess sex-dependent effects, and therefore sex-based analyses were not performed. For P2 neonatal pups primary culture, sex was not determined at the time of primary cell isolation, and sex-based analyses were not performed.
Field-collected samples	No field-collected samples were used in this study.
Ethics oversight	All procedures for animal surgery and maintenance were performed using protocols that were approved by the institutional Animal Care and Use Committee of of the University of of California, Davis.

Note that full information on the approval of the study protocol must also be provided in the manuscript.

Plants

Seed stocks	N/A
Novel plant genotypes	N/A
Authentication	N/A
