# Supplementary material for: Injury-transduced oligodendrocytes modulate neuroinflammation and glial activation in diseased and non-diseased central nervous system
Source: Nat Commun. 2026 Aug 29;17:10347. doi: 10.1038/s41467-026-76159-2 (PMC13623841; doi:10.1038/s41467-026-76159-2)
Supplement: Supplementary file 2 [file 41467_2026_76159_MOESM16_ESM.pdf]

nature portfolio

Peer Review File

Injury-transduced oligodendrocytes modulate neuroinflammation and glial activation in diseased and non-diseased central nervous system

Corresponding Author: Dr Fuzheng Guo

This file contains all reviewer reports in order by version, followed by all author rebuttals in order by version.

Version 0:

Reviewer comments:

Reviewer #1

(Remarks to the Author)
The authors studied a new subtype of oligodendrocytes, the SerpinOLs, showing an increase of the SERPINA3N marker in mature oligodendrocytes in several CNS injuries/pathologies such as demyelination, the ischemic stroke, the neuroinflammation induced by LPS, the neurodegeneration in the 5xFAD Alzheimer's disease model, and the normal aging. The use of several models is comprehensive and reinforces the authors' demonstration of the transition of oligodendrocytes into SerpinOLs is a common mechanism shared by multiple injury/disease conditions and aging. The use of multiple genetic and chemical-induced mouse models as well as cutting-edge techniques such as RNA-seq, and RiboRag RNA immunoprecipitation are also an asset for this article. The results put forward in this article are also innovative, since they overturn the paradigm suggesting that SERPINA3N is only a marker found in astrocytes, since the authors show that this marker mainly comes from oligodendrocytes. Also, this article highlights the function of oligodendrocytes in the promotion of neuroinflammation and aging. However, there are several areas for improvement. This is a very dense article presenting many results, unfortunately the large number of figures and conditions/models does not help the comprehension and fluidity of the reading of the manuscript. More importantly, the lack of evidence in preventing or attenuating the demyelination in the absence of SERPINA3N in mature oligodendrocytes. In addition, the lack of animal controls for several injuries or verification of the presence of a real phenotype such as verification of LPS-induced neuroinflammation after one day of treatment or the lack of evidence of demyelination in the CPZ model reduces confidence in some results. Although the authors have suggested and shown some results in favor of a relationship between SERPINA3N and STAT3 and the involvement of SerPINA3N in the promotion of a pro-inflammatory phenotype in CNS pathologies, the molecular mechanisms associated with SERPINA3N remain to be investigated. The relationship between SERPINA3N and STAT3 and other targets could be further investigated.
Other comments:
• In Figure 3F and supplementary Figure 7A, one potential issue is that the staining of SERPINA3N may not be all real because amyloid plaques are sticky and easy to bind to SERPINA3N. It is worth demonstrating that the sequential staining of SERPINA3N and 6E10, instead of co-staining. Also, is there any difference in the expression of SERPINA3N between gray matter and white matter, proximal or distal from amyloid plaques?
• Olgio-2 and CC-1 may not be specific for mature oligodendrocytes. The combination of PDGFR-alpha and MBP should be more specific for mature oligodendrocytes, instead of OPC or premyelinating oligodendrocytes.
• The authors mentioned the artifacts of co-staining of SERPINA3N and GFAP. However, have the authors considered the sequential staining of GFAP and SERPINA3N or vice versa?
• In general, the number of cells counted, the number of regions of interest and the number of animals used for each graph is missing. Please add this information to all the figures.
• In lines 26-27 on page 3, the authors state that "All cells in the SERPINA3N+ oligodendroglial line were mature CC1+ LOs but not NG2+ OPCs" but most IFs and quantifications were performed with the SOX10 marker. Even if the authors showed CC1 IF sometimes, it would have been preferable to use CC1 instead of SOX10 to remain consistent following this statement. Can the authors justify this choice?
• The authors studied the transition of homeostatic LOs to SerpinOLs in several CNS pathologies such as the ischemic stroke, neuroinflammation induced by LPS, the neurodegeneration in the Alzheimer's disease mice model and in normal aging. This multi-pathological approach is very interesting. However, the lack of control animals for the ischemic stroke and the LPS models is a weak point in this demonstration. It would be preferable to add controls to reinforce these results.

• In Figure 3, can the authors explain the choice of regions for the LPS model (cortex and subcortical white matter)? Also, no demonstration has been made by the authors to prove LPS-induced neuroinflammation in the CNS. As described in the previous comment, it would be judicious to add control animals and prove that neuroinflammation was indeed induced by LPS in the chosen regions to reinforce these results.
• On lines 2-3 page 6, the authors state "In contrast, less than 10% were GFAP+ reactive astrocytes (Fig. 3 A2-A3, Fig. 3 B2-B3, Suppl Fig. 5 C1-D2) and none were CD68+ myeloid cells (Suppl Fig. 5 E) throughout the duration of the disease". But Suppl Fig. 5 E shows "fluorescent IHC showing CD68+ myeloid cells engulfing extracellular SERPINA3N", and if this phenomenon is not always observed after injury, the authors shouldn't be so categorical. Moreover, it would be interesting to know whether this phenomenon is also observed in the LPS model.
• In the ischemic stroke panel, the authors showed several immunofluorescences for GFAP, CD68, NeuN and SERPINA3N for day 1, 2, 7 and 14. The authors should add low magnification for all the timepoints for SOX10 too, especially because it is one of the markers showing a new type SERPINA3N. Also, it would also be interesting to measure the number of cells in the lesion or penumbra for each marker to highlight glial cell recruitment, for example. Also, the numerous IFs and graphs do not make it easy to understand all the data. It could be interesting to combine the histograms into one, or to modify their representation into curves, for example.
• For most of quantifications, the authors measured the percentage of SERPINA3N cells positive for GFAP, SOX10, CD68 and "other" positive cells. Can the authors explain how they quantified the percentage of SERPINA3N cells for these different markers? Did they perform multiple IFs in the same section? The authors should add the NeuN marker especially for the normal aging model and show the percentage of SERPINA3N-positive cells in neurons too.
• For Figure 6H, the authors stated lines 16-17 page 9: "We found that approximately 20% of SOX10+ oligodendrocytes were transformed into tdTom+ SerpinOLs in the corpus callosum at this stage" but no quantification is showed. The authors should add this quantification as a graph and should apply this same quantification (% of SerpinOLs of SOX10-posiive cells) for the CPZ 4 weeks timepoint to see if there is any increase at 4 weeks compared to 2 days and see if the proportion of SerpinOLs is really related to the demyelination phenomenon. Furthermore, the authors concluded at lines 19-20 page 9 "these results suggest that oligodendroglial injury alone is sufficient to drive the transition from homeostatic to Serpin OLs". There is no evidence of the demyelination or oligodendroglial injury shown in the manuscript for the CPZ model, particularly after 2 days. The authors should add further results focusing on myelinated oligodendrocytes to show demyelination; or change this conclusion to avoid speculation.
• For some quantification, the authors counted the percentage of SERPINA3N cells in the SOX10-positive cells. In my point of view, to have this information for all the panels that showed that SERPINA3N was mostly in SOX10-positive cells is important to have an idea if this proportion represents most of the oligodendrocytes.
• The authors showed that it could exist a relationship between SERPINA3N and STAT3 by using a Stat3 cKO mice model. As the authors also used a Serpina3n cKO mice model. Did they check the expression and the localization of STAT3 in the cKO model? These results could improve this demonstration.
• The authors should correct the repetition in lines 7-8 page 10: "Unlike the spinal cord, OL loss was evident in the corpus callosum (Fig. 8I-J) in the corpus callosum, accompanied by strong activation of microglia and astrocytes (Fig. 8 F, G, H)".

Reviewer #2

(Remarks to the Author)
I co-reviewed this manuscript with one of the reviewers who provided the listed reports. This is part of the Nature Communications initiative to facilitate training in peer review and to provide appropriate recognition for Early Career Researchers who co-review manuscripts.

Reviewer #3

(Remarks to the Author)
In the manuscript "Injury-transduced SerpinOLs control neuroinflammation and glial activation in the diseased and non-diseased CNS" Wang et al. present evidence that Serpina3 is predominantly produced by mature oligodendrocytes (OLs) in response to demyelinating injury, including EAE and cuprizone (CPZ) models. Using multiple lines of evidence—RNA-seq, RT-qPCR, western blotting, immunohistochemistry, and a Serpina3n-TdTomato reporter—they convincingly show that Serpina3n expression is restricted to mature OLs, both in lesional and non-lesional areas, and across acute and chronic phases of disease.

The use of Olig2Cre:Serpina3nf/f mice provides strong support for the oligodendrocyte-specific origin of Serpina3n. Importantly, the authors extend their findings to several additional models of white matter injury (e.g., ischemic stroke, 5xFAD, spinal cord injury, aging), observing that 70–90% of Serpina3n is produced by OLs in each context. Interestingly inflammation alone does not trigger the transition of OLs into Serpina3n-expressing OLs (SerpinOLs), rather, injury or damage is required. This transition is detected as early as two days into the CPZ diet and within 24 hours of PLP-DTA induction, even prior to microglia or astrocyte activation. These data suggest a damage-sensing intrinsic response in OLs.

1. First, a point of clarification is needed regarding previous work by the same authors (Meina Zhu et al., 2024), in which they reported that Serpina3n is primarily produced in the liver and brain under homeostatic conditions and upregulated in response to systemic inflammation. In contrast, the current manuscript emphasizes the requirement for damage or injury to induce SerpinOLs. This discrepancy should be addressed.

2. The finding that Serpina3n expression is dependent on injury, disease or in the aged brain is not sufficient to justify publication. The MAJOR FLAW in this study is that it is unclear what the impact of Serpina3n expression has on nervous

system repair or maintenance, i.e. demyelination, remyelination, degeneration, microglial phagocytosis, etc. The title reads "control" neuroinflammation—if anything, Serpina3n exacerbates inflammation and does not control it. Since Serpina3n expression is dependent on injured OLs and enhances inflammation on a transcriptional level, Serpina3n is neither necessary nor sufficient in promoting inflammation. Without functional evaluation, nothing can be concluded.
If the authors want to make claims about necessity of Serpina3n in multiple animal models, they must show:
a) EAE clinical scores and histology
b) Cuprizone remyelination effects
c) Aging—Impact on neurodegeneration and loss of myelin
d) 5XFAD—cognitive function, degeneration, plaques, etc.
e) Ischemia—recovery, degeneration,
f) Spinal cord injury—regeneration, remyelination
This will greatly contribute to whether Serpina3n is necessary for recovery— is beneficial or deleterious in enhancing inflammation after injury, disease or in the aged brain. It would benefit the authors to focus on a couple of models and dig deep into the functional consequence of Serpina3n deletion from OLs.

3. Authors propose that mature oligodendrocytes are the primary responders to injury through the production of SerpinA3, which subsequently modulates microglial activity. This interpretation contrasts with a substantial evidence in demyelinating diseases, where microglia are consistently reported as the first cells to become activated—often preceding demyelination. Sustained activation of microglia can drive a neurotoxic profile characterized by the release of pro-inflammatory cytokines (e.g., IL-1β, TNF-α). The authors claim that microglial responses are governed by Serpin-OL represents a notable departure from the prevailing view of microglia as autonomous and rapid responders to CNS damage. However, this conclusion appears to be based solely on transcriptomic data. To strengthen this claim, functional validation is essential. It would be important to assess whether modulation of Serpina3n affects microglial activity and remyelination dynamics in established models such as the cuprizone paradigm. Additionally, in the context of neurodegeneration, microglia is involved in phagocytosis of pathological proteins and damaged synapses, debris clearance. In the RNA-seq analysis of microglia (cKO_MOG vs the Ctrl_MOG mice), authors found that 44% of the DEGs were significantly downregulated in the cKO. These genes were primarily associated with inflammatory/immune responses and phagocytosis. Alterated transcription was also found in the CPZ model. Since microglia have been shown to carry out a wide range of functions critical to both the degenerative and reparative phases of disease, including debris clearance, secretion of trophic factors, and promotion of remyelination. If Serpina3n deletion dampens microglial activation, my hypothesis would be that the outcome would not be beneficial. I would anticipate the opposite, it could impair endogenous recovery mechanisms.

Although the study provides extensive transcriptional evidence of Serpina3n expression in oligodendrocytes, it lacks direct functional validation of its role in modulating microglial activation. As such, the mechanistic link remains speculative. Additional evidence is required to support the proposed model, and in its current form, the manuscript is incomplete and not suitable for publication.

Reviewer #4

(Remarks to the Author)
In this study Wang and colleagues investigate an oligodendrocyte subpopulation, characterized by the upregulation of serpina3n, herein termed SerpinOLs, that was previously demostrated in various studies as a disease-associated subpopulation in several conditions in mice and human brain samples. Importantly, Serpina3n was also previously reported to be expressed in disease-associated astrocytes. This study further extend these findings, it performes a comprehensive analysis of SerpinOLs in additional conditions. The relevance and novelty of this study relys on the investigation of the molecular mechanisms and the function of serpinOLs. Authors show that serpina3n are mainly expressed by oligodendrocytes, and SerpinOLs are driven by direct oligodendrocyte injury, not inflammation. STAT3 activation seems to be critical for the expression of serpina3n in oligodendrocytes. Functionally, authors show that SerpinOLs-derived SERPINA3N amplifies pro-inflammatory responses and microglial activation in the context of disease or injury, suggesting that oligodendrocytes contribute to CNS pathology beyond myelination.

This is a timely and relevant study that advances our understanding on the serpinOLs functions and mechanisms of action across various disease and injury contexts, and it highlights a potential new target for immune modulation.

Questions and comments:

- In general quantifications in control conditions are missing in the Figs data. According to RNA there is expression of Serpina3n in OL and astrocytes (neurons were not checked? Also reported to expressed serpina3n). Did authors estimate SERPINA3N+ cells in control conditions? It is important to determine if the results represent an increase in number of SERPINA3N+OLs or increased protein expression and secretion. At least quantifications such as Fig 1H and 1R should have % expression in control, if any, or reporte if SERPINA3N+ cells are detected at baseline.
- In general, info on which spinal cord region(s) were analysed for quantifications and imaging is missing.
- Quantifications are needed to sustain this sentence: "We found that SerpinOLs were markedly increased in the regions of corpus callosum (Fig. 4G) and fimbria (Fig. 4H)". What were the basal levels?
- Figure 4G does not sustain this sentence: "The proximity of SerpinOLs with activated microglia (Fig. 4G-H, arrowheads) indicates that SerpinOLs may communicate with microglia through SERPINA3N"
- Supp Fig 5 shows that the highest number of cells expressing SERPINA3N+ in lesion at day 1-2, are neurons, which decreased sharply at day 7 and 14, with the rise of serpinOLs. This is a very interesting finding as it highlights a dynamic

temporal expression of SERPINA3N in other cell types, it is not referred to in the text. The region analysed should also be specified.
- Supp A4-5: should keep consistency with similar plots and replace cell types by markers.
- Authors should be consistent in CD68+ cells, sometimes are called microglia other times myeloid cells.
- Page 10, line 7-8, text has wrong fig legends, where it says fig 8 should be Fig7
- Did authors checked if serpinOLs are dying? For instance in the PlpCre/DTA model? In general, in other models are serpinOL surviving OL? Was any Casp3 or Tunel staining performed in serpinOLs?
- Single cell studies: dotplot or violin plot with cell type markers should be represented intead of image in Fig supp 12A. The fibroblast population is not part of CNS, should be stated their putative origin and the markers. How were the populations of activated cells assigned? Did authors have the markers and the refrence papers? Were OL from Fig Supp 12A correlated with the OL previously described in literature?
- Fig 8C also shows clear expression in astrocytes, check legend.
- In Supp 13 authors conclude that serpinOL do not express MHC-II. Although panel F conclusion was “triple IHC showing that tdTom+SOX10+ SerpinOLs (arrowheads) do not express I-A/I-E in Serpina3n-tdTom reporter mice”, one arrowhead points to a cell that has I-A/I-E green staining. As previously reported in the literature, this is a very low-abundance population that can be easily overlooked.
- Fig 8: were quantifications of double positive STAT3/SERPINA3N performed? Images of the double stainings would strenght the conclusions.
- The scoring plot for EAE in the three conditions (Serpina3n Ctrl_CFA, Serpina3n Ctrl_MOG and Serpina3n cKO_MOG) show be shown. Was there any improvement or exhacerbation of EAE scoring in cKO? This is important to correlate with article showing and improvement in EAE scoring after SERPINA3N injection despite no observed reduction the number of infiltrating immune cells.

The question remains, are serpinOL protected or dying? Is OL-derived SERPINA3N detrimental or protective for disease establisment and or progression? This study would benefit from additional analysis on one models performed, for intance, analysis of serpinOL for apoptosis markers, lesion size and/or OL/myelin loss in cKO.

Version 1:

Reviewer comments:

Reviewer #1

(Remarks to the Author)
The authors have addressed the concerns raised in the previous review. The additional experiments strengthen the study's conclusions and improve the quality of the manuscript. The reorganization of the manuscript to highlight key data from demyelination models and normal aging models in the main figures is greatly appreciated and makes for easier reading. The addition of control animals helps to convince the reader of the essential role of SERPINA3. In the legends, details regarding the quantifications performed or the number of animals used improve the quality of the article. Thus, I would like to recommend accepting this manuscript for publication.

Reviewer #2

(Remarks to the Author)
I co-reviewed this manuscript with one of the reviewers who provided the listed reports. This is part of the Nature Communications initiative to facilitate training in peer review and to provide appropriate recognition for Early Career Researchers who co-review manuscripts.

Reviewer #3

(Remarks to the Author)
While the authors provide evidence for an increased number of mature oligodendrocytes in the Serpina3n cKO condition, this does not address the unresolved issue from my prior comments. The manuscript does not establish a causal, mechanistic link detailing how Serpina3N+ oligodendrocytes controls microglial functional states (microglia-intrinsic vs indirect effects) to produce these outcomes. The association between Serpina3N expression and microglial changes across the different models remains descriptive. While they speculate Serpina3N may drive the microglial switch toward a pro-survival phenotype, the current data do not convincingly demonstrate this switch. Evidence that Serpina3N acts directly on microglia is required to substantiate the proposed pro-survival/remyelination effects..

Reviewer #4

(Remarks to the Author)
The authors have addressed my all questions and concerns. Congratulations for this significant work.

Version 2:

Reviewer comments:

Reviewer #3

(Remarks to the Author)
The authors should be commended for their revisions. The new data demonstrates the interaction of SERPINA3N with microglia and the interaction with TLR4.

Open Access This Peer Review File is licensed under a Creative Commons Attribution 4.0 International License, which permits use, sharing, adaptation, distribution and reproduction in any medium or format, as long as you give appropriate credit to the original author(s) and the source, provide a link to the Creative Commons license, and indicate if changes were made.
In cases where reviewers are anonymous, credit should be given to 'Anonymous Referee' and the source.
The images or other third party material in this Peer Review File are included in the article's Creative Commons license, unless indicated otherwise in a credit line to the material. If material is not included in the article's Creative Commons license and your intended use is not permitted by statutory regulation or exceeds the permitted use, you will need to obtain permission directly from the copyright holder.
To view a copy of this license, visit https://creativecommons.org/licenses/by/4.0/

Please see the point-by-point responses to reviewers' comments below.

Reviewer #1 (Remarks to the Author):

The authors studied a new subtype of oligodendrocytes, the SerpinOLs, showing an increase of the SERPINA3N marker in mature oligodendrocytes in several CNS injuries/pathologies such as demyelination, the ischemic stroke, the neuroinflammation induced by LPS, the neurodegeneration in the 5xFAD Alzheimer's disease model, and the normal aging. The use of several models is comprehensive and reinforces the authors' demonstration of the transition of oligodendrocytes into SerpinOLs is a common mechanism shared by multiple injury/disease conditions and aging. The use of multiple genetic and chemical-induced mouse models as well as cutting-edge techniques such as RNA-seq, and RiboRag RNA immunoprecipitation are also an asset for this article. The results put forward in this article are also innovative, since they overturn the paradigm suggesting that SERPINA3N is only a marker found in astrocytes, since the authors show that this marker mainly comes from oligodendrocytes. Also, this article highlights the function of oligodendrocytes in the promotion of neuroinflammation and aging. However, there are several areas for improvement.

Response: We thank the reviewer for appreciating the significance and innovation of the study. We revised the manuscript according to your insightful comments.

1). This is a very dense article presenting many results, unfortunately the large number of figures and conditions/models does not help the comprehension and fluidity of the reading of the manuscript.

Response: We truly thank the reviewer for this insightful critique of improving our manuscript. To increase the readability and fluidity of the manuscript, we have re-organized the figures by retaining the key data of demyelinating models and normal aging models in the main figures, as they directly relate to the central theme of the current study - injury-transduced SerpinOLs modulate neuroinflammation and glial activation in the diseased (demyelinating) and non-diseased (normal aging) CNS.

The major purpose of using other mouse models (stroke, LPS, 5xFAD, SCI) is to "reinforce the demonstrate of the transition of oligodendrocytes into SerpinOLs is a common mechanism shared by multiple injury/disease conditions" as pointed out by the reviewer. Therefore, the data of Serpina3n expression in these models have been moved to Supplementary Figures (Suppl Figs. 5-9) to streamline the presentation while ensuring that all supporting data remain accessible to readers. We also shortened the description of SERPINA3N expression in these injury models (see the Results section). This reorganization would substantially help the comprehension and fluidity of the manuscript without impacting data integrity and availability.

2). the lack of evidence in preventing or attenuating the demyelination in the absence of SERPINA3N in mature oligodendrocytes.

Response: We appreciate the reviewer's critique. We conducted further analyses and presented new data showing that Serpina3n cKO attenuated demyelination and promoted remyelination (Please see Fig. S16, Fig. S17, Fig. S19). Together, these findings provide evidence that the absence of SERPINA3N in oligodendrocytes mitigates demyelination and supports remyelination in the diseased (demyelinating) and non-diseased (normal aging) CNS.

3). In addition, the lack of animal controls for several injuries or verification of the presence of a real phenotype such as verification of LPS-induced neuroinflammation after one day of treatment or the lack of evidence of demyelination in the CPZ model reduces confidence in some results.

Response: Great point! In the revised manuscript, we have added appropriate controls for all disease/injury models, showing that Serpina3n expression is minimal in control mice compared with those with CNS diseases/injuries (please see Fig. 1h; Fig. 1p; Fig. S5a; Fig. S6e, k; Fig. S9b).

We have also included new data confirming LPS-induced neuroinflammation (Fig. S6a-d), CPZ-induced demyelination (Fig. S16f), and EAE-induced demyelination (Fig. S16d). These new data strengthen the validation of our models and the reliability of our results.

4). Although the authors have suggested and shown some results in favor of a relationship between SERPINA3N and STAT3 and the involvement of SerPINA3N in the promotion of a pro-inflammatory phenotype in CNS pathologies, the molecular mechanisms associated with SERPINA3N remain to be investigated. The relationship between SERPINA3N and STAT3 and other targets could be further investigated.

Response: We appreciate the reviewer's insightful comment and agree that the relationship between SERPINA3N and STAT3 warrants further investigation. In the original submission, we identified STAT3 as an upstream factor activating SERPINA3N expression in OLs (Fig. 7k-m). In the revised manuscript, we provided further insights into the regulation and demonstrated that STAT3 may regulate SERPINA3N by directly binding to the promoter regions and activating Serpoina3n transcription (Fig. 7o). Furthermore, SERPINA3N depletion reduced CNS inflammation (Fig. 8) and demyelination (Fig. S16) in demyelinating mouse animals, which may, in turn, dampen the activation of STAT3 in oligodendroglial cells (Fig. S12), a major signaling pathway in regulating neuroinflammation. Together, our data suggest a reciprocal regulation of STAT3 and SERPINA3N in OLs.

Other comments:

5) In Figure 3F and supplementary Figure 7A, one potential issue is that the staining of SERPINA3N may not be all real because amyloid plaques are sticky and easy to bind to SERPINA3N. It is worth demonstrating that the sequential staining of SERPINA3N and 6E10, instead of co-staining.
Also, is there any difference in the expression of SERPINA3N between gray matter and white matter, proximal or distal from amyloid plaques?

Response: We thank the reviewer for raising the possibility that SERPINA3N immunoreactivity in 5xFAD mice could reflect nonspecific binding to sticky amyloid plaques. We performed sequential staining for SERPINA3N and 6E10. In the subiculum (grey matter), we first stained SERPINA3N and then applied 6E10 (see Panel A of the figure below) We also performed immunostaining in an opposite order (6E10 → SERPINA3N; see Panel B of the figure below). In both staining sequences, extracellular SERPINA3N signal remained stably detectable and consistently co-localized with $6E10^{+}$ Aβ plaques. This order-independent co-localization supports the interpretation that SERPINA3N is genuinely deposited within and around amyloid plaques. Our observation (SERPINA3N colocalization with amyloid plaques) is also consistent with a previous study in 1988 (PMID: 3257719).

We examined regional differences in grey versus white matter. In the cortex, SERPINA3N and 6E10 show robust co-localization around parenchymal plaques, indicating that plaque-rich grey matter is a major site of extracellular SERPINA3N accumulation (see Panel C of the figure below). In contrast, 6E10 staining shows few or no Aβ plaques in the corpus callosum (white matter), yet SERPINA3N immunoreactivity is still present (see Panel D of the figure below), suggesting that SERPINA3N induction can occur in plaque-poor white matter, presumably due to oligodendrocyte injury in 5xFAD model.

Taken together, the sequential staining experiments demonstrate that SERPINA3N co-localization with 6E10$^+$ plaques in the grey matter is unlikely a technical artifact and that SERPINA3N is induced in both plaque-rich grey matter and plaque-poor white matter.

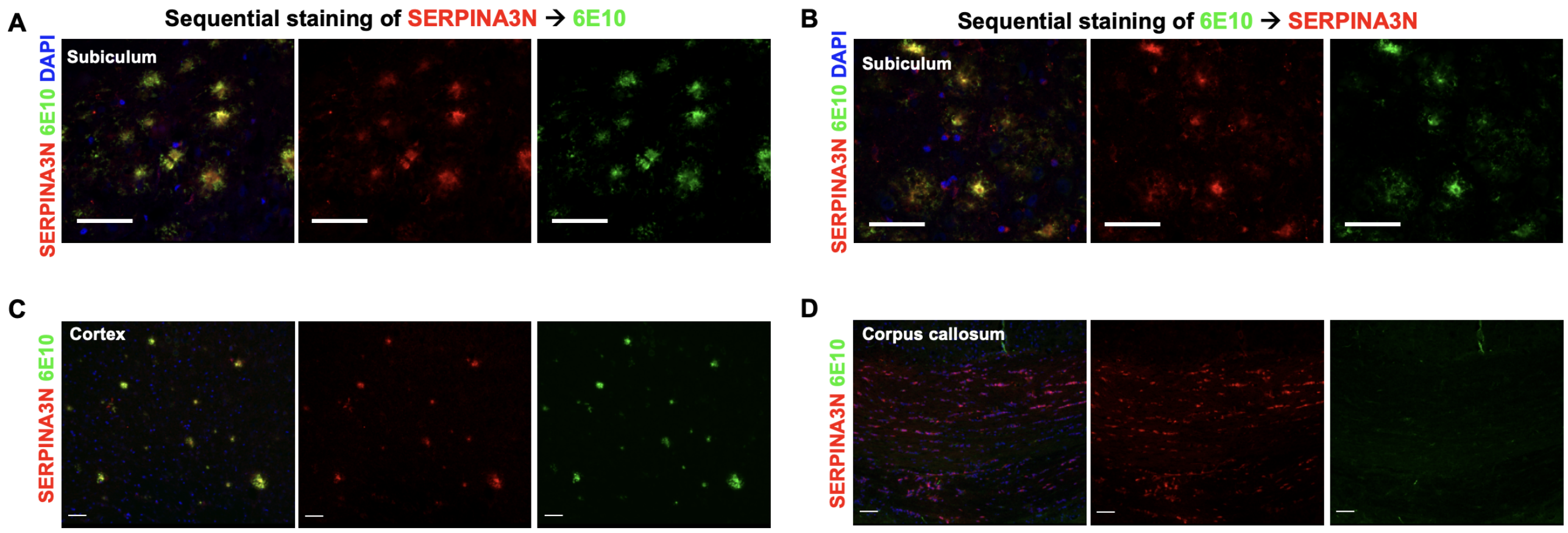

6) Olgio-2 and CC-1 may not be specific for mature oligodendrocytes. The combination of PDGFR-alpha and MBP should be more specific for mature oligodendrocytes, instead of OPC or premyelinating oligodendrocytes.

Response: Thanks for this comment. We agree with the reviewer that Olig2 is not specific to mature OLs as it also labels PDGFRa$^+$ OPCs and that MBP is more specific for mature OLs. However, MBP is predominantly located in the distal processes of mature OLs and largely absence from cell bodies, making it difficult to identify cytoplasmic SERPINA3N-expressing cells (see the figure below).

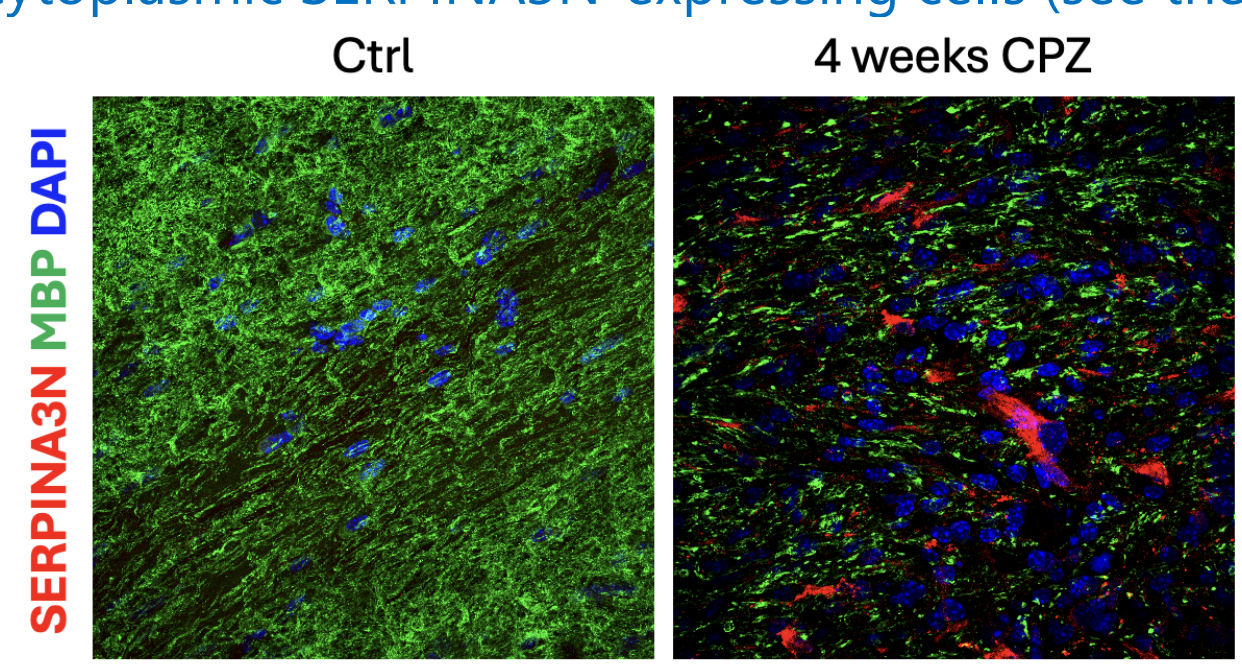

Instead, we used OPC marker NG2 to identify Serpina3n-expressing cells as non-OPCs and then used CC1 to identify them as OLs (please see Fig. 1j, t). We further employed CC1 and premyelinating OL marker TCF7l2 to identify Serpina3n-expressing OLs as mature OLs (but not TCF7l2$^+$ premyelinating OLs) (please see Fig. 1u, Fig. S2c). Together, our data demonstrate that Serpina3n-expressing cells are mature OLs.

7) The authors mentioned the artifacts of co-staining of SERPINA3N and GFAP. However, have the authors considered the sequential staining of GFAP and SERPINA3N or vice versa?

Response: Great point! We performed the sequential immunostaining in the corpus callosum of CPZ model mice (see Fig. S3g-h) and fimbria of the 5xFAD model mice (see Fig. S8f-g), in both orders (GFAP→SERPINA3N and SERPINA3N→GFAP). These experiments produced consistent results and confirmed that SERPINA3N does not extensively colocalize with GFAP$^+$ reactive astrocytes under either condition. Together, these data indicate that the extensive overlap of SERPINA3N/GFAP observed in conventional co-staining was probably an artifact, rather than true biological colocalization.

8) In general, the number of cells counted, the number of regions of interest and the number of animals used for each graph is missing. Please add this information to all the figures.

Response: We thank the reviewer for this important suggestion. In the revised manuscript, we have added the important information to figure legends.

9) In lines 26-27 on page 3, the authors state that "All cells in the SERPINA3N+ oligodendroglial line were mature CC1+ LOs but not NG2+ OPCs" but most IFs and quantifications were performed with the SOX10 marker. Even if the authors showed CC1 IF sometimes, it would have been preferable to use CC1 instead of SOX10 to remain consistent following this statement. Can the authors justify this choice?

Response: We appreciate the reviewer's insightful comment (SOX10 labels all developmental stages of oligodendroglial lineage cells). In the adult CNS, >90% of SOX10+ cells are OLs and <10% are OPCs. As described above in #6, we first spent substantial effort identifying SERPINA3N-expressing cells as $CC1^+$ mature OL ($SOX10^+$), but not $NG2^+$ OPCs ($SOX10^+$) or $TCF7l2^+$ premyelinating OLs ($SOX10^+$). Therefore, $SERPINA3N^+/SOX10^+$ oligodendroglial lineage cells are mature OLs.
CC1 is a cytoplasmic marker, and its staining intensity and pattern can vary with disease/injury state. Instead, SOX10 is a consistent nuclear transcription factor, which technically makes the quantification unambiguous and precise. We did perform SERPINA3N/CC1 quantification i.e. the percentage of $SERPINA3N^+$ cells among total OLs (please see Fig. 1k). In other circumstances, we simply quantified the percentage of $SERPINA3N^+$ cells that belong to oligodendroglial lineage (SOX10), astroglial lineage (GFAP), or activated microglia/macrophages (CD68) (for example Fig. 1i, s).

10) The authors studied the transition of homeostatic LOs to SerpinOLs in several CNS pathologies such as the ischemic stroke, neuroinflammation induced by LPS, the neurodegeneration in the Alzheimer's disease mice model and in normal aging. This multi-pathological approach is very interesting. However, the lack of control animals for the ischemic stroke and the LPS models is a weak point in this demonstration. It would be preferable to add controls to reinforce these results.

Response: We thank the reviewer for highlighting the importance of appropriate controls in the ischemic stroke and LPS models. In the revised manuscript, we have included IHC data from control animals (please see Fig. 1h, Fig. 1p, Fig. 7f, Fig. S3d, Fig. S4e, f, Fig. S5a, Fig. S6e, k, Fig. S7c, Fig. S9b). In conclusion, minimal or no SERPINA3N staining signal was observed in control animals, in line with our conclusion that SERPINA3N is an injury-induced molecule expressed in OLs.

11) In Figure 3, can the authors explain the choice of regions for the LPS model (cortex and subcortical white matter)? Also, no demonstration has been made by the authors to prove LPS-induced neuroinflammation in the CNS. As described in the previous comment, it would be judicious to add control animals and prove that neuroinflammation was indeed induced by LPS in the chosen regions to reinforce these results.

Response: We thank the reviewer for this constructive comment. Our selection of the cortex and subcortical white matter in the LPS model was guided by both biological relevance and prior studies (Zamanian et al, 2012, PMID: 22553043). Systemic LPS administration is well known to induce robust neuroinflammation in cortical regions and adjacent white-matter tracts. Zamanian et al. demonstrated strong neuroinflammation, glial activation, and induction of Serpina3n in the cortex and corpus callosum following LPS treatment.

In accordance with the reviewer's suggestion, we have strengthened the LPS data in two ways:
a) Addition of control animals: We included vehicle (PBS)-injected controls processed in parallel and analyzed at the same time point as LPS-treated animals. In these controls, SERPINA3N expression remained at baseline, with minimal or no $SERPINA3N^+$ cells or tdTomato-labeled cells in Serpina3n-tdTom mice (Fig. S6).

b) Demonstration of LPS-induced neuroinflammation in the analyzed regions: In the same cortical and subcortical white-matter areas used for SerpinOL quantification, LPS-treated mice exhibit marked increases in $Iba1^+$/$CD68^+$ activated microglia/macrophages and $GFAP^+$ reactive astrocytes, whereas saline controls show only baseline glial activation (Fig. S6).

Together, these additional controls and validation experiments confirm that LPS induces robust neuroinflammation in the chosen cortical and subcortical white-matter regions, and that the emergence of $SERPINA3N^+$ "SerpinOLs" reflects a true inflammatory CNS response rather than staining artifacts.

12) On lines 2-3 page 6, the authors state "In contrast, less than 10% were GFAP+ reactive astrocytes (Fig. 3 A2-A3, Fig. 3 B2-B3, Suppl Fig. 5 C1-D2) and none were CD68+ myeloid cells (Suppl Fig. 5 E) throughout the duration of the disease". But Suppl Fig. 5 E (stroke model) shows "fluorescent IHC showing CD68+ myeloid cells engulfing extracellular SERPINA3N", and if this phenomenon is not always observed after injury, the authors shouldn't be so categorical. Moreover, it would be interesting to know whether this phenomenon is also observed in the LPS model.

Response: We thank the reviewer for pointing out this important clarification. Our original statements were indeed too categorical since this phenomenon is not always observed after injury. We believe that such statements (microglial engulfment of extracellular SERPINA3N in stroke, 5xFAD, and SCI) diverts the readers from the central theme of the study and negatively impacts the fluidity and comprehension of the manuscript. Therefore, we deleted such statements and descriptions in the revised manuscript.

Nevertheless, to address the reviewer's concern, we observed the phenomenon (extracellular SERPINA3N engulfed by $CD68^+$ cells) more frequently in 5xFAD model (despite low absolute incidence) than SCI and stroke models, and none in LPS model. As described above, we removed such description from the revision.

13) In the ischemic stroke panel, the authors showed several immunofluorescences for GFAP, CD68, NeuN and SERPINA3N for day 1, 2, 7 and 14. The authors should add low magnification for all the timepoints for SOX10 too, especially because it is one of the markers showing a new type SERPINA3N. Also, it would also be interesting to measure the number of cells in the lesion or penumbra for each marker to highlight glial cell recruitment, for example. Also, the numerous IFs and graphs do not make it easy to understand all the data. It could be interesting to combine the histograms into one, or to modify their representation into curves, for example.

Response: Thank you for this very helpful suggestion. We apologize for the confusion of data presentation at different time points after stroke injury. To improve clarity and emphasize the central findings, we have substantially reorganized the ischemic stroke data (please see Fig. S5). In brief,

a) Streamlined time-course data: We removed the full time-course immunofluorescence panels for GFAP, CD68, and NeuN at days 1, 2, 7, and 14 post-stroke. Instead, we focused exclusively on $SERPINA3N^+$ cell counts in the lesion and penumbra at these timepoints (Fig. S5a), which directly reflect the temporal dynamics of SERPINA3N expression.
b) Presented representative images at key pathological stages only (Day 7 and Day 2). The number of $Serpina3n^+$ cells peaks at day 7 post-stroke, a time point corresponds to the stage of glial scar formation. We therefore include representative immunohistochemistry images of SERPINA3N, GFAP, SOX10, CD68, and NeuN at day 7 (Fig. S5b-e). This approach highlights cellular localization while avoiding unnecessary redundancy.
c) Combined quantification plots: To improve readability, we consolidated the quantifications of SERPINA3N and cell-type markers into curves rather than multiple separate histograms (Fig. S5g).

This presentation more clearly illustrates how SERPINA3N expression relates to cell types within the same analytical framework.

14) For most of quantifications, the authors measured the percentage of SERPINA3N cells positive for GFAP, SOX10, CD68 and “other” positive cells. Can the authors explain how they quantified the percentage of SERPINA3N cells for these different markers? Did they perform multiple IFs in the same section? The authors should add the NeuN marker especially for the normal aging model and show the percentage of SERPINA3N-positive cells in neurons too.

Response: Thank you for the comments. Please see the Materials and Methods section in the revised manuscript. Briefly,
a) Quantification method. For each model, we quantified the percentage of SERPINA3N$^+$ cells co-expressing GFAP, SOX10, CD68, or designated “Other” as SERPINA3N$^+$ cells that did not colocalize with any of these markers. Immunofluorescence staining of at least 3 sections from each animal were used for quantification. This quantification workflow has been added to the Methods section for clarity.

b) Neuronal quantification in the normal aging model. SERPINA3N$^+$ cells were observed predominantly in the subcortical white matter regions and few in the cortical areas in normal aging model. We evaluated SERPINA3N$^+$ cells in the corpus callosum (CC) and fimbria, white matter regions where NeuN$^+$ neurons are essentially absent. As illustrated in the representative image below, the CC contains no NeuN$^+$ neurons (green). In contrast, the cortex (right) displays dense NeuN$^+$ neuronal labeling but very limited SERPINA3N expression.

Since SERPINA3N and NeuN show minimal spatial overlap in the aging brain, and NeuN$^+$ neurons are absent in white-matter regions analyzed (CC and fimbria), we did not include SERPINA3N$^+$/NeuN$^+$ double-positive quantification for the aging brain.

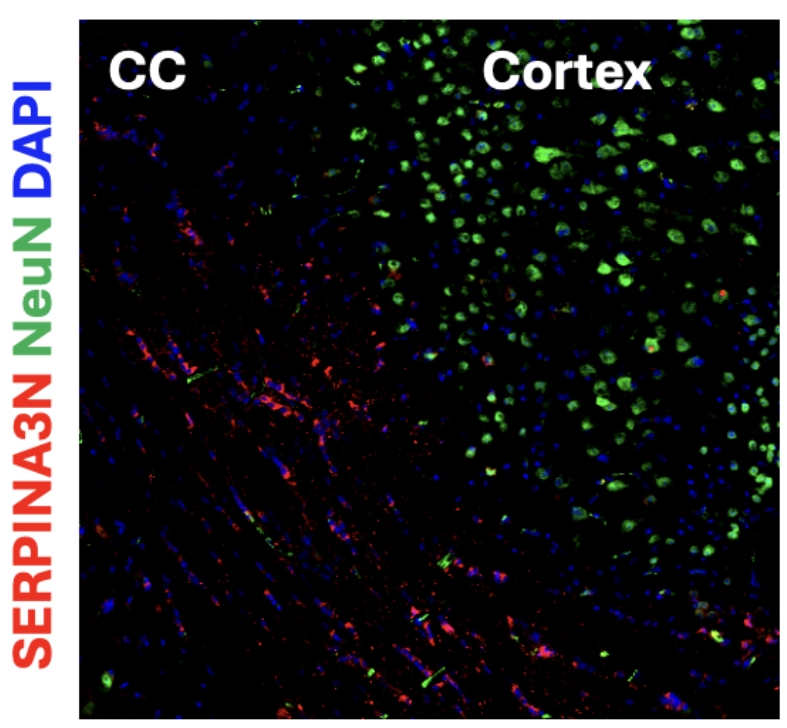

15) For Figure 6H, the authors stated lines 16-17 page 9: “We found that approximately 20% of SOX10+ oligodendrocytes were transformed into tdTom+ SerpinOLs in the corpus callosum at this stage” but no quantification is showed. The authors should add this quantification as a graph and should apply this same quantification (% of SerpinOLs of SOX10-posiive cells) for the CPZ 4 weeks timepoint to see if there is any increase at 4 weeks (Fig 2F, 2H) compared to 2 days (Fig. 6H) and see if the proportion of SerpinOLs is really related to the demyelination phenomenon.
Furthermore, the authors concluded at lines 19-20 page 9 “these results suggest that oligodendroglial injury alone is sufficient to drive the transition from homeostatic to Serpin OLs”. There is no evidence of the demyelination or oligodendroglial injury shown in the manuscript for the CPZ model, particularly after 2 days. The authors should add further results focusing on myelinated oligodendrocytes to show demyelination; or change this conclusion to avoid speculation.

Response: Thank you for these constructive comments.

Please see the revised Fig. 5j (% tdTom$^+$ OLs and astrocytes at day 2 CPZ treatment). Please also see revised Fig. 2h (% of tdTom$^+$ OLs and astrocytes at week 4 CPZ treatment).

Previous studies show that OL injury/damage occurs as early as day 1 after CPZ diet treatment (PMID: 33106352), yet demyelination occurs at much later time points (PMID: 35526004). OL injury/damage and demyelination is asynchronized in CPZ model. The increased % of tdTom$^+$ OLs suggests that SerpinOL transition is related to oligodendrocyte damage/injury (not necessarily direct demyelination). This conclusion is also supported by the data from the genetic model of OL damage/injury (see revised Fig. 6)

16) For some quantification, the authors counted the percentage of SERPINA3N cells in the SOX10-positive cells. In my point of view, to have this information for all the panels that showed that SERPINA3N was mostly in SOX10-positive cells is important to have an idea if this proportion represents most of the oligodendrocytes.

Response: Thank you for this important comment. Please see the responses to comment #9. We agree with the reviewer that SOX10 is a pan-marker for oligodendroglial lineage cells labeling OLs and OPCs. In the adult CNS, greater than 90% of SOX10-positive cells are oligodendrocytes, the remainder OPCs. The purpose of quantifying SERPINA3N$^+$ cells among the SOX10$^+$ (or GFAP$^+$, CD68$^+$) populations is to define the lineage identity of SERPINA3N-positive cells.

We first showed that the majority of SERPINA3N$^+$ cells belong to SOX10$^+$ oligodendroglial lineage. We then used mature OL marker CC1, premyelinating OL marker TCF7l2, and OPC marker NG2 to further assign SOX10$^+$/SERPINA3N$^+$ cells as oligodendrocytes (Fig. 1j, Fig. 1t, Fig. 1u, Fig. S2c).

17) The authors showed that it could exist a relationship between SERPINA3N and STAT3 by using a Stat3 cKO mice model. As the authors also used a Serpina3n cKO mice model. Did they check the expression and the localization of STAT3 in the cKO model? These results could improve this demonstration.

Response: We appreciate the reviewer's thoughtful comment. Please see the revised Fig. S12 (STAT3 quantification in Serpina3n cKO mice).

To address this point, we examined the expression level and localization of STAT3 in Serpina3n cKO mice under CFA and EAE conditions. As expected, STAT3 expression is markedly induced in the EAE spinal cord compared with CFA controls. Importantly, STAT3 signal intensity is reduced in the EAE spinal cords of Serpina3n cKO mice relative to Ctrl_MOG mice, whereas its cellular and regional distribution pattern remains unchanged (revised Fig. S12).

Please also see the responses to comment #4 regarding the relationship between STAT3 and SERPINA3N.

18) The authors should correct the repetition in lines 7-8 page 10: "Unlike the spinal cord, OL loss was evident in the corpus callosum (Fig. 8I-J) in the corpus callosum, accompanied by strong activation of microglia and astrocytes (Fig. 8 F, G, H)".

Response: Thank you for pointing out this typographical error. We have removed the repeated phrase.

Reviewer #3 (Remarks to the Author)

In the manuscript "Injury-transduced SerpinOLs control neuroinflammation and glial activation in the diseased and non-diseased CNS" Wang et al. present evidence that Serpina3 is predominantly produced by mature oligodendrocytes (OLs) in response to demyelinating injury, including EAE and cuprizone (CPZ) models. Using multiple lines of evidence—RNA-seq, RT-qPCR, western blotting, immunohistochemistry, and a Serpina3n-TdTomato reporter—they convincingly show that Serpina3n expression is restricted to mature OLs, both in lesional and non-lesional areas, and across acute and chronic phases of disease.

The use of Olig2Cre:Serpina3nf/f mice provides strong support for the oligodendrocyte-specific origin of Serpina3n. Importantly, the authors extend their findings to several additional models of white matter injury (e.g., ischemic stroke, 5xFAD, spinal cord injury, aging), observing that 70–90% of Serpina3n is produced by OLs in each context. Interestingly inflammation alone does not trigger the transition of OLs into Serpina3n-expressing OLs (SerpinOLs), rather, injury or damage is required. This transition is detected as early as two days into the CPZ diet and within 24 hours of PLP-DTA induction, even prior to microglia or astrocyte activation. These data suggest a damage-sensing intrinsic response in OLs.

1) First, a point of clarification is needed regarding previous work by the same authors (Meina Zhu et al., 2024), in which they reported that Serpina3n is primarily produced in the liver and brain under homeostatic conditions and upregulated in response to systemic inflammation. In contrast, the current manuscript emphasizes the requirement for damage or injury to induce SerpinOLs. This discrepancy should be addressed.

Response: We thank the reviewer for pointing out this seemingly discrepancy. We believe that the reviewer is referring to the work by Zhu et al. (PMID: 38647003), a review paper entitled - regulation of CNS pathology by Serpina3n/SERPINA3: the knowns and the puzzles, and Zhu et al., (PMID: 39450611), a data paper entitled - dispensable regulation of brain development and myelination by the immune-related protein Serpina3n.

This prior work does not overlap with the current study, nor does it conflict with the conclusion of the current study. In the review paper, we showed that the baseline level of liver SERPINA3N was significantly increased in the adult mice in response to LPS systemic inflammation. In the current study, we demonstrated that brain SERPINA3N was significantly increased in response to LPS systemic inflammation and identified Serpina3n-expressing cells in the brain as OLs (SerpinOLs).

In the data paper, we reported that SERPINA3N was transiently expressed in oligodendrocytes during early postnatal development. We used genetic knockout approaches and concluded that transient SERPINA3N expression does not affect oligodendrocyte differentiation, developmental myelination, or the development of other glial cells and neurons. In the current study, we found no or limited SERPINA3N in the adult homeostatic CNS (Fig. 1h, Fig. 1p) yet significantly induced in oligodendrocytes under multiple disease/injury and normal aging conditions. These data, in combination with additional key data in the manuscript (Fig. 4-5), suggest a requirement of intrinsic damage or injury in transitioning SerpinOLs, as pointed out by the reviewer.

2) The finding that Serpina3n expression is dependent on injury, disease or in the aged brain is not sufficient to justify publication. The MAJOR FLAW in this study is that it is unclear what the impact of Serpina3n expression has on nervous system repair or maintenance, i.e. demyelination, remyelination, degeneration, microglial phagocytosis, etc. The title reads "control" neuroinflammation—if anything, Serpina3n exacerbates inflammation and does not control it. Since Serpina3n expression is dependent on injured OLs and enhances inflammation on a transcriptional level, Serpina3n is neither necessary nor sufficient in promoting inflammation. Without functional evaluation, nothing can be concluded.
If the authors want to make claims about necessity of Serpina3n in multiple animal models, they must show:

a) EAE clinical scores and histology
b) Cuprizone remyelination effects
c) Aging—Impact on neurodegeneration and loss of myelin
d) 5XFAD—cognitive function, degeneration, plaques, etc.
e) Ischemia—recovery, degeneration,
f) Spinal cord injury—regeneration, remyelination
This will greatly contribute to whether Serpina3n is necessary for recovery— is beneficial or deleterious in enhancing inflammation after injury, disease or in the aged brain. It would benefit the authors to focus on a couple of models and dig deep into the functional consequence of Serpina3n deletion from OLs.

Response: We appreciate the reviewer's constructive comments and great points! We have added new functional data in the revised manuscript, strengthening the impact of oligodendroglial SERPINA3N on neuroinflammation, glial activation, demyelination, remyelination, and neurodegeneration.

We fully agree with the reviewer that "the finding that Serpina3n expression is dependent on injury, disease or in the aged brain is not sufficient to justify publication". As pointed out by Reviewer #1 (summary statement, also see responses to Rev #1 Comment #1), the use of several disease/injury models is to demonstrate that the transition of oligodendrocytes into SerpinOLs is a common mechanism shared by multiple injury/disease conditions and aging. This part of study challenged the previous dogma that SERPINA3N is expressed in astrocytes under LPS model and other disease/injury models. Instead, we showed that OLs are the major cell types that express SERPINA3N under diseased/injured conditions.

Beyond Serpina3n expression, we studied the mechanisms underlying SerpinOL transition (Fig. 4-6), an open question in the field (PMID: 38513664, PMID: 39322475). We demonstrated that intrinsic OL damage/injury, rather than neuroinflammation which is a common feature of CNS diseases/injuries, transitions homeostatic OLs into SerpinOLs. Our functional data further showed that depleting SERPINA3N attenuated neuroinflammation and glial activation in the diseased (demyelinating) and non-diseased (normal aging) CNS.

As Reviewer #4 noted, "Functionally, authors show that SerpinOL-derived SERPINA3N amplifies pro-inflammatory responses and microglial activation in the context of disease or injury." In the revised study, we provided additional data delineating the functional consequence of Serpina3n deletion from OLs focusing on the diseased models (EAE and CPZ) and non-diseased model (normal aging):

a) In EAE injury model, Serpina3n cKO reduced inflammatory responses (Fig. 8 a-i), demyelination (Fig. S16 d) and improved motor functions of EAE animals (Fig. S16 a-c).
b) In CPZ demyelination model, Serpina3n cKO attenuated neuroinflammation and glial activation (Fig. 8 j-s), oligodendrocyte loss (Fig. S16 e), and demyelination (Fig. S16 f).
c) In CPZ remyelination model, Serpina3n cKO favored oligodendrocyte regeneration and enhanced remyelination (Fig. S17).
d) In normal aging model, Serpina3n cKO decreased neuroinflammation and glial responses (Fig. 9) and attenuated aging-elicited myelin damage (Fig. S19 b-d), axonal dysfunction (Fig. S19 a), and oxidative stress (Fig. S19 e).

Taken together, these functional data provide extensive evidence that oligodendrocyte-derived SERPINA3N exacerbates disease pathologies including neuroinflammation, demyelination, and/or axonal dysfunction. We have revised the Results and Discussion to reflect the major conclusion that OL-derived Serpina3n acts as a pathogenic amplifier of CNS pathologies under diseased and non-diseased conditions.

3-1) Authors propose that mature oligodendrocytes are the primary responders to injury through the production of SerpinA3, which subsequently modulates microglial activity. This interpretation contrasts with a

substantial evidence in demyelinating diseases, where microglia are consistently reported as the first cells to become activated—often preceding demyelination. Sustained activation of microglia can drive a neurotoxic profile characterized by the release of pro-inflammatory cytokines (e.g., IL-1β, TNF-α). The authors claim that microglial responses are governed by Serpin-OL represents a notable departure from the prevailing view of microglia as autonomous and rapid responders to CNS damage. However, this conclusion appears to be based solely on transcriptomic data. To strengthen this claim, functional validation is essential.

Response: We thank the reviewer for these thoughtful comments.
We agree with the reviewer that microglia are usually the first responders to become activated – often preceding demyelination. In CPZ demyelination model, CPZ diet induces rapid OL injury/damage as early as day 1 (PMID: 33106352) when microglial activation and demyelination are yet to observe (PMID: 35526004). Our data showed that OLs respond to CPZ diet by upregulating SERPINA3N on day 2 after treatment, a time point when no evidence of microglial activation is observed (Fig. 5). In the manuscript, we proposed (and demonstrated) that injury-transduced SerpinOLs amplify neuroinflammation and microglial activation.

We also agree with the reviewer that activated microglia release pro-inflammatory cytokines, which may further aggravate OL injury and SERPINA3N expression. To address reviewer's critiques and to supplement our transcriptomic data, we added new validation data showing that Serpina3n cKO significantly diminishes injury-elicited elevation in astroglial and microglial activation in EAE model (see Fig. 8d, e), CPZ model (see Fig. 8r), and normal aging model (Fig. 9d).

Accordingly, we have revised the manuscript to avoid implying that oligodendrocytes "replace" microglia as primary initiators of neuroinflammation. Instead, our data support a working model in which injury-transduced oligodendrocytes, through induction of SERPINA3N, amplify neuroinflammation and glial activation (see Figs. 8-9).

3-2) It would be important to assess whether modulation of Serpina3n affects microglial activity and remyelination dynamics in established models such as the cuprizone paradigm. Additionally, in the context of neurodegeneration, microglia is involved in phagocytosis of pathological proteins and damaged synapses, debris clearance. In the RNA-seq analysis of microglia (cKO_MOG vs the Ctrl_MOG mice), authors found that 44% of the DEGs were significantly downregulated in the cKO. These genes were primarily associated with inflammatory/immune responses and phagocytosis. Altered transcription was also found in the CPZ model. Since microglia have been shown to carry out a wide range of functions critical to both the degenerative and reparative phases of disease, including debris clearance, secretion of trophic factors, and promotion of remyelination. If Serpina3n deletion dampens microglial activation, my hypothesis would be that the outcome would not be beneficial. I would anticipate the opposite, it could impair endogenous recovery mechanisms.

Response: These are great points! We showed that Serpina3n cKO diminishes activation and increases homeostasis of microglia (please see Fig. 8 p, q, r), attenuates demyelination (Fig. S16 f), and promotes remyelination (Fig. S17) in the cuprizone paradigm.

We agree with the reviewer that microglial activation can have both degenerative and reparative effects. Our in vitro data showed SERPINA3N increases microglial activation toward proinflammatory state (Fig. 10). Furthermore, Serpina3n cKO diminishes disease severity and demyelination in EAE (Fig. S16 a-d), and favors remyelination in CPZ (Fig. S17). Serpina3ncKO also diminishes normal aging-elicited myelin damage and axonal injury and reduces oxidative stress (Fig. S19). These findings suggest that, in the models examined, SERPINA3N may promote a detrimental microglial activation state that exacerbates tissue injury, rather than supporting beneficial debris clearance and repair.

3-3) Although the study provides extensive transcriptional evidence of Serpina3n expression in oligodendrocytes, it lacks direct functional validation of its role in modulating microglial activation. As such, the mechanistic link remains speculative. Additional evidence is required to support the proposed model, and in its current form, the manuscript is incomplete and not suitable for publication.

Response: Thank the reviewer for the comment.
As described above, in addition to Serpina3n expression in OLs (Figs. 1-3), this study defined the mechanisms underlying SERPINA3N expression in OLs (Figs. 4-7) and provided functional data validating the functional significance of oligodendroglial SERPINA3N in regulating neuroinflammation, glial activation, and demyelination in the diseased (demyelinating) and non-diseased (normal aging) CNS (Figs. 8-10, Suppl Figs. 16, 17, 19). Please see Responses to comment #2 for details. We greatly appreciate the reviewer's effort in improving our manuscript.

Reviewer #4 (Remarks to the Author):

In this study Wang and colleagues investigate an oligodendrocyte subpopulation, characterized by the upregulation of serpina3n, herein termed SerpinOLs, that was previously demostrated in various studies as a disease-associated subpopulation in several conditions in mice and human brain samples. Importantly, Serpina3n was also previously reported to be expressed in disease-associated astrocytes. This study further extend these findings, it performes a comprehensive analysis of SerpinOLs in additional conditions. The relevance and novelty of this study relys on the investigation of the molecular mechanisms and the function of serpinOLs. Authors show that serpina3n are mainly expressed by oligodendrocytes, and SerpinOLs are driven by direct oligodendrocyte injury, not inflammation. STAT3 activation seems to be critical for the expression of serpina3n in oligodendrocytes. Functionally, authors show that SerpinOLs-derived SERPINA3N amplifies pro-inflammatory responses and microglial activation in the context of disease or injury, suggesting that oligodendrocytes contribute to CNS pathology beyond myelination.

This is a timely and relevant study that advances our understanding on the serpinOLs functions and mechanisms of action across various disease and injury contexts, and it highlights a potential new target for immune modulation.

Response: We thank the reviewer for pointing out the significance and innovation of the study. We revised the manuscript according to your insightful comments as shown below.

Questions and comments:

1) In general quantifications in control conditions are missing in the Figs data. According to RNA there is expression of Serpina3n in OL and astrocytes (neurons were not checked? Also reported to expressed serpina3n). Did authors estimate SERPINA3N+ cells in control conditions? It is important to determine if the results represent an increase in number of SERPINA3N+OLs or increased protein expression and secretion. At least quantifications such as Fig 1H and 1R should have % expression in control, if any, or report if SERPINA3N+ cells are detected at baseline.

Response: Thank you for these important comments. We have now included control groups across all models (please see Fig. 1h, Fig. 1p, Fig. 7f, Fig. S3d, Fig. S4e, Fig. S4f, Fig. S5a, Fig. S6e, k, Fig. S7c, Fig. S9b). Our results demonstrate that SERPINA3N expression is very low in adult healthy CNS (spinal cord and brain).

Quantifications of control groups were also added to Fig. 1i and Fig. 1s, showing the percentages of SERPINA3N⁺ cells in baseline control conditions. We demonstrated an increase in the number of SERPINA3N⁺ OLs, elevated protein expression, and increased secretion in injury/disease animals compared with control groups.

Additionally, we examined neuronal SERPINA3N expression. Although bulk RNA-seq datasets have reported low or detectable Serpina3n transcripts in neurons, our immunostaining did not detect SERPINA3N protein in NeuN⁺ neurons under control or diseased conditions (aging, LPS and Spinal cord trauma). These data suggest that neurons are unlikely to be a major cellular source of SERPINA3N protein in vivo during CNS injury or demyelination.

2) In general, info on which spinal cord region(s) were analysed for quantifications and imaging is missing.

Response: We appreciate the reviewer's comment. For clarity, we have now specified the spinal cord regions analyzed in each model. In the EAE and PlpCre/DTA models, quantifications and imaging were performed in the ventral white matter of the lumbar spinal cord. In the spinal cord trauma model, analyses were conducted in the lateral white matter caudal to the T10 injury site. These details have been added to the Materials and Methods, Results or Figure Legend sections.

3) Quantifications are needed to sustain this sentence: "We found that SerpinOLs were markedly increased in the regions of corpus callosum (Fig. 4G) and fimbria (Fig. 4H)". What were the basal levels?

Response: We thank the reviewer for this helpful suggestion. We have now included quantifications to support the statement that SerpinOLs are markedly increased in the corpus callosum and fimbria. Specifically, we quantified the percentage of SERPINA3N⁺ cells in both regions under control condition. The results show that basal SERPINA3N expression is barely detectable in healthy controls, whereas a significant increase is observed following cuprizone treatment. These quantifications have been added to the revised Fig. 3h and described in the Results text.

4) Figure 4G does not sustain this sentence: "The proximity of SerpinOLs with activated microglia (Fig. 4G-H, arrowheads) indicates that SerpinOLs may communicate with microglia through SERPINA3N"

Response: Thank you for pointing this out. We have revised the text to avoid implying evidence of direct microglia–SerpinOL communication. Instead, we now state that normal aging promotes the transition of homeostatic OLs into SerpinOLs preferentially in white matter regions with microglial activation.

5) Supp Fig 5 shows that the highest number of cells expressing SERPINA3N+ in lesion at day 1-2, are neurons, which decreased sharply at day 7 and 14, with the rise of serpinOLs. This is a very interesting finding as it highlights a dynamic temporal expression of SERPINA3N in other cell types, it is not referred to in the text. The region analysed should also be specified.

Response: We thank the reviewer for this insightful comment. Supplementary Fig. 5 indeed shows a dynamic temporal change in the cellular source of SERPINA3N following ischemic stroke in the lesion core. We have now incorporated this important observation into the revised manuscript and specified the regions analyzed. We clarified that acute SERPINA3N expression (1-2 dpi) is mainly observed in NeuN⁺ neurons in the lesional areas and Sox10+ OLs within the lesional and penumbra areas, whereas neuronal SERPINA3N⁺ cells sharply decline by 7-14 dpi as neuronal loss progresses. Concurrently, SERPINA3N expression becomes highly enriched in SOX10⁺ oligodendroglia during the chronic phase (7-14 dpi). This addition strengthens the interpretation and highlights the dynamic role of SERPINA3N in ischemic pathology.

6) Supp 6 A4-5: should keep consistency with similar plots and replace cell types by markers.

Response: We thank the reviewer for this helpful suggestion. In Supplementary Fig. 6, we have revised the graphs to maintain consistency with the other panels by replacing the cell type labels with their corresponding markers. The figure labels and legends have been updated accordingly. Please see the revised Suppl Fig. 6

7) Authors should be consistent in CD68+ cells, sometimes are called microglia other times myeloid cells. For scientific rigor and to satisfy reviewers:

Response: We appreciate the reviewer's comment regarding the description of $CD68^{+}$ cells.

CD68 primarily labels activated microglia and monocyte-derived macrophages in the diseased CNS. For scientific rigor, we referred to $CD68^{+}$ cells as activated microglia or activated microglia/macrophages in the revised manuscript.

$CD68^{+}$ cells are primarily activated microglia in CPZ, LPS, 5xFAD, and aging models, whereas in other models such as EAE, stroke, and SCI, $CD68^{+}$ cells are activated microglia and macrophages.

8) Page 10, line 7-8, text has wrong fig legends, where it says fig 8 should be Fig7.

Response: We thank the reviewer for pointing out this error. The figure citation on page 10, lines 7–8 has been corrected, and the reference to "Fig. 8" has now been changed to "Fig. 7" in the revised manuscript.

9) Did authors checked if serpinOLs are dying? For instance, in the PlpCre/DTA model? In general, in other models are serpinOL surviving OL? Was any Casp3 or Tunel staining performed in serpinOLs?

Response: We thank the reviewer for this question regarding the fate of SerpinOLs.
In most injury models examined (for example EAE, cuprizone demyelination, and aging), SerpinOLs generally retain typical oligodendrocyte morphology and rarely display obvious features of late-stage apoptosis or DNA fragmentation (cleaved Caspase 3 negative). However, we did notice that SerpinOLs (arrowheads) display diminished or fragmented pattern of Lamin B1, which is a senescence-associated biomarker (PMID: 22496421), compared with Serpina3n-negative cells in proximity (arrows). Please see Panel A and B of the figure below. At the chronic stages of EAE, some of SerpinOLs are positive for the typical marker of cell senescence P21.

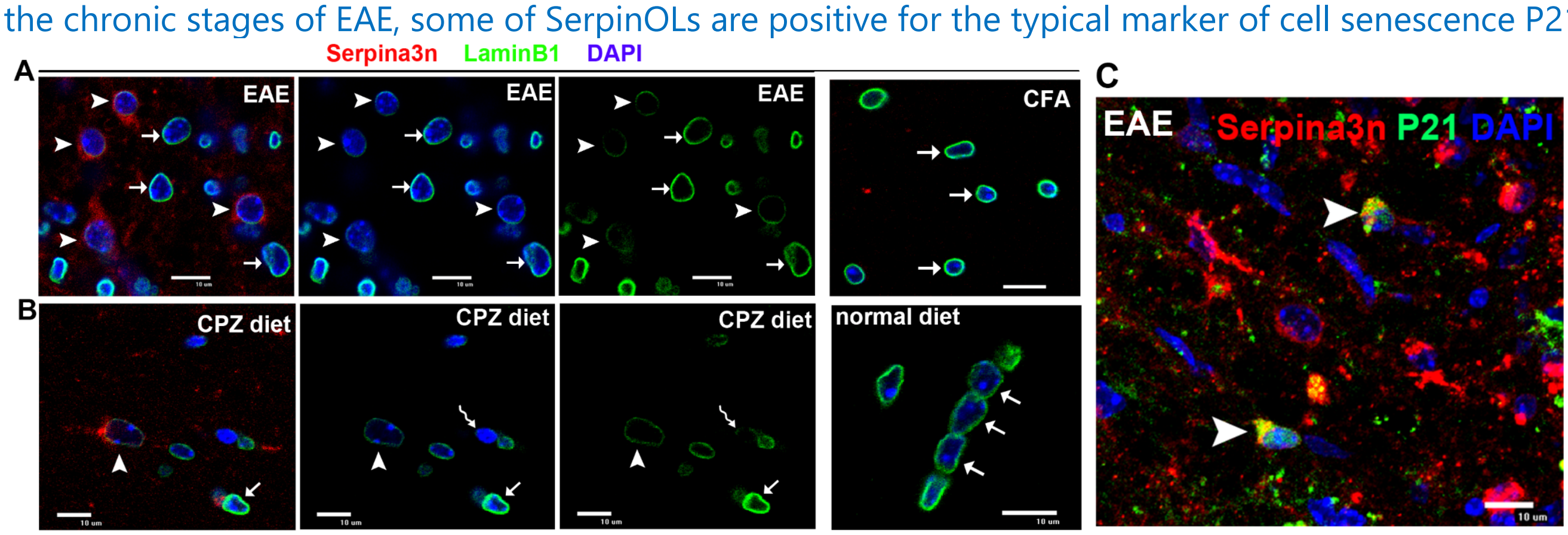

These observations suggest that SerpinOLs primarily represent a rapidly reactive, injury-responsive stage of surviving oligodendrocytes, rather than a population of cells undergoing imminent cell death. However, these observations are preliminary and have not been included in the manuscript.

10) Single cell studies: dotplot or violin plot with cell type markers should be represented intead of image in Fig supp 12A. The fibroblast population is not part of CNS, should be stated their putative origin and the markers. How were the populations of activated cells assigned? Did authors have the markers and the refrence papers? Were OL from Fig Supp 12A correlated with the OL previously described in literature?

Response: We really appreciate the reviewer for pointing out the details. Great and very insightful points! Dotplot with cell type markers of each cluster was included (please see the revised Fig. S13a). We agree with the reviewer that fibroblasts are not part of the CNS. However, they are found in the CNS, primarily in the meninges surrounding the spinal cord and brain. Since we used whole spinal cord as the starting tissue for single cell studies, they may be included and annotated based on the cell marker database CellMarker 2.0 we used (http://www.bio-bigdata.center/). As shown in Fig. S13a, they were identified by Col8a1, Bnc2, Fn1, Slc47a1, Ranbp3l, and Slc7a11, genes that are highly enriched in fibroblasts.

As shown in Fig. S13B (original Fig. S12a), cells annotated as OLs highly express SOX10 and Plp1 (see Fig. S13c).

11) Fig 8C also shows clear expression in astrocytes, check legend.

Response: We thank the reviewer for this helpful comment. We have revised the text and figure legend to accurately reflect that Serpina3n mRNA is predominantly expressed in oligodendrocyte clusters, while lower but detectable expression is also present in astrocytes, as shown in Fig. 7c. This updated description is now consistent with the displayed data.

To clarify, we agree that Serpina3n mRNA is present in astrocytes (Fig. 7c, also see Fig. 1a and Fig. 1l) even under homeostasis conditions. However, the mRNA levels in astrocytes did not increase upon CNS disease/injury (Fig. 1a and Fig. 1l) and does not seem to translate into SERPINA3N proteins as demonstrated by Serpina3n-tdTom knock-in reporter (Fig. 2) and RiboTag experiments (Fig. 1n-o).

12) In Supp 13 authors conclude that serpinOL do not express MHC-II. Although panel F conclusion was “triple IHC showing that tdTom+SOX10+ SerpinOLs (arrowheads) do not express I-A/I-E in Serpina3n-tdTom reporter mice”, one arrowhead points to a cell that has I-A/I-E green staining. As previously reported in the literature, this is a very low-abundance population that can be easily overlooked.

Response: We thank the reviewer for this careful observation. We agree that the highlighted cell in Supplementary Fig. 13f (revised Suppl Fig. 14f) seems to show weak I-A/I-E signal. However, a closer examination of the flattened image indicates that the “co-localization” may be due to the maximal projection of 3D confocal z-stack images. Indeed, there is no co-localization of tdTom and I-A/I-E in the orthogonal view at a different optical z-stack (see the orthogonal view of the same field below, arrowhead)

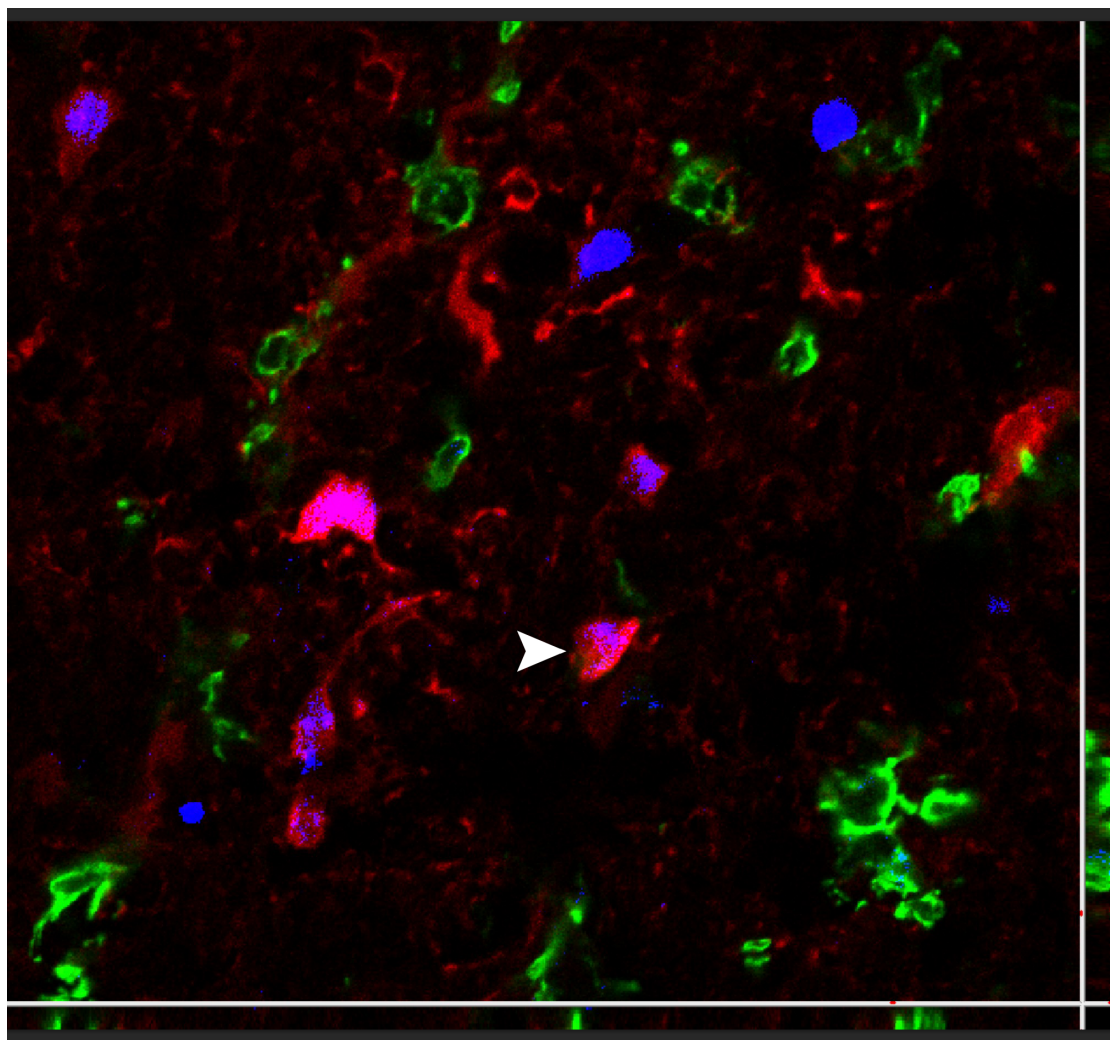

We agree with the reviewer that I-A/I-E-expressing cells are a very low-abundance population that can be easily overlooked. We therefore soften the statement in the revised manuscript as "the vast majority of tdTom^{+}SOX10^{+} SerpinOLs do not express MHC-II molecule I-A/I-E". We have updated the figure legend and Results to avoid an arbitrary statement.

13) Fig 8: were quantifications of double positive STAT3/SERPINA3N performed? Images of the double stainings would strength the conclusions.

Response: We appreciate the reviewer's insightful comment. In the original version of the manuscript, we had already included representative immunofluorescence images showing co-localization of STAT3 (red, Figure 7g) or p-STAT3 (red, Figure 7h) with SERPINA3N (green). In the revised manuscript, we have added the corresponding quantifications to complement these images (Figure 7i).

Quantitative analysis of the double staining shows that in EAE spinal cords approximately 87.7% of SERPINA3N^{+} cells are STAT3^{+} and 81.31% of SERPINA3N^{+} cells are p-STAT3^{+}, while such co-localization is rarely detected in CFA controls (Figure 7f). These added quantifications, together with the existing co-localization images, further strengthen our conclusion that SERPINA3N induction in oligodendrocytes is tightly associated with STAT3 activation in the context of EAE.

14) The scoring plot for EAE in the three conditions (Serpina3n Ctrl_CFA, Serpina3n Ctrl_MOG and Serpina3n cKO_MOG) show be shown. Was there any improvement or exhacerbation of EAE scoring in cKO? This is important to correlate with article showing and improvement in EAE scoring after SERPINA3N injection despite no observed reduction the number of infiltrating immune cells.

Response: We thank the reviewer for the comment. We have included the clinical EAE scoring data from all three conditions: Serpina3n Ctrl_CFA, Serpina3n Ctrl_MOG, and Serpina3n cKO_MOG (Figure S16a-c). These results demonstrate that Serpina3n cKO mice exhibit significantly reduced disease severity, as reflected by lower clinical scores, cumulative scores, and AUC values compared to Serpina3n Ctrl_MOG mice. Serpina3n Ctrl_CFA mice showed no disease induction. These findings strengthen our conclusion that loss of SERPINA3N is protective against EAE disease.

15) The question remains, are serpinOL protected or dying? Is OL-derived SERPINA3N detrimental or protective for disease establisment and or progression? This study would benefit from additional analysis on one models

performed, for instance, analysis of serpinOL for apoptosis markers, lesion size and/or OL/myelin loss in cKO.

Response: We thank the reviewer for raising these important points.

As described above, SerpinOLs are rarely positive for apoptosis marker cleaved caspase 3. Instead, they display cell senescence-associated markers (diminished or fragmented lamin B1 and expression of P21) (see responses to comment #9). Our functional data show that Serpina3n cKO diminished EAE-elicited demyelination (Fig. S16d) and attenuated CPZ-induced OL loss and myelin damage (Fig. S16e-f). Our data suggest that OL-derived SERPINA3N is detrimental for CNS demyelinating diseases.

We appreciate all reviewers' insightful critiques and their effort in improving our manuscript.

Thank you for considering this substantially revised manuscript.

Point-by-point responses

Reviewer #1 (Remarks to the Author):

The authors have addressed the concerns raised in the previous review. The additional experiments strengthen the study's conclusions and improve the quality of the manuscript. The reorganization of the manuscript to highlight key data from demyelination models and normal aging models in the main figures is greatly appreciated and makes for easier reading. The addition of control animals helps to convince the reader of the essential role of SERPINA3. In the legends, details regarding the quantifications performed or the number of animals used improve the quality of the article. Thus, I would like to recommend accepting this manuscript for publication.
Response: We appreciate the reviewer's positive comments.

Reviewer #3 (Remarks to the Author)

While the authors provide evidence for an increased number of mature oligodendrocytes in the Serpina3n cKO condition, this does not address the unresolved issue from my prior comments. The manuscript does not establish a causal, mechanistic link detailing how Serpina3N+ oligodendrocytes controls microglial functional states (microglia-intrinsic vs indirect effects) to produce these outcomes. The association between Serpina3N expression and microglial changes across the different models remains descriptive. While they speculate Serpina3N may drive the microglial switch toward a pro-survival phenotype, the current data do not convincingly demonstrate this switch. Evidence that Serpina3N acts directly on microglia is required to substantiate the proposed pro-survival/remyelination effects.

Response: We thank the reviewer for this insightful comment and for emphasizing the need for a more direct mechanistic link between oligodendrocyte-derived SERPINA3N and microglial functional state.

We agree that this was a key issue requiring stronger mechanistic support. In this revision, we have added new experiments that directly address this concern and demonstrate that SERPINA3N can act directly on microglia to promote a pro-inflammatory activation state.

First, in Fig. 10a-g, we show that addition of recombinant SERPINA3N to purified primary microglia is sufficient to enhance expression of pro-inflammatory markers, indicating that SERPINA3N can act directly on microglia rather than exclusively through indirect secondary mechanisms. Importantly, cultured primary microglia do not express detectable Serpina3n, supporting the conclusion that the observed effects arise from exogenous SERPINA3N rather than an autocrine microglial source.

Second, to define the underlying mechanism, we performed additional studies in Fig. 10h-o. We found that recombinant SERPINA3N physically interacts with TLR4, as demonstrated by proximity ligation assay (PLA) (Fig. 10h) and co-immunoprecipitation using anti-TLR4 (Fig. 10i) and anti-SERPINA3N (Fig. 10j) antibodies. Functionally, SERPINA3N potentiated the expression of multiple TLR4-responsive pro-inflammatory genes in activated microglia, including Tnfα (Fig. 10k), IL-1b (Fig. 10l), iNOS (Fig. 10m), Cxcl10 (Fig. 10n), and Ptgs2 (Fig. 10o). Moreover, pharmacological inhibition of TLR4 signaling with TAK-242 significantly attenuated the SERPINA3N-dependent enhancement of these inflammatory mediators (Fig. 10k-o).

Together, our results support a model in which SERPINA3N released from oligodendrocytes acts directly on microglia, engages TLR4, and potentiates TLR4-dependent inflammatory signaling. We have revised the Results and Discussion accordingly to make this mechanistic link clearer.

Reviewer #4 (Remarks to the Author):
The authors have addressed all my questions and concerns. Congratulations for this significant work.
Response: We appreciate the reviewer's positive comments.
