# Supplementary material for: Injury-transduced oligodendrocytes modulate neuroinflammation and glial activation in diseased and non-diseased central nervous system
Source: Nat Commun. 2026 Aug 29;17:10347. doi: 10.1038/s41467-026-76159-2 (PMC13623841; doi:10.1038/s41467-026-76159-2)

Title

Injury-transduced oligodendrocytes modulate neuroinflammation and glial activation in diseased and non-diseased central nervous system

Author list

Yan Wang[1,2,#,*], Meina Zhu[1,2,#], Joohyun Park[1,2,#], Hyun Kyu Kim[1,2], Xiong Ge[1], Xi Chen[3,4], Bo Chen[5], Shinghua Ding[6], Wensheng Lin[7], Fuzheng Guo[1,2,*]

Affiliations

[1]Department of Neurology, School of Medicine, University of California, Davis, Sacramento, CA, USA; [2]Institute for Pediatric Regenerative Medicine (IPRM), Shriners Hospitals' for Children – Northern California, Sacramento, CA, USA; [3]Institute of Neurology, Sichuan Provincial People's Hospital, Chengdu, China; [4]School of Medicine, University of Electronic Science and Technology of China, Chengdu, China; [5]Department of Neuroscience, Cell Biology and Anatomy, University of Texas Medical Branch, Galveston, TX, USA; [6]Department of Chemical and Biomedical Engineering, University of Missouri, Columbia, MO, USA; [7]Department of Neuroscience, University of Minnesota, MN, USA.

[#]These authors contributed equally: Yan Wang, Meina Zhu, Joohyun Park

*Corresponding authors: Yan Wang (yyawang@health.ucdavis.edu), Fuzheng Guo (fzguo@health.ucdavis.edu).

Yan Wang https://orcid.org/0000-0002-7744-4078

Fuzheng Guo https://orcid.org/0000-0003-3410-8389

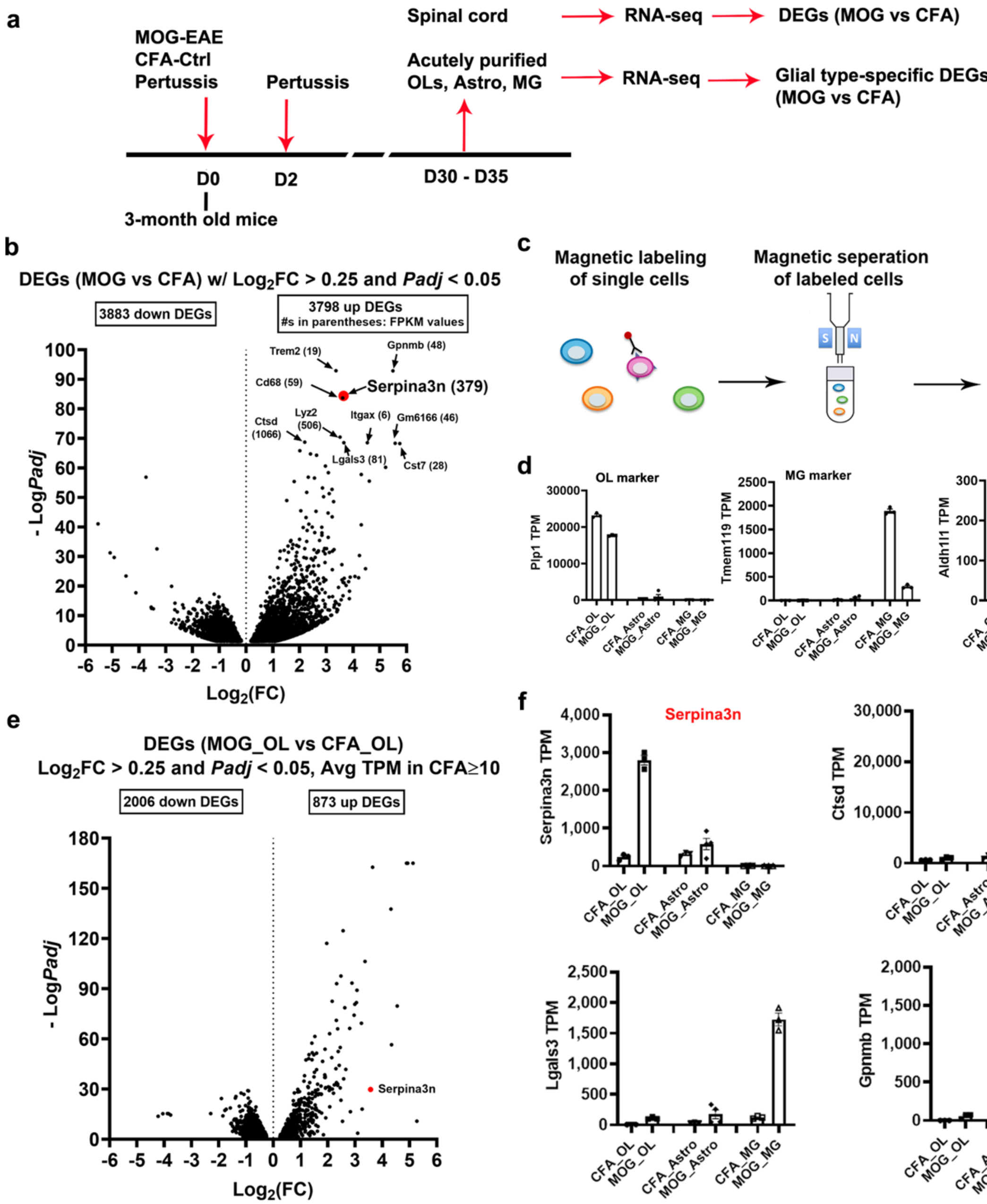

Supplementary Figure 1 – Identification of glial population-specific differentially expressed genes (related to Figure 1).

a Experimental design. Inflammatory demyelination of experimental autoimmune encephalomyelitis (EAE) was induced in adult mice via MOG immunization (CFA as control) at day 0 (D0), with pertussis administered i.p. on D0 and D2. RNA-seq was performed on whole spinal cords (**b**) and MACS-purified glial populations (**c-f**) at D30-D35. OLs, oligodendrocytes; Astro, astrocytes; MG, microglia.

b Volcano plot of differentially expressed genes (DEGs) in spinal cord of MOG/EAE (n=4 mice) vs. CFA mice (n=3 mice) at D35. Top10 DEGs are labeled with gene symbols and FPKM values.

c Schematic of magnetic beads-assisted cell sorting (MACS) of spinal cord glia at D30. OL, Astro, and myeloid cells (including MG) were isolated using anti-O4, anti-ACSA2, and anti-CD11b magnetic beads, respectively.
d Purity validation of sorted population using representative markers *Plp1* (OL), *Tmem119* (MG), and *Aldh1l1* (Astro). Expression is shown as TPM (transcripts per million). *n* = 3 mice per group for CFA_OL, MOG_OL, CFA_Astro, CFA_MG and MOG_MG; *n* = 4 mice for MOG_Astro.
e Volcano plot of DEGs in MOG_OL versus CFA_OL. *Serpina3n* is highlighted in red.
f TPM expression of *Serpina3n*, *Ctsd*, *Lgals3*, and *Gpnmb* in OLs, Astro, and MG from MOG/EAE and CFA mice. *Serpina3n* is specifically upregulated in OL, while *Ctsd*, *Lgals3*, and *Gpnmb* are enriched and upregulated in MG. *n* = 3 mice per group for CFA_OL, MOG_OL, CFA_Astro, CFA_MG and MOG_MG; *n* = 4 mice MOG_Astro.
Data are presented as mean values ± SEM. Source data are provided as a Source Data file.

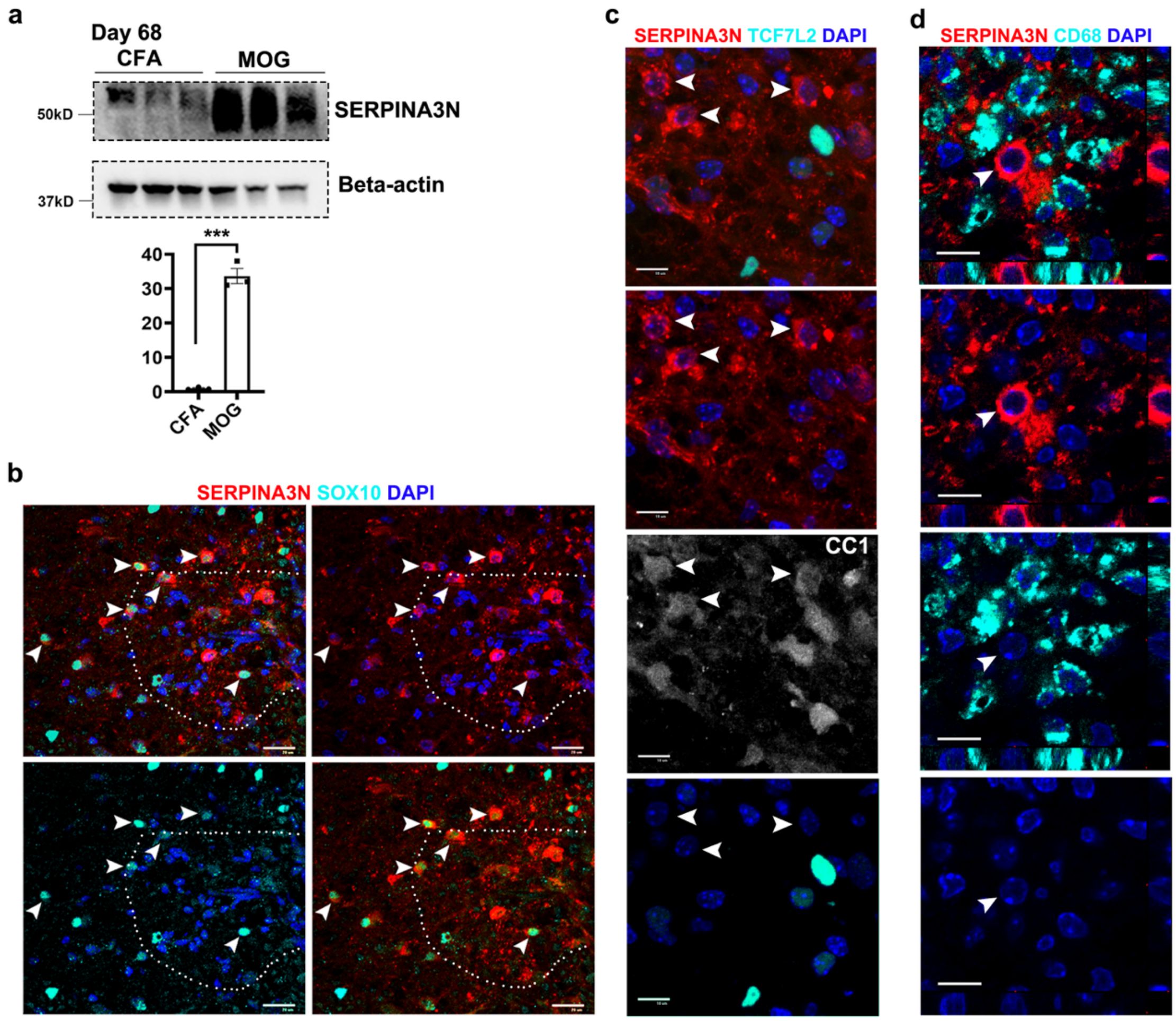

Supplementary Figure 2 – SERPINA3N persists into chronic phase of inflammatory demyelination (related to Figure 1).

a Western blot showing SERPINA3N levels in spinal cords. β-actin was run on a separate gel using the same samples and is shown as a sample processing control. *** *P* = 0.0001. *n* = 3 mice per group. Statistical significance was determined using a two-tailed unpaired Student's t-test.

b Immunofluorescence of SERPINA3N and SOX10 in MOG/EAE spinal cord. Dotted regions indicate inflammatory lesions with dense DAPI$^+$ nuclei. Arrowhead, SERPINA3N$^+$SOX10$^+$ cells.

c Confocal images of SERPINA3N, CC1, and newly regenerated OL marker TCF7l2 in MOG/EAE spinal cord. Evenly distributed DAPI$^+$ cells indicate non-lesional areas. Arrowheads highlight SerpinOLs lacking TCF7l2 expression.

d Orthogonal view of confocal images of SERPINA3N and the activate myeloid cell marker CD68. Arrowhead points to a SerpinOL adjacent to CD68$^+$ cells.

All panels: D68 post-MOG (or CFA). Nuclei counterstained with DAPI (blue), Scale bars: **b** 20 µm, **c, d** 10 µm.
Data are presented as mean values ± SEM. Source data are provided as a Source Data file.

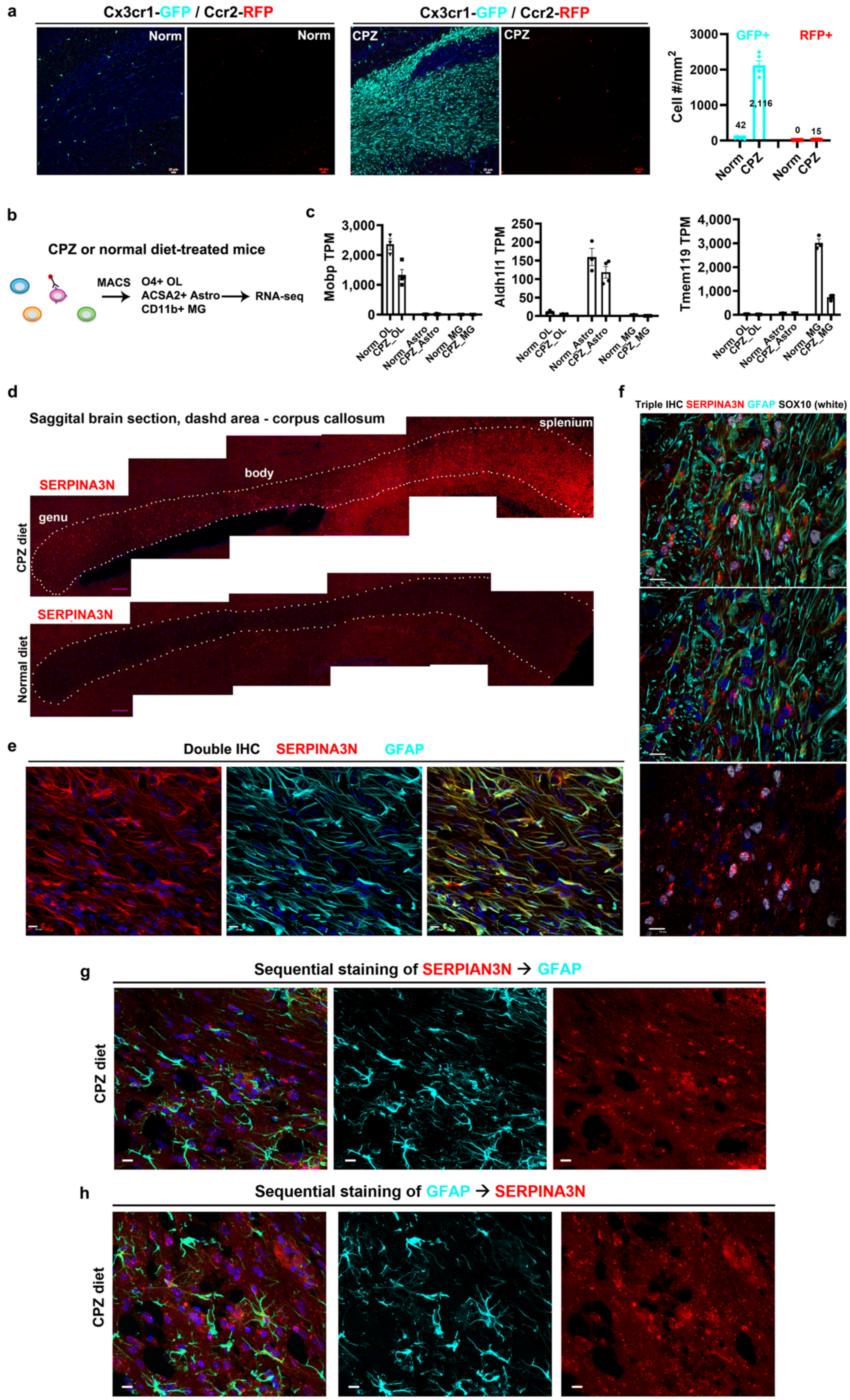
a
Cx3cr1-GFP / Ccr2-RFP
Norm
CPZ
GFP+
RFP+
Cell #/mm2
42
2,116
0
15
b
CPZ or normal diet-treated mice
MACS
O4+ OL
ACSA2+ Astro
CD11b+ MG
RNA-seq
c
Mobp TPM
Aldh1l1 TPM
Tmem119 TPM
Norm_OL
CPZ_OL
Norm_Astro
CPZ_Astro
Norm_MG
CPZ_MG
d
Saggital brain section, dashd area - corpus callosum
SERPINA3N
genu
body
splenium
CPZ diet
Normal diet
e
Double IHC SERPINA3N GFAP
f
Triple IHC SERPINA3N GFAP SOX10 (white)
g
Sequential staining of SERPIAN3N → GFAP
CPZ diet
h
Sequential staining of GFAP → SERPINA3N
CPZ diet

Supplementary Figure 3 – SERPINA3N expression in the corpus callosum during cuprizone (CPZ)-induced demyelination (related to Figure 1).

a Fluorescent IHC and quantification of GFP^+ (resident microglia and/or peripheral monocyte-derived macrophages) and RFP^+ (peripheral monocyte-derived macrophages) in the corpus callosum of Cx3cr1-GFP/Ccr2-RFP mice after 4 weeks CPZ or Norm diet. CPZ-induced demyelination showed robust GFP^+/RFP^- microglial expansion and rare RFP^+ infiltrated myeloid cells. DAPI counterstaining (blue). Scale bars: 20 μm. *n* = 5 mice per group.

b-c MACS purification of brain OLs, Astro, and MG of normal (Norm) or 4 weeks CPZ diet (**b**) and purity assessment using lineage-specific markers (*Mobp* for OLs, *Aldh1l1* for Astro, *Tmem119* for MG) (**c**). TPM (transcripts per million) values are from RNA-seq. *n* = 3 mice per group for Norm_OL, Norm_Astro, Norm_MG and CPZ_MG; *n* = 4 mice per group for CPZ_OL and CPZ_Astro.

d Single fluorescent IHC showing SERPINA3N induction along the splenium-to-genu gradient in CPZ (4 wks) corpus callosum (dotted regions), with no or little expression in Norm mice. Scale bars: 200 μm.

e Double fluorescent IHC showing near-complete overlap of SERPINA3N with reactive astrocyte marker GFAP in 4 weeks CPZ corpus callosum. DAPI counterstaining (blue). Scale bars: 10 μm.

f Triple fluorescent IHC showing no overlap of SERPINA3N with GFAP, but co-localization with oligodendroglial marker SOX10 in CPZ (4 wks) corpus callosum. DAPI counterstaining (blue). Scale bars: 10 μm. Note the distinct SERPINA3N staining patterns in panel **e** vs. **f**.

g-h Sequential IHC in CPZ diet corpus callosum with reversed staining order to validate SERPINA3N-GFAP signal overlap. SERPINA3N staining performed first, followed by GFAP (**g**). GFAP staining performed first, followed by SERPINA3N (**h**). Scale bars: 10 μm.

Data are presented as mean values ± SEM. Source data are provided as a Source Data file.

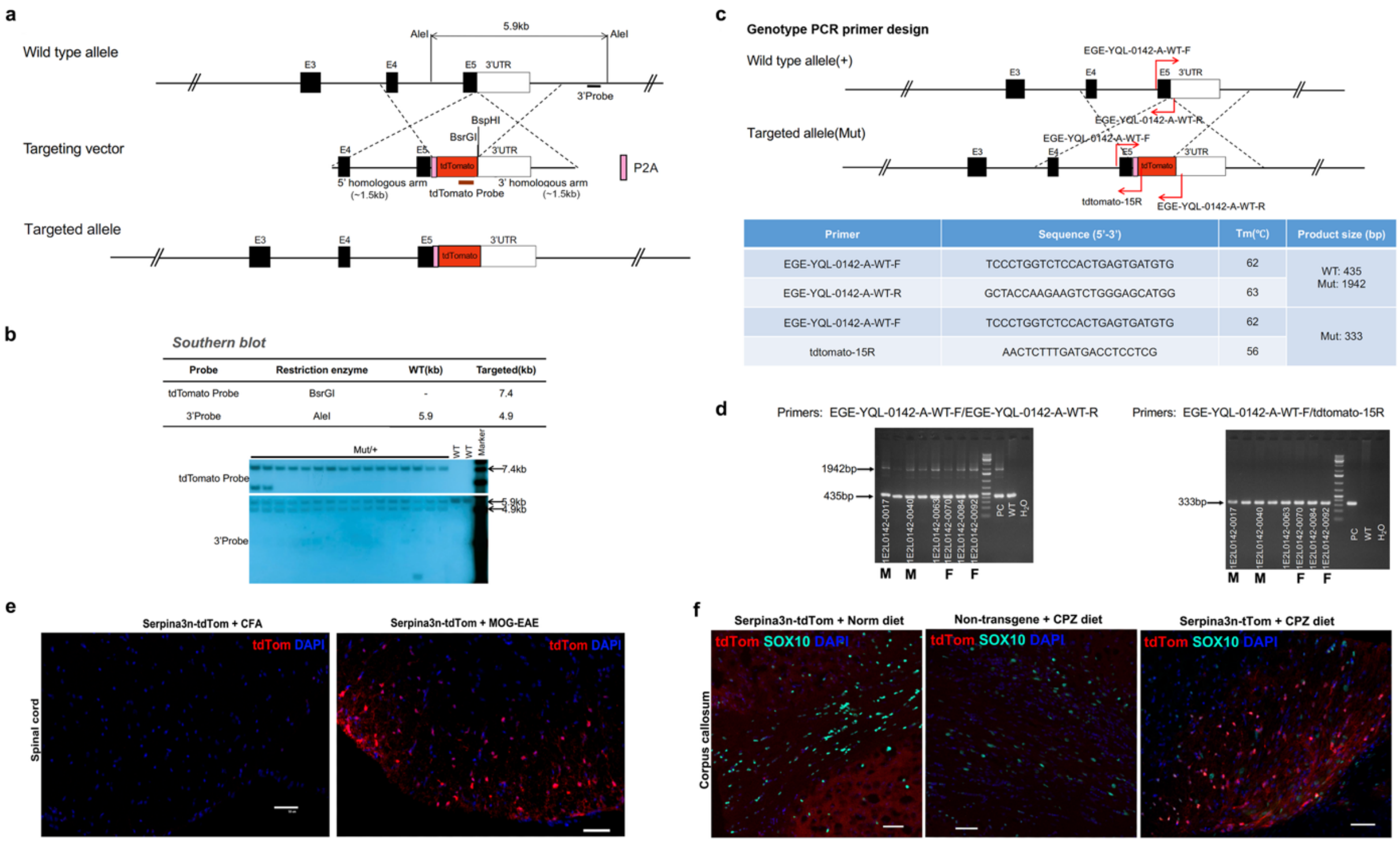

Probe	Restriction enzyme	WT(kb)	Targeted(kb)
tdTomato Probe	BsrGI	-	7.4
3'Probe	AleI	5.9	4.9

Primer	Sequence (5'-3')	Tm(°C)	Product size (bp)
EGE-YQL-0142-A-WT-F	TCCCTGGTCTCCACTGAGTGATGTG	62	WT: 435 Mut: 1942
EGE-YQL-0142-A-WT-R	GCTACCAAGAAGTCTGGGAGCATGG	63	
EGE-YQL-0142-A-WT-F	TCCCTGGTCTCCACTGAGTGATGTG	62	Mut: 333
tdtomato-15R	AACTCTTTGATGACCTCCTCG	56	

Supplementary Figure 4 – Generation of Serpina3n-tdTomato reporter mice (related to Figure 2).

a Schematic of the wild-type (WT) *Serpina3n* allele and targeting construct. The *Serpina3n* stop codon was replaced with a *P2A-tdTom* sequence, allowing expression of separate SERPINA3N and tdTom proteins. Southern Blot probe locations (tdTom probe and 3' probe) are indicated.

b Southern Blot confirming successful *Serpina3n-tdTom* integration F1 mice.

c Genotyping primer sequences and expected PCR product sizes. The EGE-YQL-0142-A-WT-F/tdtomato-15R pair amplifies a 333 bp product from the mutant allele; EGE-YQL-0142-A-WT-F/EGE-YQL-0142-A-WT-R amplifies a 435 bp WT product and a 1942 bp mutant product.

d Agarose gel showing PCR products for T and mutant alleles.

e Fluorescent IHC of tdTom in the spinal cord of Serpina3n-tdTom mice at D30 post-MOG/EAE (or CFA). Note that tdTom is induced by MOG/EAE but absent in CFA controls. Scale bar: 50 μm.

f Fluorescent IHC of tdTom in the corpus callosum of Serpina3n-tdTom mice or non-transgenic mice after 4-week CPZ or normal (Norm) diet. tdTom is detected only in CPZ-treated Serpina3n-tdTom mice. Scale bar: 50 μm.

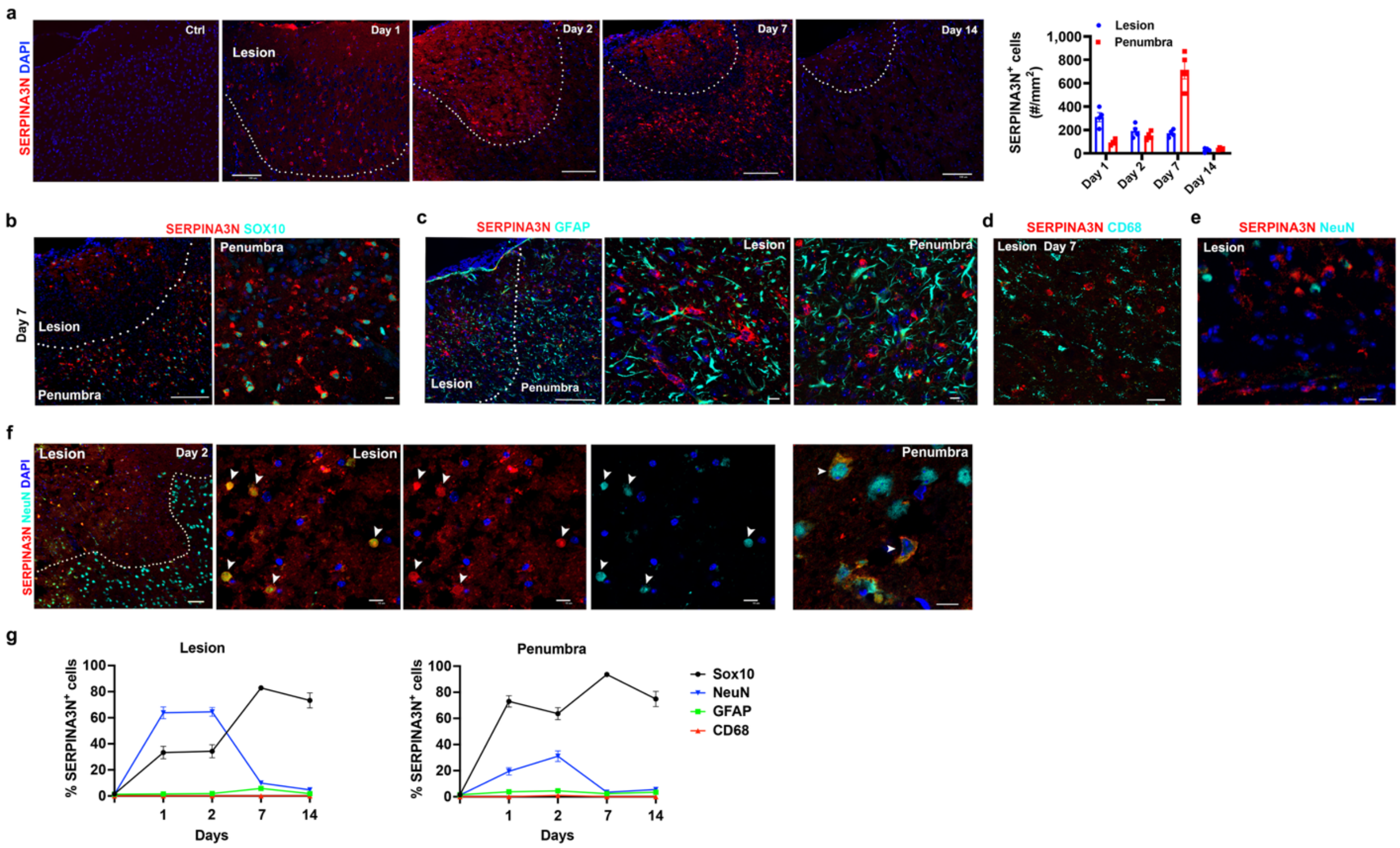

Supplementary Figure 5 – SERPINA3N expression and cellular identity in photothrombotic stroke.

a Spatiotemporal dynamic of SERPINA3N after stroke injury. Representative IHC images show SERPINA3N distribution in the cortex of control mice and at 1-, 2-, 7-, and 14-days post-injury (dpi). The white dotted line delineates the lesion core and surrounding penumbra. Right, quantification of SERPINA3N$^+$ cell density in the lesion core versus penumbra over time, showing a peak at 7 dpi with enrichment in the penumbra. Scale bars: 100 μm. *n* = 4 mice per group.

b-e Cellular identity at the peak of SERPINA3N expression (7 dpi). Double immunostaining for SERPINA3N with markers for OLs (**b**, SOX10), astrocytes (**c**, GFAP), activated microglia/macrophages (**d**, CD68), and neurons (**e**, NeuN) indicates that SERPINA3N is predominantly expressed by SOX10$^+$ oligodendrocytes in the penumbra, with minimal co-localization with GFAP, CD68, or NeuN. Scale bars: 100 μm (low magnification), 10 μm (high magnification).

f Neuronal SERPINA3N expression during the acute phase (2 dpi). Representative images from the lesion core and penumbra show SERPINA3N co-localization with NeuN$^+$ neurons (arrowheads) within the lesion core at this early time point. This localization coincides with the rapid neuronal damage and loss that characterizes the acute ischemic core. Scale bars: 10 μm.

g Temporal shift in SERPINA3N cellular source during stroke progression. Quantification of SERPINA3N$^+$ cell types in the lesion and penumbra over time reveals a transition from predominant neuronal (NeuN$^+$) expression during the acute phase (1-2 dpi) to predominant oligodendroglial (SOX10$^+$) expression during the chronic phase (7-14 dpi). GFAP$^+$ astrocytes contribute <10% of SERPINA3N$^+$ cells, and CD68$^+$ cells show negligible contribution across the time course. *n* = 4 mice per group.

Collectively, SERPINA3N exhibits a marked spatiotemporal shift after photothrombotic stroke, from acute neuronal expression in the lesion core to chronic oligodendroglial expression in both lesion and penumbra during tissue remodeling. Data are presented as mean values ± SEM. Source data are provided as a Source Data file.

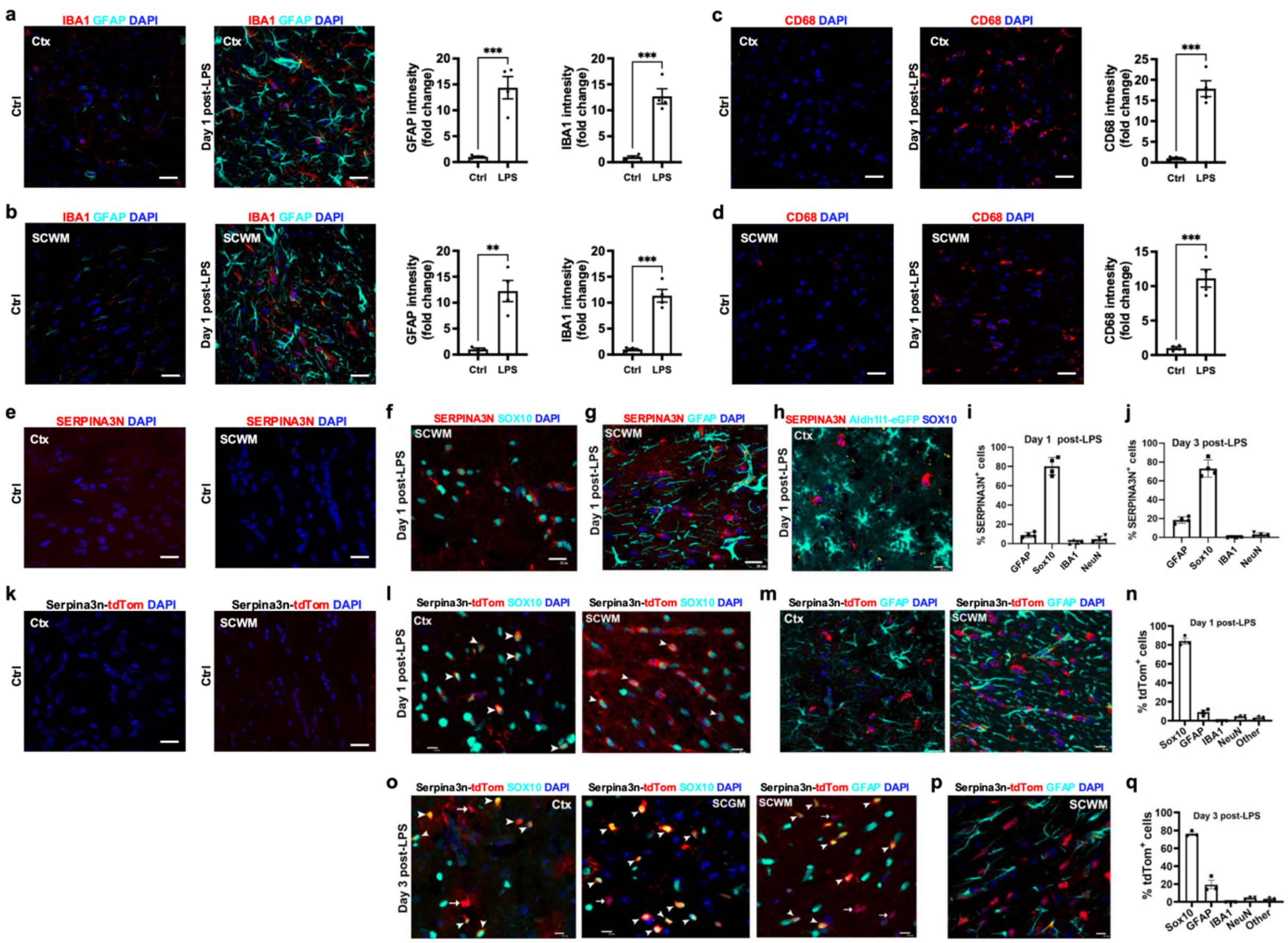

Supplementary Figure 6 – SERPINA3N expression and cellular identity in LPS-induced endotoxicity.

a-b Systemic LPS induces widespread neuroinflammation and glial activation. Representative IHC images of the astrocyte marker GFAP and microglia marker IBA1 (**a, b**), or the activated microglia/macrophage marker CD68 (**c, d**) in the cortex (Ctx) and subcortical white matter (SCWM) from saline-treated control (Ctrl) and mice at 1-day post-LPS injection. Quantification of fluorescence intensity demonstrates a robust increase in GFAP, IBA1, and CD68 signal in both the Ctx and SCWM after LPS, confirming acute neuroinflammation with prominent astrogliosis and microglial activation. (**a**) GFAP, *** P = 0.0008; IBA1, *** P = 0.0002. (**b**) GFAP, ** P = 0.0016; IBA1, ***P = 0.0002. (**c**) CD68, ***P = 0.0001. (**d**) CD68, ***P = 0.0002. n = 4 mice per group.cStatistical significance was determined using a two-tailed unpaired Student's t-test.

e-j Oligodendrocytes are the predominant source of SERPINA3N after LPS. Representative images show low basal SERPINA3N levels in the Ctx and SCWM of Ctrl mice (**e**). At Day 1 post-LPS, double/triple IHC in Ctx and SCWM reveals extensive co-localization of SERPINA3N with the OL marker SOX10 (**f**), with limited overlap with GFAP (**g**) or the astrocyte reporter Aldh1l1-eGFP (**h**). Quantification shows that >70% of SERPINA3N$^+$ cells are SOX10$^+$ OLs at 1 and 3 days post-injection, whereas <20% are GFAP$^+$ or Aldh1l1-eGFP$^+$ astrocytes; IBA1$^+$ myeloid cells or NeuN$^+$ neurons contribute minimally (**i-j**). Scale bars: C1-C5, 10 μm. n = 4 mice per group.

k-q Validation in *Serpina3n*-tdTom reporter mice. Compared to Ctrl mice (**k**), tdTom$^+$ cells at 1 day post-LPS in the Ctx and SCWM predominantly co-express SOX10 (**l**, arrowheads) rather than GFAP (**m**). This pattern persists at 3 days post-LPS (**o-p**). Quantification confirms that ~70-80% of tdTom$^+$ cells are SOX10$^+$ OLs at both time points, whereas <20% are GFAP$^+$ astrocytes (**n, q**). Scale bars: 10 μm. n = 3 mice per group.

Data are presented as mean values ± SEM. Source data are provided as a Source Data file.

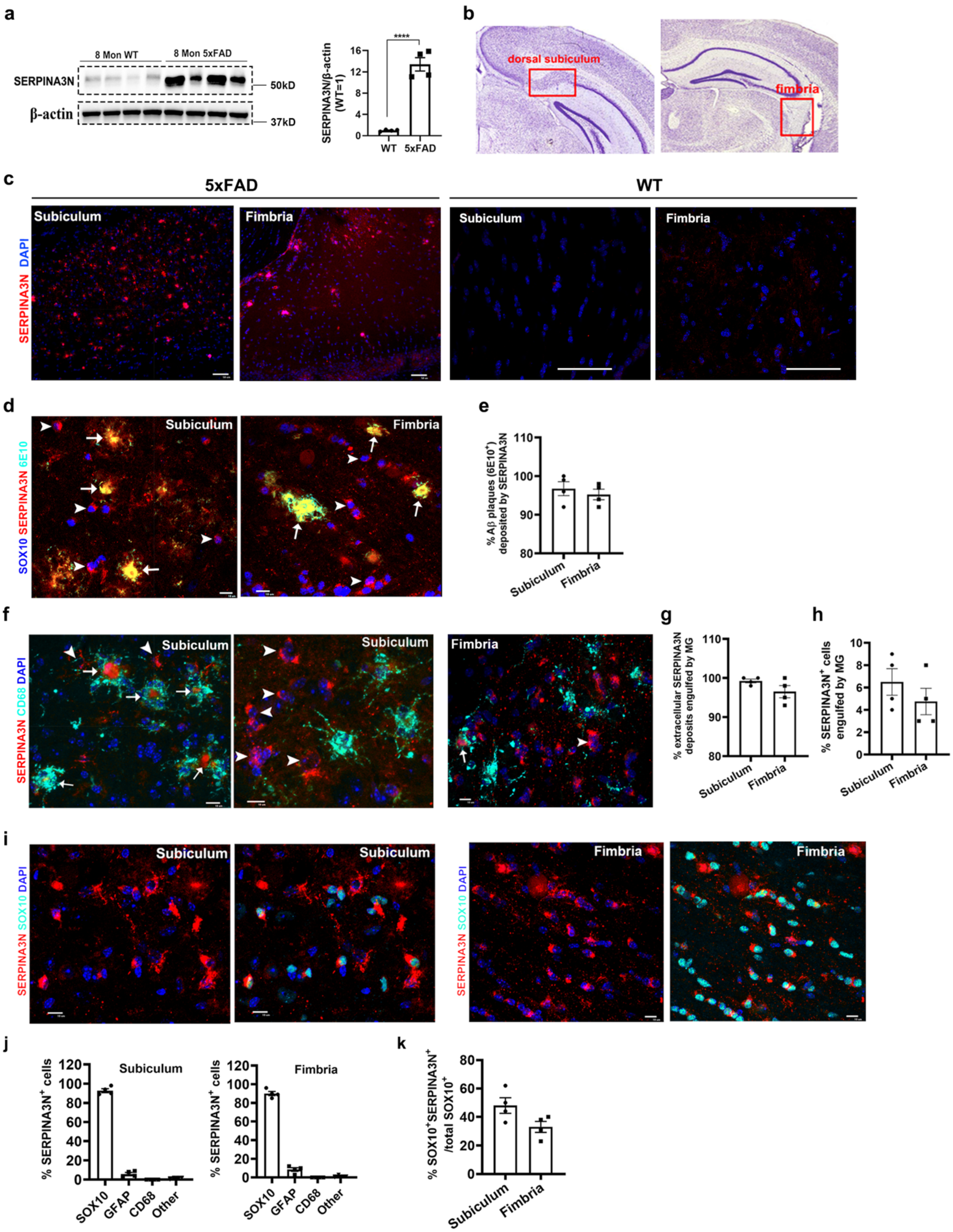

Supplementary Figure 7 – Association of extracellular and intracellular SERPINA3N with beta-amyloid plaques and oligodendroglia.

a Western blot analysis of brain lysates from 7-8-month-old 5xFAD and wild-type (WT) mice reveals a marked elevation of SERPINA3N protein levels in 5xFAD mice compared to age-matched controls. β-actin was run on a separate gel using the same samples and is shown as a sample processing control. **** $P < 0.0001$. $n = 4$ mice per group. Statistical significance was determined using a two-tailed unpaired Student's t-test.

b Representative low-magnification coronal brain sections indicating the specific hippocampal regions selected for analysis: the dorsal subiculum and fimbria (highlighted by red boxes).
c Representative IHC images of the subiculum and fimbria regions stained for SERPINA3N and DAPI. Consistent with Western blot results, dense SERPINA3N immunoreactivity is observed in the subiculum and fimbria of 7-8-month-old 5xFAD mice, whereas the signal is minimal in WT controls. Scale bars: 10 μm.
d Triple IHC staining of the subiculum and fimbria in 5xFAD mice using antibodies against SOX10, SERPINA3N, and β-amyloid (6E10). Arrows indicate the co-localization of extracellular SERPINA3N with β-amyloid plaques. Arrowheads highlight intracellular SERPINA3N accumulation within $SOX10^+$ OLs. Scale bars: 10 μm.
e Quantification of the percentage of β-amyloid plaques (6E10) that are positive for SERPINA3N immunoreactivity. The data demonstrates that the vast majority of amyloid plaques in both the subiculum and fimbria are co-localized with SERPINA3N. $n = 4$ mice per group.
f Representative double IHC images show clear co-localization of intracellular SERPINA3N within $SOX10^+$ cells.
g Quantification reveals that more than 90% of intracellular $SERPINA3N^+$ cells are $SOX10^+$ OLs, with negligible expression found in astrocytes ($GFAP^+$), microglia ($CD68^+$), or other cell types. Scale bars: 10 μm. $n = 4$ mice per group.
h Proportion of the total OL population that express SERPINA3N. Approximately 30-50% OLs in the subiculum and fimbria of 7-8-month-old 5xFAD mice have transitioned into SerpinOLs. $n = 4$ mice per group.
Collectively, these data identify OLs as the major cell type responding to AD-like amyloidosis through dysregulating SERPINA3N expression.
Data are presented as mean values ± SEM. Source data are provided as a Source Data file.

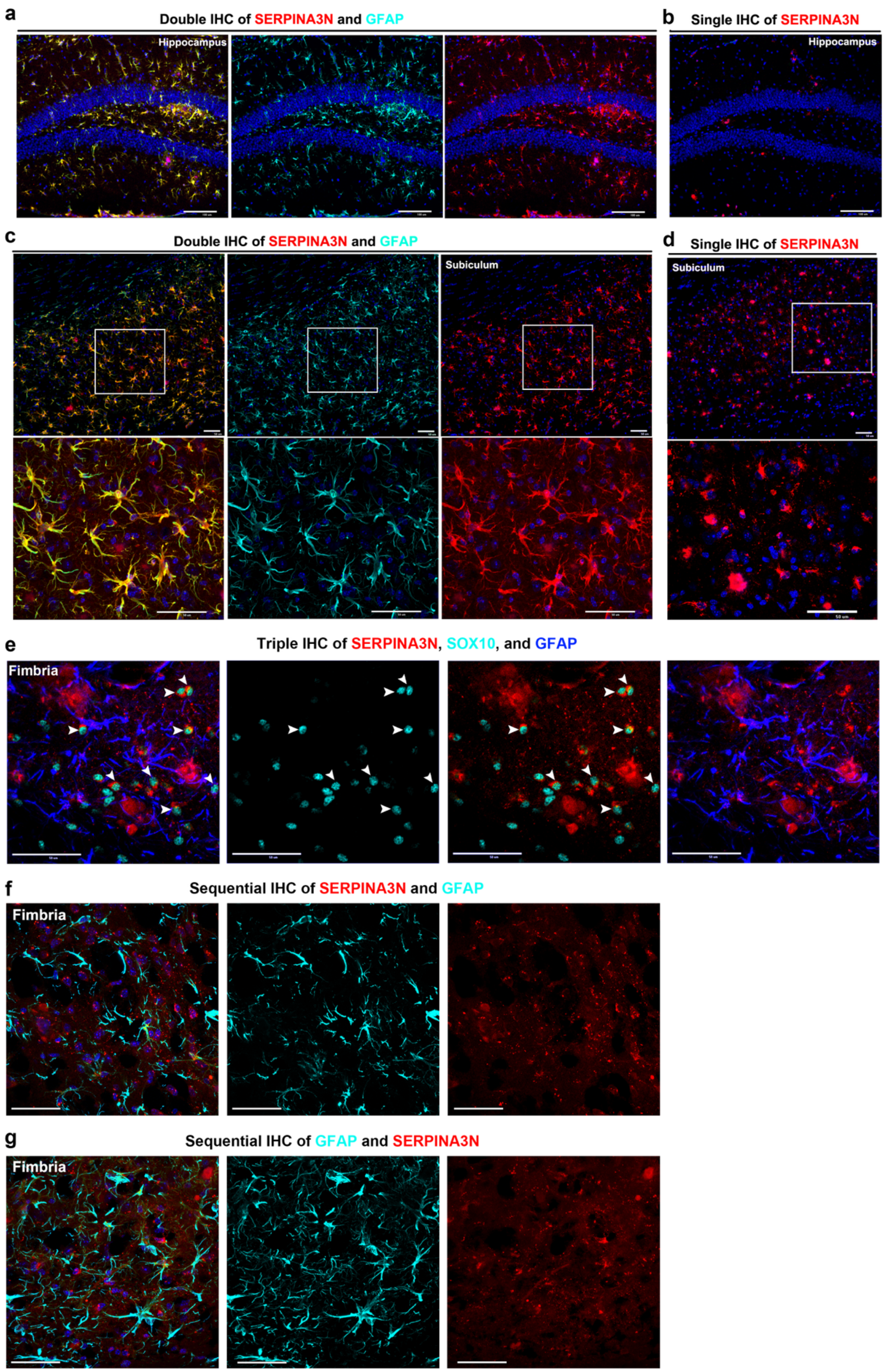

a
Double IHC of SERPINA3N and GFAP
Hippocampus
b
Single IHC of SERPINA3N
Hippocampus
c
Double IHC of SERPINA3N and GFAP
Subiculum
d
Single IHC of SERPINA3N
Subiculum
e
Triple IHC of SERPINA3N, SOX10, and GFAP
Fimbria
f
Sequential IHC of SERPINA3N and GFAP
Fimbria
g
Sequential IHC of GFAP and SERPINA3N
Fimbria

Supplementary Figure 8 – Evidence of SERPINA3N immunoreactivity artifacts in $GFAP^+$ astrocytes.

a-b Comparison of double vs. single IHC on adjacent hippocampal sections from 7-8-month-old 5xFAD mice. Double IHC for SERPINA3N and GFAP shows an apparent complete overlap (yellow signal) (**a**). Single IHC for SERPINA3N reveals a distinct punctate and cellular expression pattern that does not resemble astrocytic morphology (**b**).

c-d Double IHC again suggests extensive co-localization of SERPINA3N with $GFAP^+$ astrocytes in the subiculum (**c**). Single IHC for SERPINA3N on the adjacent section confirms the signal is not astrocytic (**d**). Boxed areas shown at higher magnification.

e Triple IHC of SERPINA3N, SOX10, and GFAP in the hippocampal fimbria. Arrowheads indicate that SERPINA3N genuinely co-localizes with $SOX10^+$ OLs, whereas the signal is distinct from $GFAP^+$ astrocytic processes.

f-g Sequential IHC staining was performed to exclude potential antibody cross-reactivity or steric hindrance artifacts. Sections were stained for SERPINA3N first followed by GFAP (**f**), or for GFAP first followed by SERPINA3N (**g**). In both sequences, SERPINA3N signal remained punctate and spatially distinct from the stellate morphology of GFAP+ astrocytes, with no observable overlap (yellow). These data indicate that the apparent co-localization observed in simultaneous double staining reflects a technical artifact.

Scale bars: **a, b** 100 μm; **c-g** 50 μm.

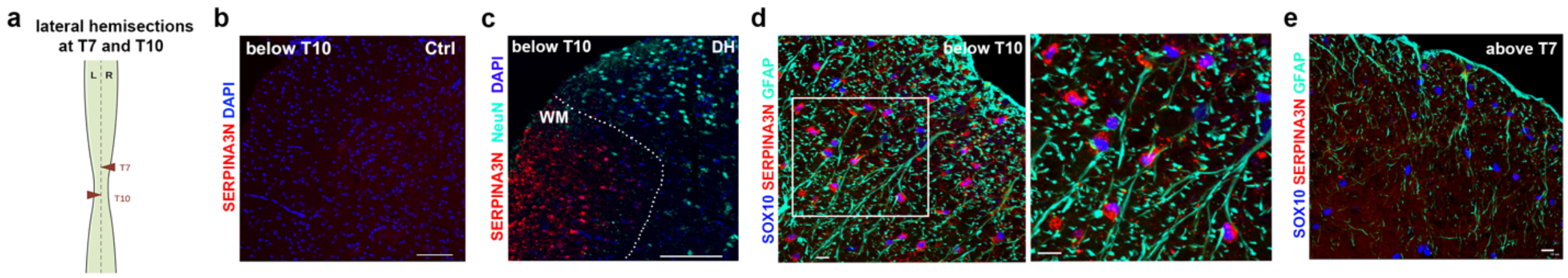

Supplementary Figure 9 – Oligodendrocytes transition into SerpinOLs in response to CNS trauma.

a Schematic of the lateral spinal cord hemisection at thoracic (T) segments T7 and T10, which disrupts innervation below T10.

b-c Analysis of SERPINA3N expression 10 weeks post-injury. Compared to controls which show minimal expression (**b**), the injured spinal cord displays robust SERPINA3N induction specifically in the white matter (WM) below the T10 lesion (**c**). Note that expression is absent in the NeuN$^+$ gray matter dorsal horn (DH).

d-e Triple IHC for SERPINA3N, SOX10, and GFAP in segments above T7 (**d**) and below T10 (**e**) identifies the cellular source of SERPINA3N. The high-magnification view (boxed area in E) demonstrates that SERPINA3N co-localizes with SOX10$^+$ OLs rather than GFAP$^+$ astrocytes.

Mice were analyzed 10 weeks post-SCI. Scale bars: 10 µm.

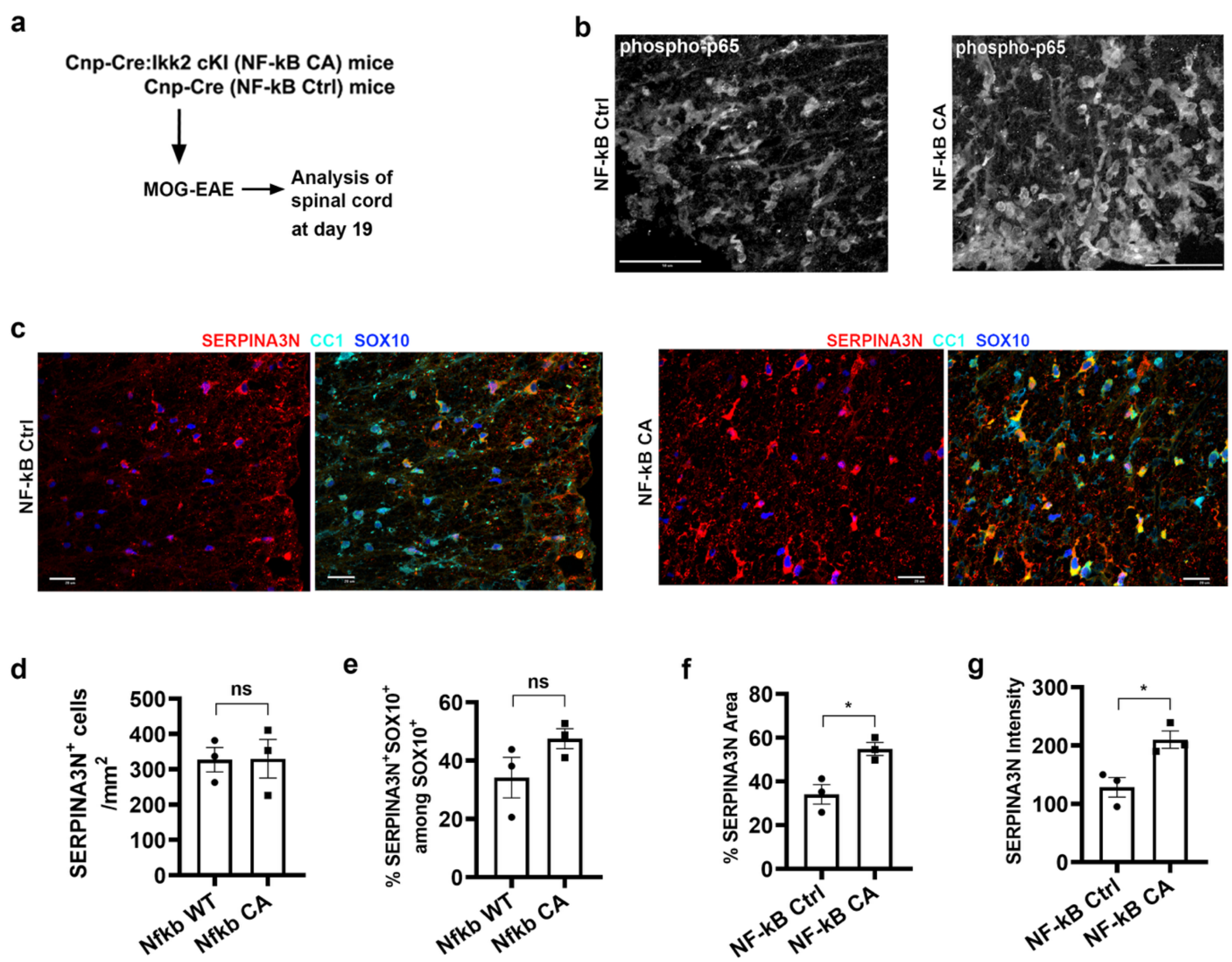

Supplementary Figure 10 - CNS inflammation enhances SERPINA3N expression under demyelinating conditions (related to Figure 4).

a Experimental design for panels **b-g**. MOG/EAE injury was induced to adult NFkB CA and NFkB Ctrl littermates. Spinal cords were analyzed at day 19 post-MOG/EAE.

b IHC showing increased phosphorylated p65 (NF-kb activation) in NFkB CA mice. Scale bar: 50µm.

c Representative immunofluorescence of SERPINA3N, SOX10, and CC1 in spinal cord white matter of NFkB CA and NFkB Ctrl mice. Scale bar: 20µm.

d-g Quantification of SERPINA3N expression. **d**, $P = 0.969$; **e**, $P = 0.1618$; **f**, * $P = 0.0180$; **g**, * $P = 0.0222$. $n = 3$ mice per group. Statistical significance was determined using a two-tailed unpaired Student's t-test.

Data are presented as mean values ± SEM. Source data are provided as a Source Data file.

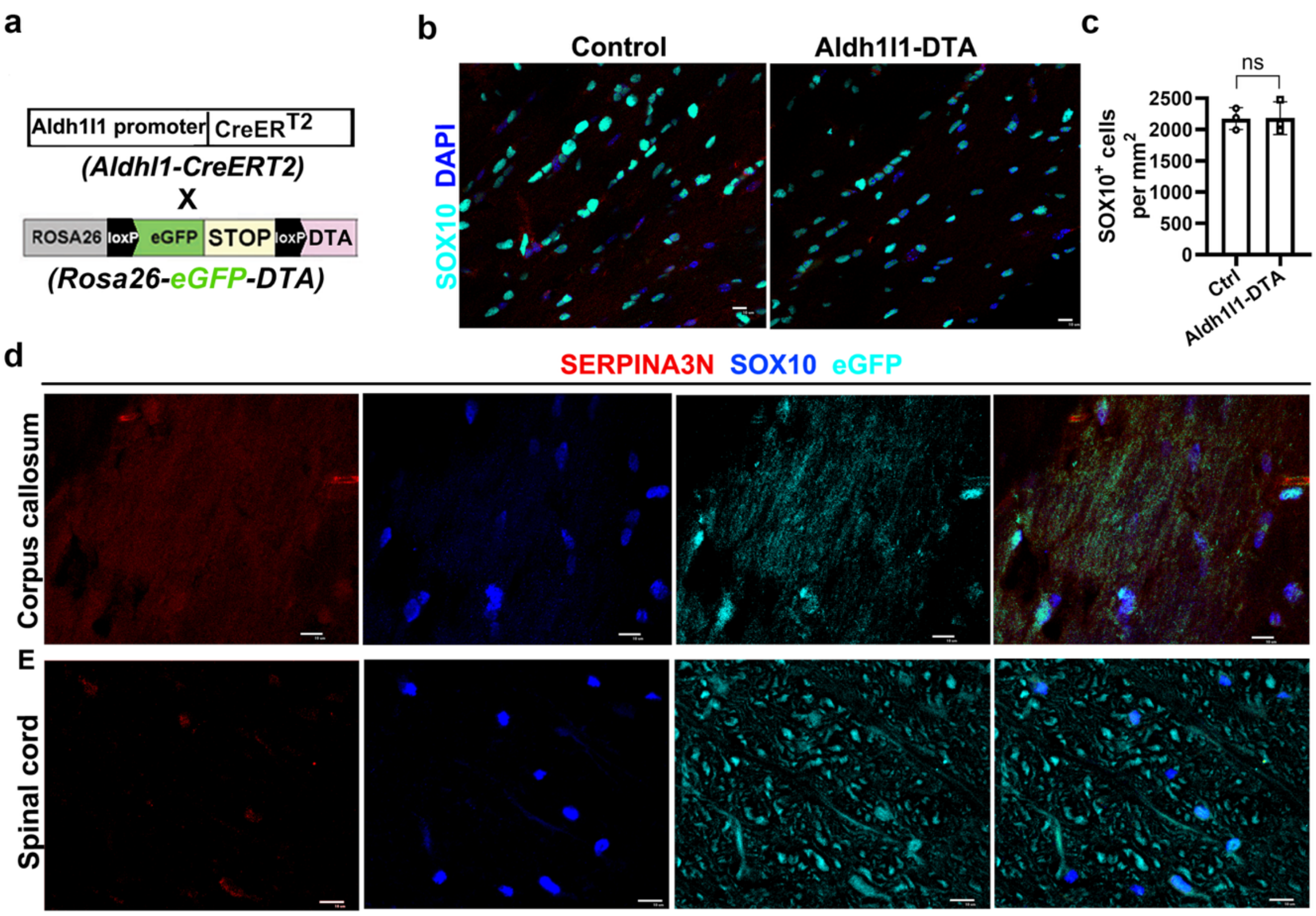

Supplementary Figure 11 – Absence of SerpinOLs in the brain and spinal cord of Aldh1l1-DTA mice (related to Figure 6).

a Astrocyte-specific Cre line (*Aldh1l1-CreER*^{T2}) was crossed with *Rosa26-eGFP-DTA* mice to generate *Aldh1l1-CreER*^{T2}: *Rosa26-eGFP-DTA* (Aldh1l1-DTA) mice. Tamoxifen treatment induces Cre-mediated eGFP silence and DTA expression specifically in astrocytes. A single tamoxifen injection (1mg) was given to adult Aldh1l1-DTA or *Aldh1l1-CreER*^{T2} Ctrl mice. Brain/spinal cord were analyzed 24 hours after tamoxifen injection.

b-c Fluorescent IHC and quantification of SOX10^{+} oligodendroglial cells in the corpus callosum. P = 0.9670. Scale bars;10 μm. n = 3 mice per group. Statistical significance was determined using a two-tailed unpaired Student's t-test.

d Triple IHC of SERPINA3N, SOX10, and eGFP in the corpus collosum and spinal cord of Aldh1l1-DTA mice. Scale bars: 10 μm.

Data are presented as mean values ± SEM. Source data are provided as a Source Data file.

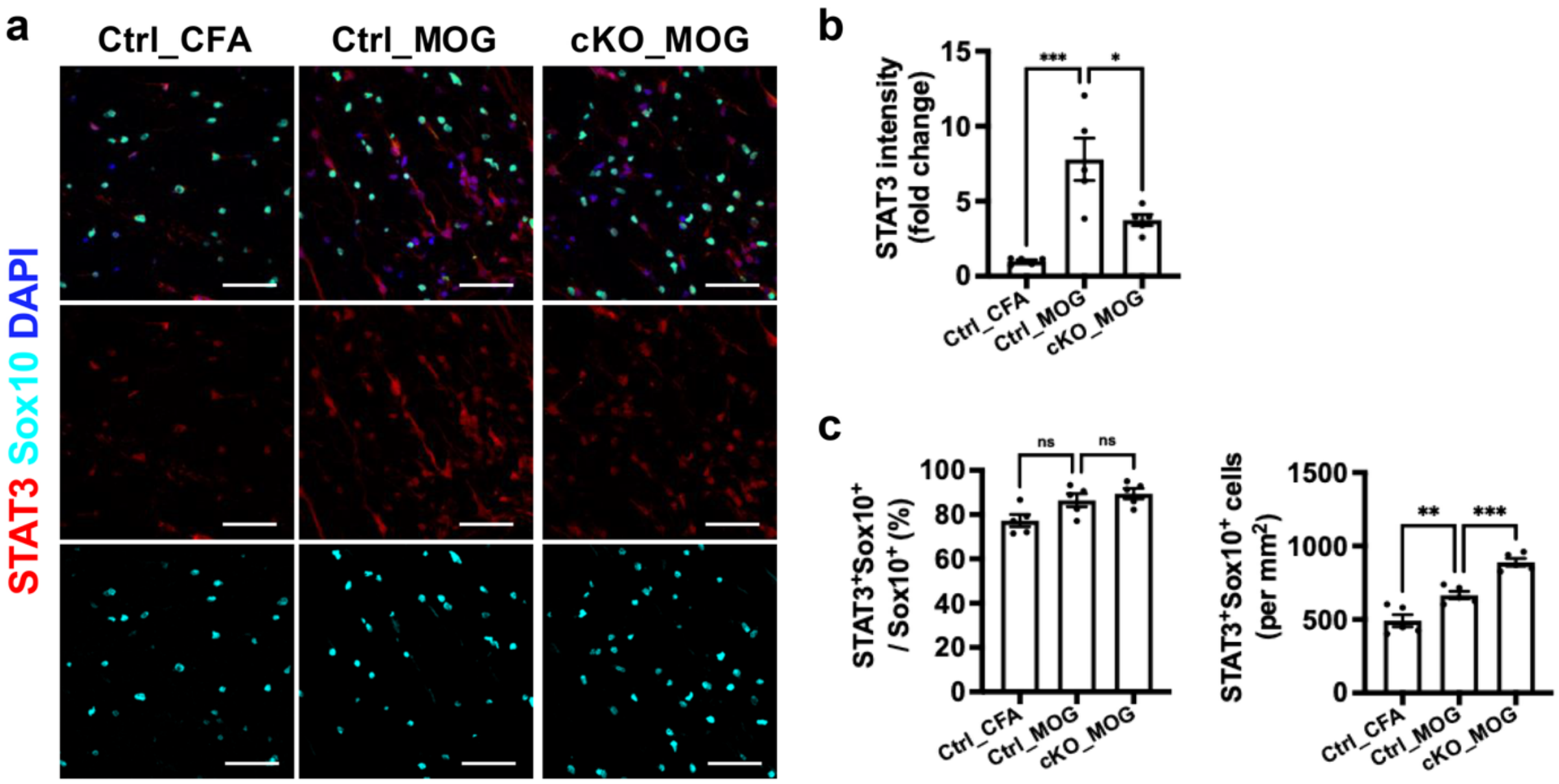

Supplementary Figure 12 – *Serpina3n* deficiency attenuates STAT3 signaling intensity in the spinal cord during EAE (related to Figure 7).

a Double staining for STAT3 and Sox10 in the spinal cords from Ctrl and *Serpina3n* cKO mice under CFA or MOG-induced EAE conditions. STAT3 is broadly expressed throughout the spinal cord and markedly increased during EAE.

b Quantification of STAT3 signal intensity. MOG immunization significantly increases STAT3 intensity, and this induction is partially reduced in *Serpina3n* cKO mice, without an obvious change in regional distribution. *** P = 0.0002, * P = 0.0108. n = 5 mice per group.

c Quantification of $STAT3^+$ $SOX10^+$ cells. The percentage of $SOX10^+$ cells that are $STAT3^+$ is comparable between Ctrl_MOG and cKO_MOG mice, whereas the density of $STAT3^+SOX10^+$ cells is increased in both EAE groups relative to CFA controls. $STAT3^+Sox10^+$ / $Sox10^+$ P = 0.0642 (Ctrl_CFA vs. Ctrl_MOG); P = 0.6978 (Ctrl_MOG vs. cKO_MOG). $STAT3^+Sox10^+$ cells: ** P = 0.0047, *** P = 0.0007. n = 5 mice per group.

Statistical significance was determined using one-way ANOVA followed by Tukey's multiple comparisons test. Scale bars: 50 µm.

Data are presented as mean values ± SEM. Source data are provided as a Source Data file.

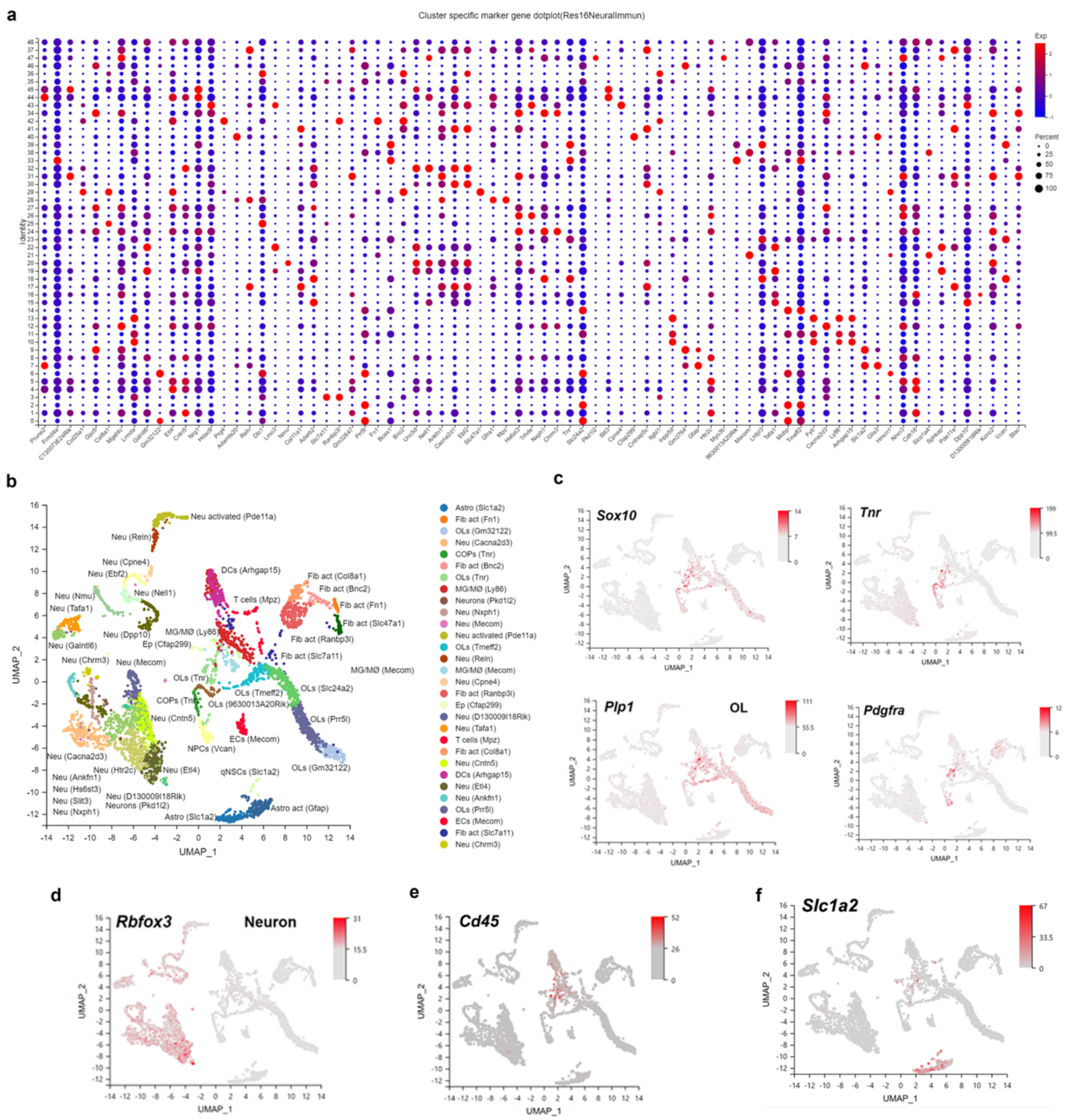

Supplementary Figure 13 – lineage-specific marker expression in UMAP clusters of scRNA-seq (related to Figure 7).

a Dot plot visualizing the expression of canonical marker genes used to assign cell identity to each scRNA-seq cluster. The size of the dot represents the percentage of cells expressing the gene, and the color intensity represents the average expression level (red = high, blue = low). A distinct cluster of fibroblast-like cells was identified, characterized by the enriched expression of *Col8a1, Bnc2, Fn1, Slc47a1, Ranbp3l,* and *Slc7a11*. These cells likely represent meningeal and perivascular fibroblasts captured during the dissociation of the whole spinal cord. Cluster identities were assigned using canonical markers referenced in the CellMarker 2.0 database.

b Uniform Manifold Approximation and Projection (UMAP) visualization of cell-type annotations for scRNA-seq performed on day 30 (D30) MOG spinal cords. Major cell populations are labeled with representative marker genes.

c Feature plots showing expression and distribution of stage-related oligodendroglial lineage markers (*Sox10, Tnr, Plp1, and Pdgfra*). Cells annotated as oligodendrocytes exhibit high *Sox10* and *Plp1* expression.
d Feature plot showing the expression level and distribution of the pan-neuronal marker gene *Rbfox3* (NeuN).
e Feature plot showing the expression level and distribution of the immune cell marker gene *Cd45*.
f Feature plot showing the expression level and distribution of the astrocyte marker gene *Scl1a2* (glutamate transporter 1, GLT1).

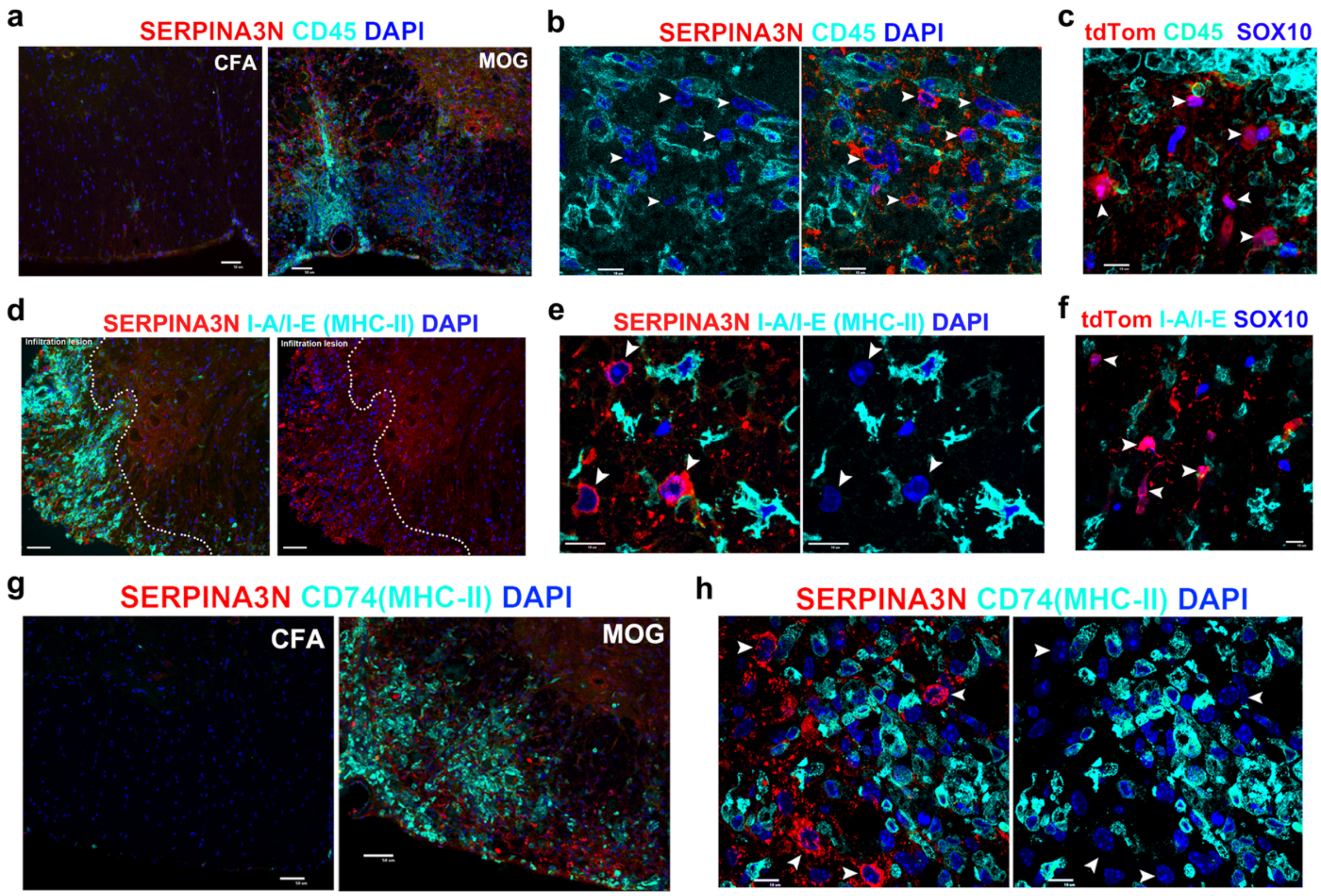

Supplementary Figure 14 – SerpinOLs do not adopt a classical immune or antigen-presenting phenotype in inflammatory demyelination (related to Figure 7).

a-b Confocal images of low (**a**) and high (**b**) magnification showing that SERPINA3N$^+$ cells (arrowheads) are negative for immune cell marker CD45.

c Triple IHC showing that tdTom$^+$SOX10$^+$ SerpinOLs (arrowheads) are negative for CD45 in Serpina3n-tdTom reporter mice.

c-e Confocal images of low (**d**) and high (**e**) magnification showing that SERPINA3N$^+$ cells (arrowheads) were negative for major histocompatibility complex class II (MHC-II) molecule I-A/I-E.

f Triple IHC showing that the vast majority of tdTom$^+$SOX10$^+$ SerpinOLs (arrowheads) do not express MHC-II molecule I-A/I-E in Serpina3n-tdTom reporter mice.

g-h Confocal images of low (**g**) and high (**h**) magnification showing that SERPINA3N$^+$ cells (arrowheads) are negative for MHC-II molecule CD74.

Adult Serpina3n-tdTom mice or WT mice were injured by MOG/EAE, and the lumbar spinal cord ventral white matter was analyzed by immunofluorescent staining at day 21 post-MOG/EAE. Scale bars: 50 μm **a, d, g**; 10 μm **b, c, e, f, h**.

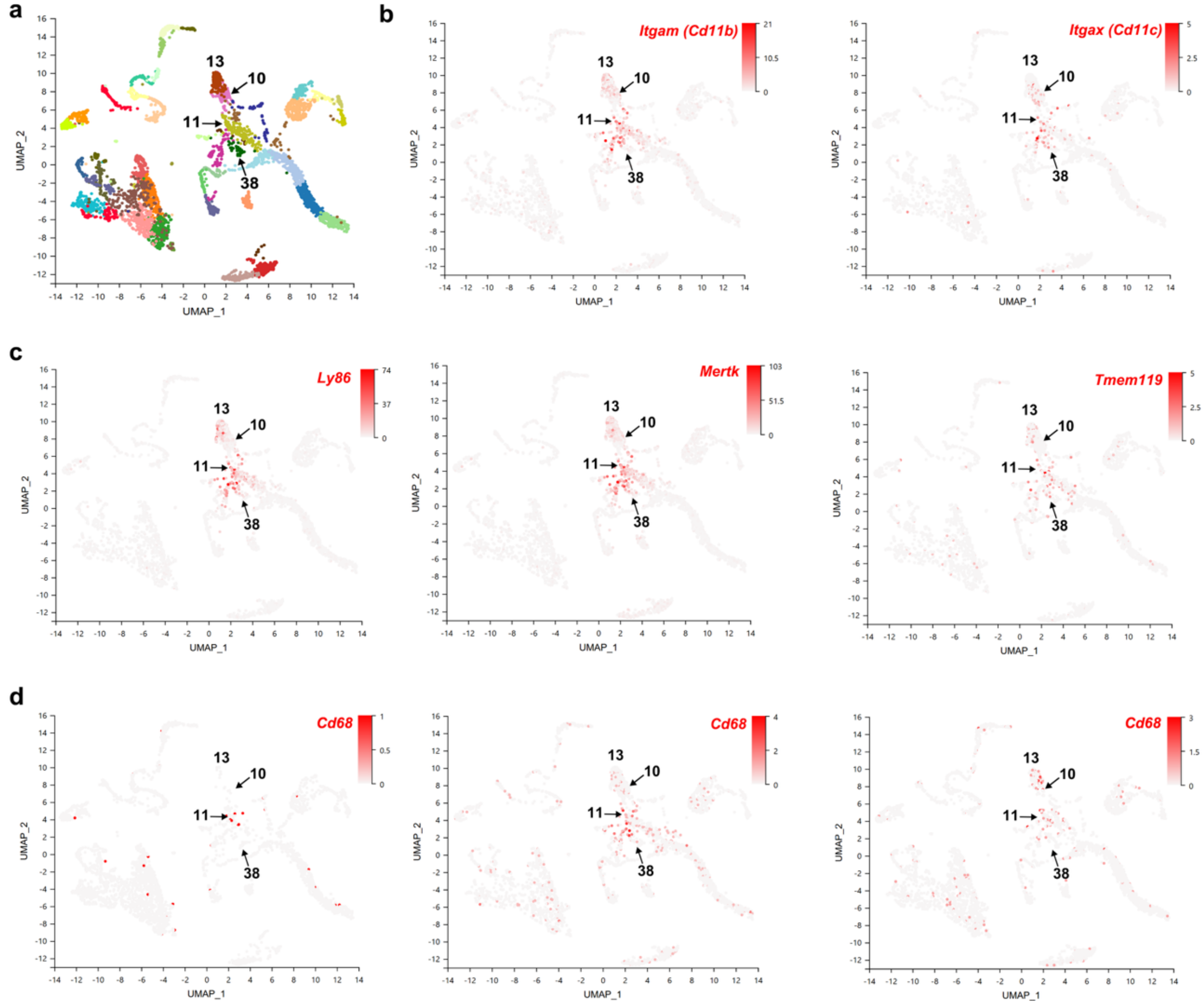

Supplementary Figure 15 – Myeloid cell clusters of scRNA-seq of MOG/EAE (related to Figure 8).

a Cluster distribution of myeloid cells. Cluster 11, 13, and 38 were annotated as microglia/macrophages (MG/MØ), and cluster 10 as dendritic cells (DCs).

b Distribution and expression level of myeloid cell gene *Cd11b* and DC-enriched gene *Cd11c*.

c Distribution and expression level of activated myeloid cell genes *Ly86* and *Mertk*, and microglial gene *Tmem119*.

d Distribution and expression level of the activated microglia/macrophage marker *Cd68* in CFA (left), Ctrl_MOG (middle), and *Serpina3n* cKO_MOG (right) mice.

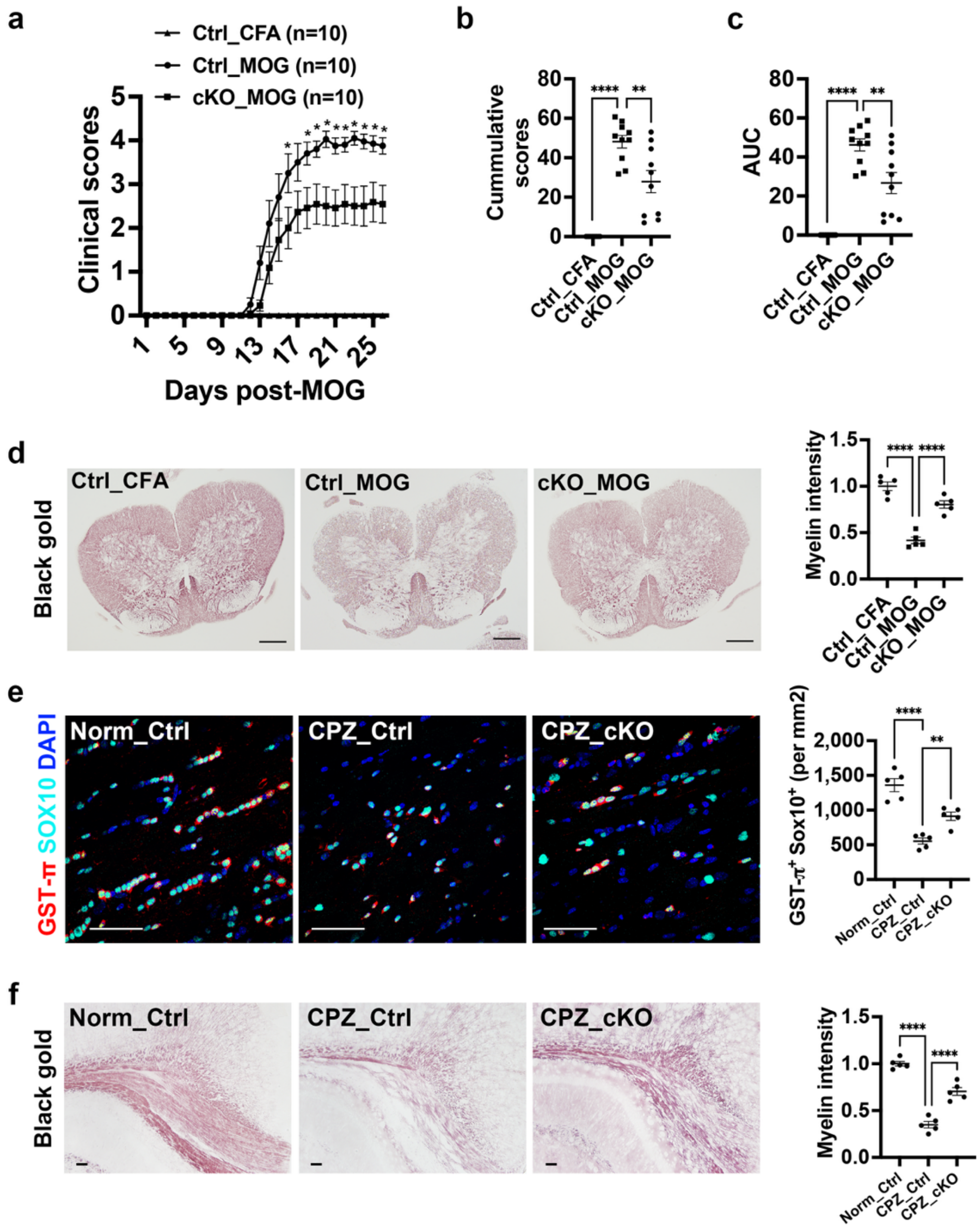

Supplementary Figure 16 – Serpina3n deficiency attenuates clinical severity and prevents demyelination in EAE and cuprizone models (related to Figure 8).

a-c Clinical score in the MOG-induced EAE model. Following MOG immunization, Ctrl_MOG mice developed progressive and severe motor deficits, whereas Serpina3n cKO_MOG (*Sox10-Cre:Serpina3n$^{fl/fl}$*) mice exhibited significantly attenuated disease (**a**). This protection was supported by reduced cumulative clinical scores (**b**) and a decreased area under the curve (AUC) (**c**) compared with Ctrl_MOG controls. EAE clinical score, Ctrl_CFA vs. Ctrl_MOG: D15 * $P = 0.0128$; D16 * $P = 0.0464$; D17 * $P = 0.0135$; D18 * $P = 0.0121$; D19 *** $P = 0.004$; D20 ** $P = 0.0015$; D21 ** $P = 0.0035$; D22 *** $P = 0.0002$; D23 *** $P = 0.0007$; D24 ** $P = 0.0046$; D25 ** $P = 0.0048$. Cummulative score: **** $P < 0.0001$, ** $P = 0.0013$. AUC: **** $P < 0.0001$, ** $P = 0.0014$. $n = 10$ mice per group.

d Spinal cord demyelination in EAE. Black Gold myelin staining revealed extensive demyelination in Ctrl_MOG spinal cords, whereas Serpina3n cKO_MOG mice showed preserved myelin staining and significantly higher myelin signal intensity, indicating protection from inflammatory demyelination. **** $P < 0.0001$. $n = 5$ mice per group.
e-f CPZ-induced demyelination model. CPZ treatment caused a marked loss of mature OLs (GST-π$^+$Sox10$^+$) in CPZ_Ctrl mice, while *Serpina3n* cKO_CPZ (*Sox10-Cre:Serpina3n$^{fl/fl}$*_CPZ) mice retained a significantly higher density of surviving mature OLs (**e**). Consistently, Black Gold staining showed significantly higher myelin intensity in the corpus callosum of CPZ_cKO mice compared with CPZ_Ctrl (**f**). GST-π$^+$Sox10$^+$: ** $P = 0.0068$, **** $P < 0.0001$. $n = 5$ mice per group.
Collectively, these data demonstrate that *Serpina3n* deficiency protects against demyelination and preserves mature OLs in both autoimmune (EAE) and toxic (CPZ) models of CNS injury.
Statistical significance was determined using one-way ANOVA followed by Tukey's multiple comparisons test. Scale bars: 500 μm **d** and 50 μm **e, f**.
Data are presented as mean values ± SEM. Source data are provided as a Source Data file.

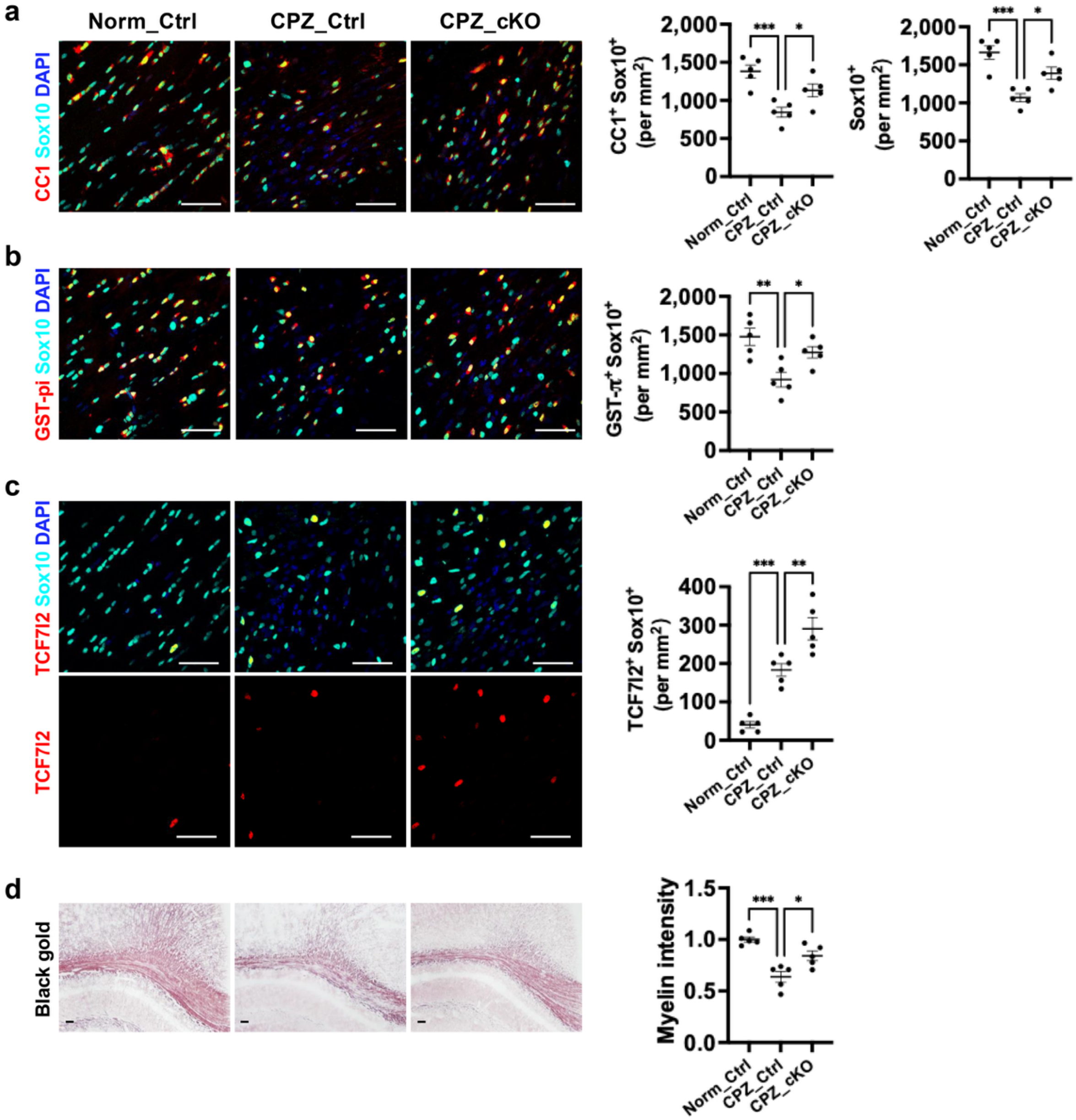

Supplementary Figure 17 – *Serpina3n* deficiency promotes CNS remyelination (related to Figure 8).

a-b Oligodendrocyte differentiation and recovery during remyelination (5 weeks CPZ diet followed by 1 week normal diet). IHC was performed using two mature OL markers, CC1 (**a**) and GST-π (**b**), co-stained with the lineage marker Sox10. As expected, CPZ_Ctrl showed partial recovery but retained reduced densities of mature OLs ($CC1^{+}Sox10^{+}$ and GST-π^{+} $Sox10^{+}$) and the total OL lineage cells ($Sox10^{+}$) compared with Norm_Ctrl. In contrast, *Serpina3n* cKO (*Sox10-Cre:Serpina3n*$^{fl/fl}$) mice exhibited significantly increased densities of both mature OLs and total $Sox10^{+}$ cells, consistent with accelerated differentiation and lineage recovery during remyelination. $CC1^{+}Sox10^{+}$: * P = 0.0486, *** P = 0.0008. $Sox10^{+}$: * P = 0.0209, *** P = 0.0002. GST-π^{+} $Sox10^{+}$: * P = 0.0431, ** P = 0.0028. n = 5 mice per group.

c Newly generated OLs assessed by TCF7l2 (transcription factor 7-like 2). CPZ induced TCF7l2 expression in $Sox10^{+}$ cells in CPZ_Ctrl mice, reflecting an endogenous repair response, which was significantly enhanced in *Serpina3n* cKO mice, as shown by a higher density of $TCF7L2^{+}$ cells. ** P = 0.005, *** P = 0.0005.

d Myelin integrity assessed by Black Gold staining. CPZ_cKO mice displayed significantly higher myelin staining intensity in the corpus callosum compared with CPZ_Ctrl. * $P = 0.0217$, *** $P = 0.0002$.

Collectively, these data demonstrate that Serpina3n deficiency promotes the formation of newly OLs, promotes their differentiation into mature myelinating cells, and ultimately enhances CNS remyelination. Statistical significance was determined using one-way ANOVA followed by Tukey’s multiple comparisons test. Scale bars: **a-d** 50 μm.

Data are presented as mean values ± SEM. Source data are provided as a Source Data file.

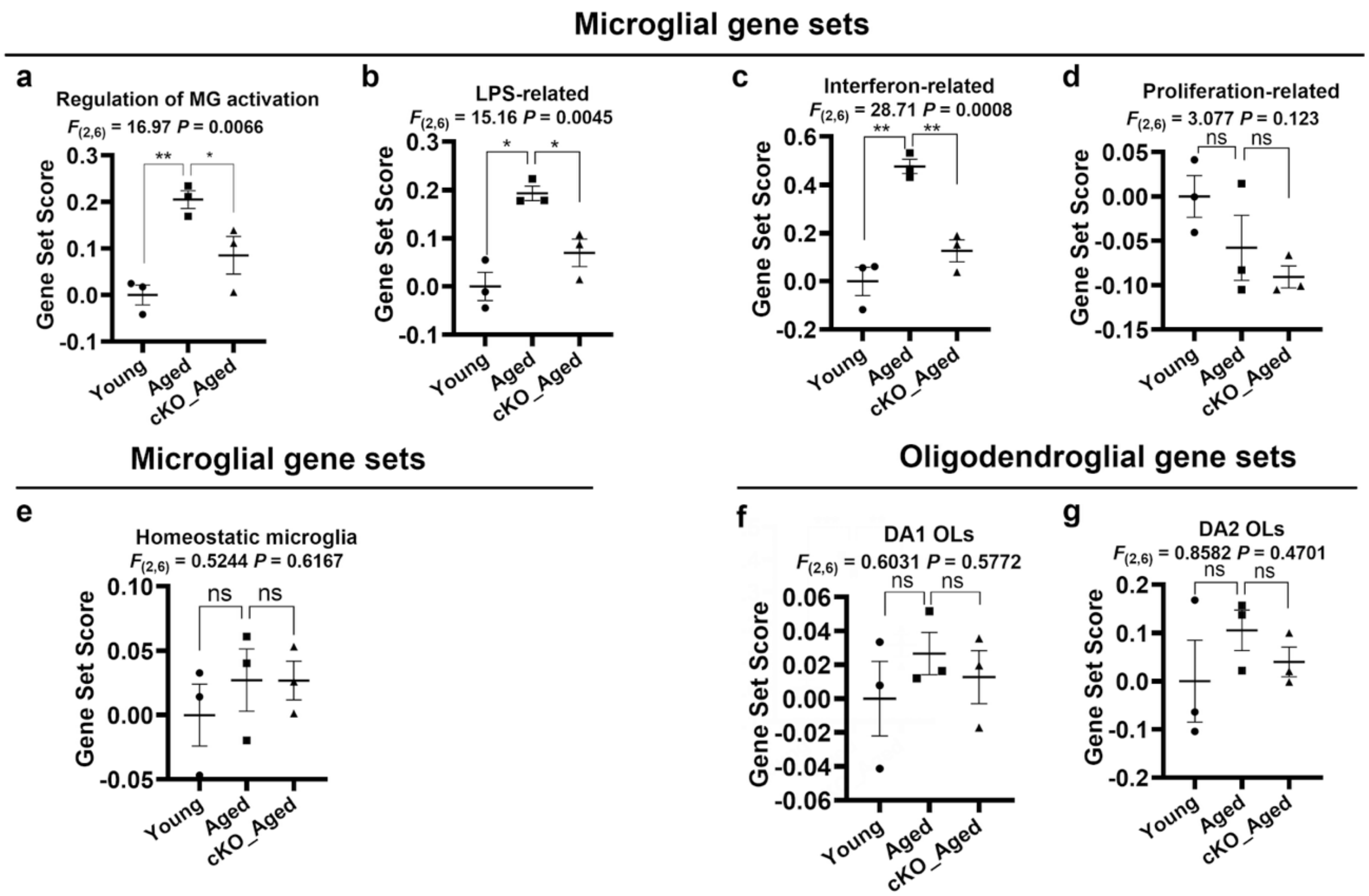

Supplementary Figure 18 - Ablating SERPINA3N alters the activation state of microglia and oligodendrocytes at the transcriptomic level (related to Figure 9).

a Cumulative gene set scores of microglial activation regulation (Supplementary Data 11) in each group of mice. ** P = 0.0069 Aged vs Young, * P = 0.0497 cKO_Aged vs Aged.

b-d Cumulative scores of microglial gene sets [60] involved in LPS-stimulation (**b**), interferon-response (**c**), and proliferation (**d**) (Supplementary Data 11). LPS * P = 0.0048 Aged vs Young, * P = 0.0236 cKO_Aged vs Aged; Interferon ** P = 0.001 Aged vs Young, ** P = 0.0034 cKO_Aged vs Aged; Proliferation P = 0.3090 Aged vs Young, P = 0.4102 cKO_Aged vs Aged.

e Gene set scores of microglial homeostasis markers [76] (Supplementary Data 11) of young (2 mon), aged (20 mon), and *Serpina3n* cKO aged (20 mon) mice. P = 0.7911 Aged vs Young, P = 0.9918 cKO_Aged vs Aged.

f-g Cummulative gene set scores of DA1 OLs (associated with immunogenic genes) and DA2 OLs (associated with cell survival and differentiation genes) (Supplementary Data 11). DA1 OLs P = 0.6777 Aged vs Young, P = 0.8288 cKO_Aged vs Aged; DA2 OLs P = 0.5649 Aged vs Young, P = 0.6974 cKO_Aged vs Aged. n = 3 mice per group (**a-g**). Statistical significance was determined using one-way ANOVA followed by Tukey's multiple comparisons test. Data are presented as mean values ± SEM. Source data are provided as a Source Data file.

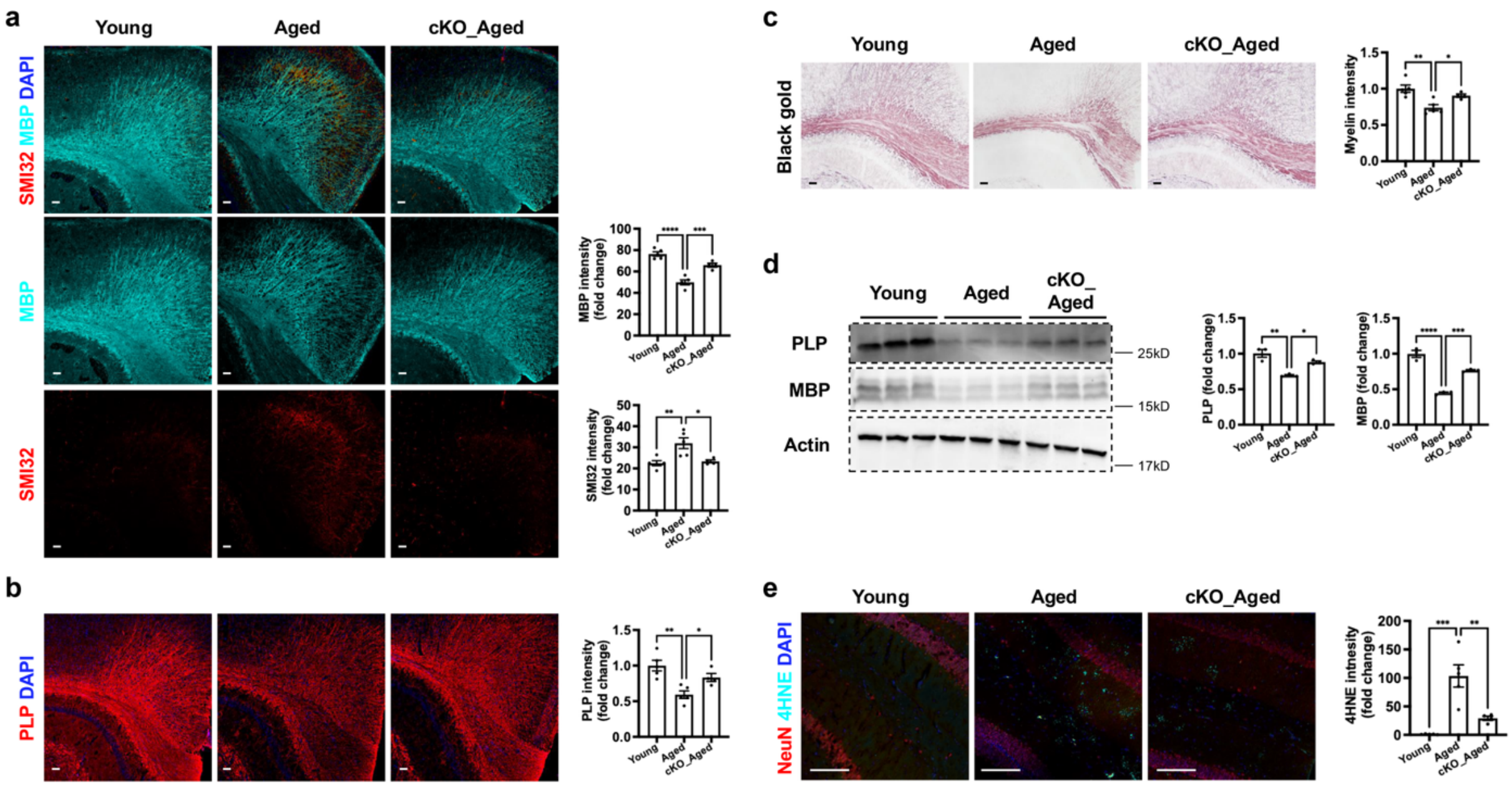

Supplementary Figure 19 – *Serpina3n* deficiency mitigates age-related white matter injury, axonal dysfunction, and oxidative stress (related to Figure 9).

a-c White matter integrity and axonal health in the corpus callosum of Young, Aged, and *Serpina3n* cKO_Aged mice. IHC for myelin basic protein (MBP) and the damaged axon marker SMI32 (**a**), as well as proteolipid protein (PLP) (**b**), showed that Aged mice exhibit reduced myelin signal (MBP and PLP) and increased axonal stress (SMI32) compared with Young controls; these age-associated changes were significantly attenuated in *Serpina3n* cKO_Aged mice. Consistently, Black Gold staining (**c**) showed preservation of myelinated fiber density in *Serpina3n* cKO_Aged mice relative to Aged controls. MBP: **** $P < 0.0001$, *** $P = 0.0006$. SMI32: ** $P = 0.0052$, * $P = 0.0140$. PLP: ** $P = 0.0013$, * $P = 0.0478$. Myelin: ** $P = 0.0019$, * $P = 0.0418$. $n = 5$ mice per group for Young and Aged; $n = 4$ mice for cKO_Agedz

d Western blot showing that the age-related reduction of PLP and MBP protein levels is partially rescued in *Serpina3n* cKO_Aged mice. β-actin was run on a separate gel using the same samples and is shown as a sample processing control. PLP: ** $P = 0.0013$, * $P = 0.0159$. MBP: **** $P < 0.0001$, *** $P = 0.0007$. $n = 3$ mice per group.

e Oxidative stress in the hippocampus. Lipid peroxidation, a hallmark of oxidative stress, was assessed by 4-Hydroxynonenal (4-HNE) staining with NeuN co-labeling. Aged mice showed increased 4-HNE accumulation compared with Young controls, which was significantly reduced in *Serpina3n* cKO_Aged mice. *** $P = 0.0002$, ** $P = 0.0031$. $n = 5$ mice per group for Young and Aged; $n = 4$ mice for cKO_Aged.

Statistical significance was determined using one-way ANOVA followed by Tukey's multiple comparisons test. Scale bars: **a-e** 50 μm.

Data are presented as mean values ± SEM. Source data are provided as a Source Data file.

Uncropped scans of all blots and gels in Supplementary Figures

Supplementary Figures 2a

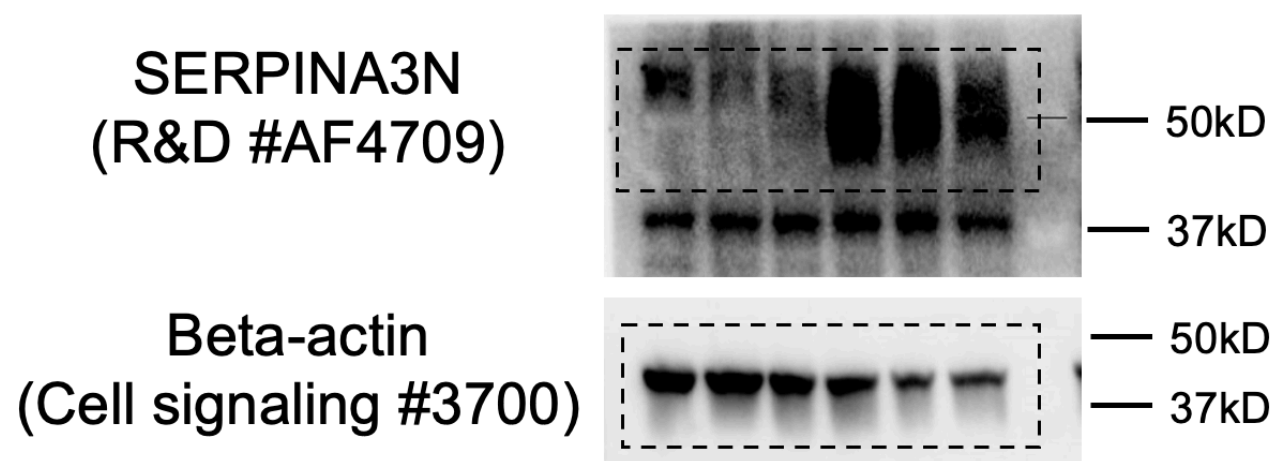

Supplementary Figures 7a

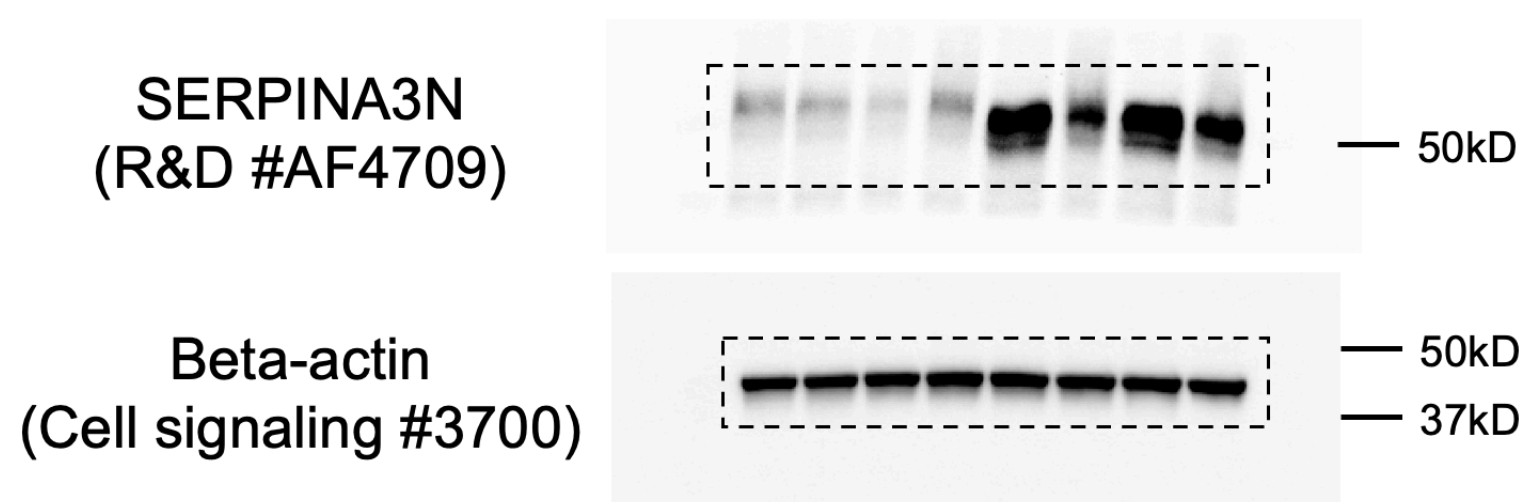

Supplementary Figures 19d

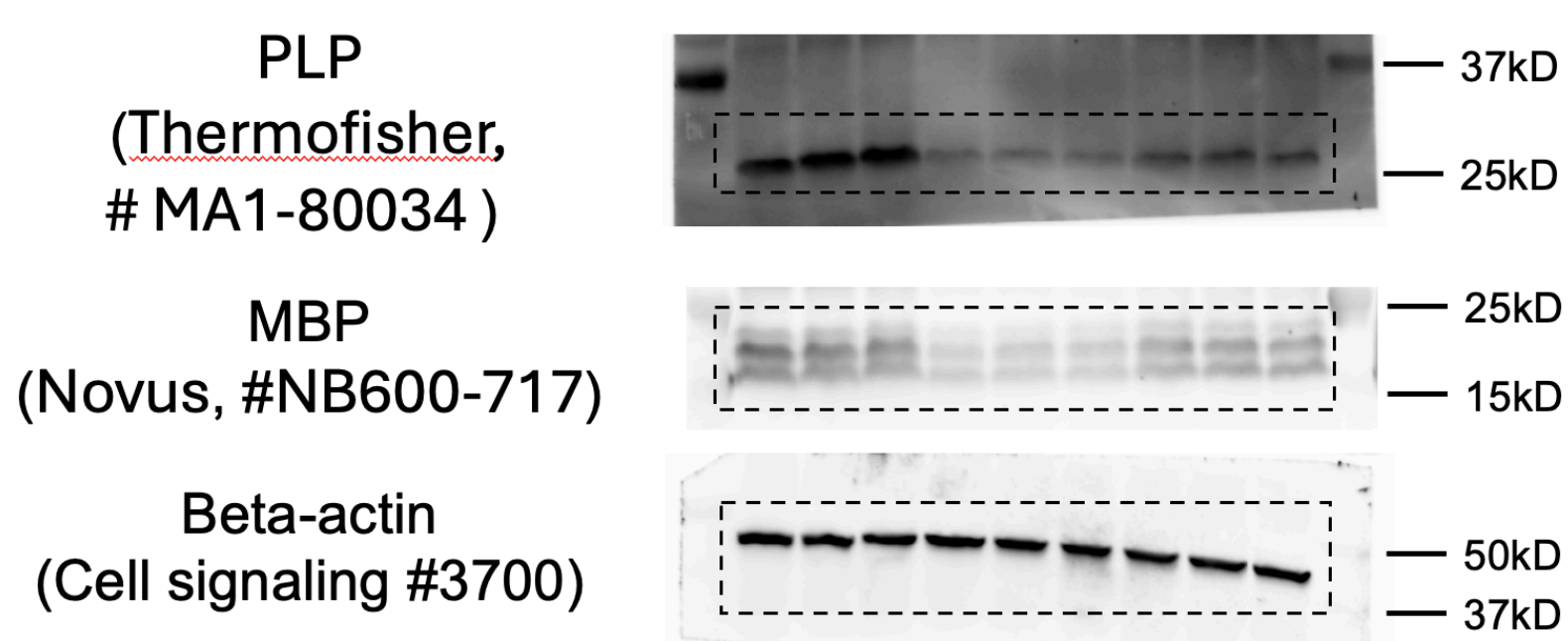

Supplement: Supplementary file 3 [file 41467_2026_76159_MOESM1_ESM.pdf]
