# Supplementary material for: Injury-transduced oligodendrocytes modulate neuroinflammation and glial activation in diseased and non-diseased central nervous system
Source: Nat Commun. 2026 Aug 29;17:10347. doi: 10.1038/s41467-026-76159-2 (PMC13623841; doi:10.1038/s41467-026-76159-2)
Supplement: Supplementary file 4 [file 41467_2026_76159_MOESM2_ESM.pdf]

Description of Additional Supplementary Files

Supplementary Data 1 - Expression levels (FPKM) of differentially expressed genes (DEGs) in the spinal cord at D36 post-EAE (related to Fig. 1).

Supplementary Data 2 - Glial type-enriched differentially expressed genes (DEGs) in response to MOG-EAE injury (related to Fig. 1).

Supplementary Data 3 - RNA-seq of MACS-purified MG, OL and Astro in CPZ-treated brain (related to Fig. 1)

Supplementary Data 4 - DEGs and functional annotations in the spinal cord of CFA versus PBS (related to Fig. 5).

Supplementary Data 5 - DEGs and functional annotations in the optic nerve of NFkB CA versus NFkB Ctrl (related to Fig. 5).

Supplementary Data 6 - Marker genes used for UMAP clustering of EAE spinal cord scRNAseq (related to Fig. 8).

Supplementary Data 7 - DEGs and functional annotation of SerpinOLs versus non-SerpinOLs in MOG-EAE (related to Fig. 8).

Supplementary Data 8 - Pseudobulk DEGs and functional annotations of microglia and macrophages in the spinal cord of Ctrl_MOG vs Ctrl_CFA (related to Fig. 9).

Supplementary Data 9 - Pseudobulk DEGs and functional annotations of microglia and macrophages in the spinal cord of cKO_MOG vs Ctr_MOG (related to Fig. 9).

Supplementary Data 10 - DEGs in microglia and GO terms of CPZ_cKO, CPZ_Ctrl, and Norm_Ctrl mice (4 weeks CPZ) (related to Fig. 9).

Supplementary Data 11 - List of gene sets and gene symbols in each gene set (related to Fig. 9 and Fig. 10).

Supplementary Data 12 – DEGs in the brain of young, aged, and cKO-aged mice (related to Fig. 10).
